# Solvent‐Induced Structural Modulation in Nanoscale Covalent Organic Frameworks Enables High‐Performance NO_2_ Sensing

**DOI:** 10.1002/anie.2684732

**Published:** 2026-08-13

**Authors:** Yu Pan, Xueying Kong, Guangling Liang, Samson Afewerki, Chao Xu

**Affiliations:** ^1^ State Key Laboratory of Structural Chemistry Fujian Institute of Research on the Structure of Matter Chinese Academy of Sciences (CAS) Fuzhou China; ^2^ University of Chinese Academy of Sciences (UCAS) Beijing China; ^3^ Division of Nanotechnology and Functional Materials Department of Materials Science and Engineering Uppsala University Uppsala Sweden; ^4^ Department of Chemistry United Arab Emirates University Al Ain UAE

**Keywords:** chemiresistive sensing, covalent organic frameworks, nitrogen dioxide sensing, porphyrin, structural modulation

## Abstract

Covalent organic frameworks (COFs) offer exceptional structural and functional tunability, enabling performance optimization in separation, catalysis, and sensing applications. Here, we report a solvent‐induced strategy for inducing structural disorder in porphyrin‐based COF nanoparticles (nanoCOFs) synthesized in aqueous acetic acid, where the porphyrin units are protonated during the synthesis. Subsequent post‐treatment with polar organic solvents induces deprotonation of the porphyrin units and effectively reduces the crystallinity of the nanoCOFs. The decreased crystallinity arises from solvent insertion between COF layers, which disrupts the ordered interlayer stacking and promotes structural disorder. Systematic control experiments demonstrated that the protonation‐deprotonation behaviors of porphyrin units, the strength of interlayer interactions, and solvent properties (e.g., size and polarity) are key factors in regulating the framework structure and degree of structural disorder. Despite the significantly decreased surface area and reduced crystallinity, the solvent‐treated nanoCOF‐366, with its less compactly stacked layers, exposes more accessible porphyrin sites to guest molecules. As a result, they exhibit enhanced NO_2_ uptake and superior chemiresistive sensing performance, achieving an ultrahigh record response of 1083.0 to 10 ppm NO_2_ and an ultralow limit of detection of 0.21 ppb. This work presents an effective strategy for structural regulation of COFs toward advanced gas sensing applications.

## Graphical abstract

Solvent intercalation disrupts the stacking order of protonated porphyrin‐based 2D COF nanoparticles, resulting in a less compactly packed structure with increased accessibility of porphyrin sites. This structural modulation leads to significantly enhanced chemiresistive NO_2_ sensing performance.

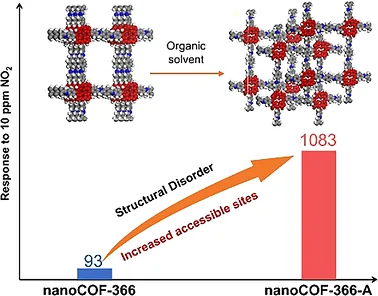

## 1 Introduction

Covalent organic frameworks (COFs) are constructed by linking purely organic monomers through strong covalent bonds in an ordered manner, forming extended networks with well‐defined porous channels [1, 2]. Guided by the principles of reticular chemistry and enabled by the powerful toolbox of organic and polymer chemistry, COFs exhibit exceptional synthetic diversity [3, 4]. Their structures (e.g., linkages, topology, and porosity) and functionalities (e.g., physical and chemical properties) can be precisely tuned at the molecular level through the rational selection of organic building blocks, polymerization reactions, and post‐synthetic modifications [5, 6, 7, 8, 9, 10]. In addition, modulation of the stacking and arrangement of two‐dimensional (2D) layers provides alternative strategies for tailoring COF structures. As a result, COFs with tunable structures and properties show great potential in applications such as energy storage and conversion, sensing, separation, and catalysis [11, 12, 13, 14, 15, 16, 17, 18, 19, 20, 21].

2D COFs consist of covalently bonded planar layers that stack through interlayer noncovalent interactions (e.g., van der Waals forces and *π*–*π* stacking) to form extended porous channels. Modulation of the arrangement of these 2D layers provides an effective strategy to tune the structures and properties of 2D COFs, thereby enabling extended and diversified applications. Various approaches have been developed to regulate the stacking modes of 2D COFs [6, 22, 23, 24]. For instance, by controlling the synthesis conditions, the stacking arrangement can be tuned between eclipsed and staggered modes, which directly affects the effective pore size and pore connectivity, enabling efficient molecular separation applications [22]. In addition, the insertion of guest molecules, such as gas and solvent molecules, into the interlayer space can weaken *π*–*π* interactions and induce layer slipping or lattice expansion, allowing reversible control over porosity and framework accessibility [25, 26, 27, 28, 29]. Furthermore, chemical exfoliation, mechanical delamination, or electrochemical delamination of layered COFs into few‐layer or monolayer nanosheets effectively disrupts the long‐range stacking order, significantly increasing the accessible surface area and improving mass transport, thereby enhancing their performance in catalysis, energy storage, and sensing [24, 30, 31, 32, 33, 34, 35, 36, 37]. In addition, the resulting exfoliated COF nanosheets, with their excellent solution processability, enable the fabrication of freestanding membranes for advanced separation applications [8, 38]. These stacking‐modulation strategies offer powerful opportunities to engineer COF structures and unlock new functionalities for advanced applications.

Among the various 2D COFs, porphyrin‐based COFs constitute a particularly important class owing to the unique structural and functional characteristics of porphyrin units [39, 40]. Porphyrins are large, rigid, and highly *π*‐conjugated macrocycles that exhibit strong visible‐light absorption, rich redox chemistry, and versatile metal‐coordination capability. These attributes render porphyrin‐based COFs attractive platforms for applications in photocatalysis, electrocatalysis, sensing, gas adsorption, and energy conversion [41, 42, 43]. Importantly, protonation of the inner nitrogen atoms of the porphyrin macrocycle not only modulates its light‐absorption behavior and electronic structure but also offers an effective handle for morphology control and enhanced dispersibility in solution [44]. Moreover, protonation can induce electrostatic repulsion between positively charged layers, weakening interlayer noncovalent interactions and thereby providing a potential strategy to modulate interlayer stacking, introduce stacking disorder, or even promote layer delamination. Although ordered porous structures typically exhibit higher surface areas when probed by inert gases (e.g., N_2_, Ar), introducing layer slipping or disorder in COFs can expose additional adsorption sites for specific guest molecules (e.g, H_2_O, CO_2_, NO_2_), thereby increasing adsorption capacity or host–guest interactions [45, 46]. This observation inspired us to explore structural modulation in porphyrin‐based COFs as a strategy to enhance their gas‐sensing performance.

In this study, we report an organic solvent‐induced structural disorder in porphyrin‐based nanoCOFs synthesized in aqueous acetic acid, which exhibits a protonated framework and well‐defined nanorod morphology. By systematically investigating the interplay between protonation–deprotonation behavior, molecular side‐group engineering of COFs, and solvent properties, we elucidate the detailed mechanisms governing solvent‐responsive structural evolution in nanoCOFs. Remarkably, organic‐solvent‐treated nanoCOFs with reduced interlayer ordering expose more accessible porphyrin sites, resulting in significantly improved chemiresistive NO_2_ sensing performance. Furthermore, the correlation between structural transformation and enhanced gas sensing performance is elucidated at the molecular level.

## 2 Results and Discussion

### 2.1 Synthesis and Characterizations

nanoCOF‐366 was synthesized in an aqueous acetic acid solution under ambient conditions following our previously reported method, with minor modifications [44]. Briefly, terephthalaldehyde (TPA) was added dropwise to an aqueous suspension of 5,10,15,20‐tetrakis(4‐aminophenyl)porphyrin (TAPP), followed by aging at room temperature for 3 days to yield protonated nanoCOF‐366 nanorods (Figure 1a). The material exhibits excellent aqueous dispersibility due to its nanoscale morphology and protonated framework (Figure 1b,c). Statistical analysis revealed that the nanoparticles possess an average width of 56.1 ± 13.7 nm and an average length of 152.9 ± 46.3 nm (Figure S1). Powder x‐ray diffraction patterns agree well with the simulated COF‐366 structure and the lattice size obtained from high‐resolution transmission electron microscopy (HRTEM), confirming the formation of a highly crystalline framework (Figures 1d,e and S2). The polyimine network was verified by characteristic C = N signals in FTIR and solid‐state ^13^C NMR spectra (Figure 1f,g), while N_2_ sorption measurements revealed a microporous structure with a Brunauer–Emmett–Teller (BET) surface area of 1768 m^2^ g^−1^ (Figures 1h and S3). The morphology, crystallinity, chemical structure, and porosity are consistent with our previous report. Detailed synthesis procedures and characterization data are provided in the Supporting Information and our earlier study.

**FIGURE 1:**
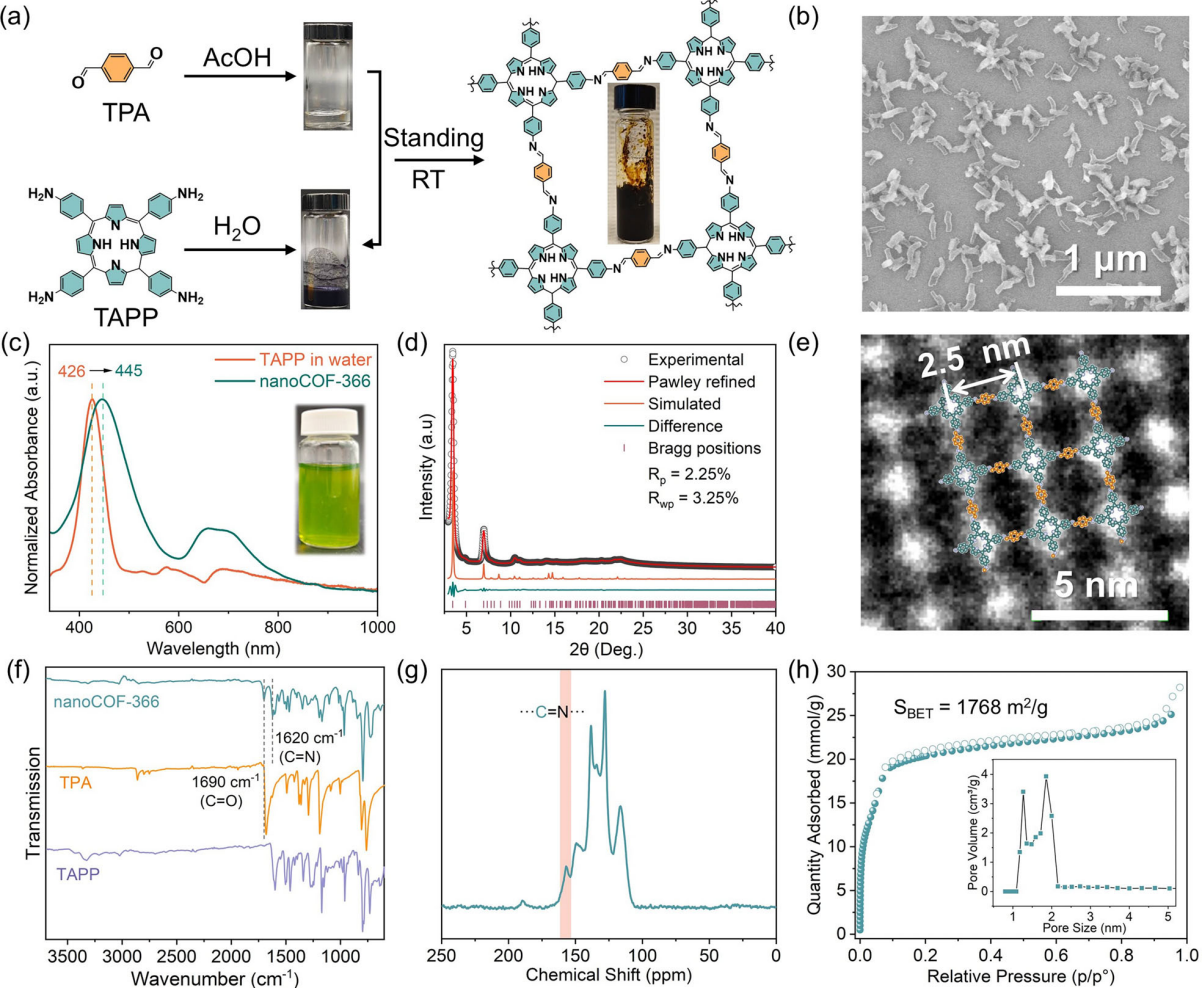
Synthesis of nanoCOF‐366. (a) Schematic illustration of the aqueous synthesis of nanoCOF‐366; (b) TEM and SEM images of as‐synthesized nanoCOF‐366; (c) UV–vis absorption spectra of TAPP and nanoCOF‐366 in water. Inset: optical image of the aqueous suspension of nanoCOF‐366; (d) comparison of the experimental PXRD pattern of as‐synthesized nanoCOF‐366 with the simulated pattern and Pawley refinement fit. (e) FFT‐HRTEM image of as‐synthesized nanoCOF‐366. (f) FT‐IR spectra of TAPP, TPA linkers, and nanoCOF‐366; (g) solid‐state ^13^C NMR spectrum of nanoCOF‐366; (h) N_2_ adsorption–desorption isotherms and corresponding pore size distribution of nanoCOF‐366.

### 2.2 Structural Modulation

The as‐synthesized nanoCOF‐366 exhibits excellent long‐term stability in water, retaining high crystallinity, high surface area, and excellent dispersibility for several months (Figures 2a, S4, and S5). In contrast, it shows poor stability in organic solvents, with significant losses in dispersibility, crystallinity, and porosity. Upon addition of acetone to an aqueous suspension of nanoCOF‐366, the material undergoes a pronounced color change from green to brown (Figure 2a). Moreover, exposure of the solid sample to acetone vapor induces this color change within seconds (Figure 2b). In addition, the acetone‐treated sample shows significantly reduced dispersibility in both water and organic solvents and rapidly precipitates from suspension (Figure S6). SEM images reveal that, although the solvent‐treated sample largely retains its rod‐like morphology, it displays blurred edges, reduced facet definition, and partial aggregation compared with the pristine nanoparticles, which exhibit well‐defined, isolated nanorods with sharp edges and corners (Figure S7). More strikingly, acetone treatment results in a dramatic loss of crystallinity. XRD measurements of as‐synthesized nanoCOF‐366 exposed to acetone vapor show a significant reduction in the intensity of the characteristic (100) and (200) diffraction peaks, together with an increased full width at half maximum (FWHM) (Figures 2c and S8). Notably, this change occurs even after exposure to acetone vapor for only a few seconds, indicating the exceptional sensitivity of the material to acetone. Meanwhile, the (001) reflection at ∼22° decreases in intensity, broadens, and shifts slightly to lower 2*θ* values after acetone treatment, indicating expanded interlayer spacing accompanied by disruption of stacking order (Figure 2c, inset and Figure S9). These structural changes are further reflected in a sharp loss of porosity, with the specific surface area decreasing rapidly from approximately 1768 to 630.6 m^2^ g^−1^ after 5 min of treatment and further to < 100 m^2^ g^−1^ after long time exposure (Figure 2d). HRTEM provides further insight into the structural changes. The as‐synthesized nanoCOF‐366, with high crystallinity, displays well‐defined periodic contrast features (Figure 2e), which progressively lose regularity upon acetone treatment, exhibiting local distortion and curvature (Figure 2f). This evolution is indicative of gradual disruption of long‐range order and increasing structural deformation. After prolonged exposure, the channel features become largely indistinct, and the contrast features eventually disappear, indicating severe disordering and a transition toward a highly disordered state (Figure 2g). Collectively, these results confirm that acetone treatment induces significant structural disorder in nanoCOF‐366, leading to loss of long‐range order and severe porosity collapse within the framework.

**FIGURE 2:**
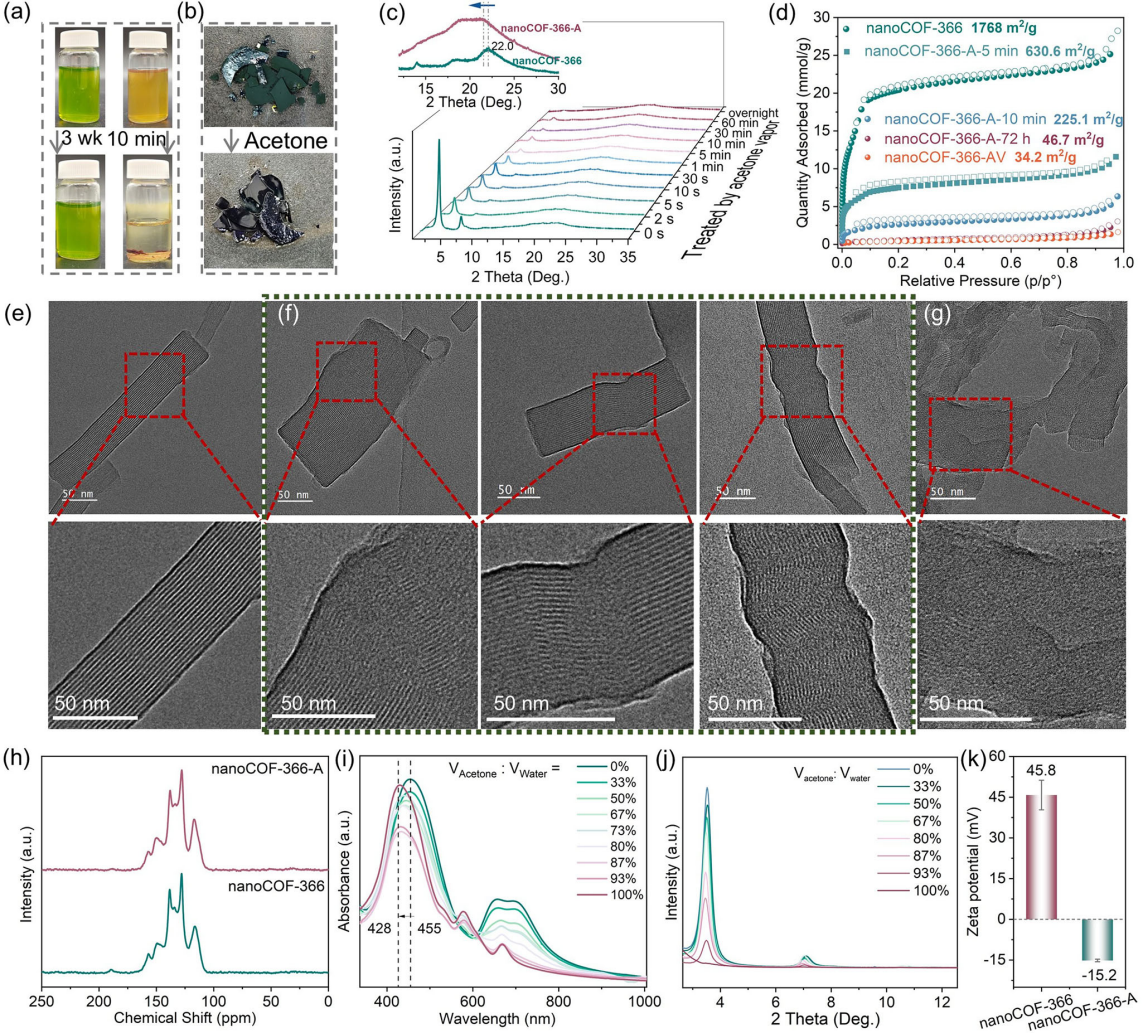
Solvent‐induced structural evolution of nanoCOF‐366. (a) Optical images of nanoCOF‐366 dispersed in water (left) and acetone (right), showing the suspensions after 3 weeks and 10 min, respectively; (b) optical images of as‐synthesized nanoCOF‐366 powder and powder treated with acetone; (c) time‐dependent PXRD of as‐synthesized nanoCOF‐366 treated with acetone vapor; inset: magnified PXRD patterns of nanoCOF‐366 and nanoCOF‐366‐A in the 2*θ* range of 11°–30°. (d) N_2_ sorption isotherms of nanoCOF‐366, the sample treated with acetone solution (nanoCOF‐366‐A) with different durations and the sample treated with acetone vapor (nanoCOF‐366‐AV); (e) HRTEM images of as‐synthesized nanoCOF‐366, nanoCOF‐366 after short‐time acetone treatment (f), and after 72 h treatment (g); (h) comparison of solid‐state ^13^C NMR spectra of nanoCOF‐366 and nanoCOF‐366‐A; (i) UV–vis spectra and (j) PXRD patterns of the corresponding samples treated with solvents of varying water/acetone ratios; (k) zeta potential of nanoCOF‐366 and nanoCOF‐366‐A.

To elucidate the structural changes induced by acetone at the molecular level, we first examined the molecular structure of nanoCOF‐366 before and after treatment. FT‐IR and ^13^C NMR spectra were nearly identical, confirming that the polyimine framework remained chemically intact (Figures 2h and S10). However, UV–vis absorption spectra of the aqueous suspension revealed gradual blue shifts in both the Soret and Q bands upon addition of acetone and with increasing the acetone content, accompanied by decreased Q‐band intensity and the emergence of multiple Q bands (Figure 2i). Eventually, the UV–vis spectrum of nanoCOF‐366 dispersed in pure acetone closely resembles that of the TAPP linker (Figure S11). These spectral changes suggest a gradual transition from protonated porphyrin species toward the neutral free‐base state upon acetone treatment. Consistently, the crystallinity of samples treated in water/acetone mixtures gradually decreases with increasing acetone fraction (Figure 2j), demonstrating the correlation between protonation–deprotonation behavior and the associated structural evolution. Furthermore, the zeta potential decreases from + 45.7 to − 15.2 mV upon acetone treatment (Figure 2k). These spectral and surface charge variations are consistent with those observed during deprotonation of nanoCOF‐366 via titration with the organic base triethylamine (TEA), which restores the neutral free‐base porphyrin form [44]. Collectively, these results provide strong evidence that acetone treatment induces deprotonation of the porphyrin cores in nanoCOF‐366, leading to recovery of the neutral free‐base porphyrin state.

Interestingly, further soaking the acetone‐treated sample in water under ambient conditions allows for partial recovery of zeta potential and crystallinity (Figures S12–S14). This recovery is likely driven by the re‐protonation of the porphyrin units in water, which restores interlayer interactions, including hydrogen bonding and electrostatic interactions with counteranions, promoting rearrangement and restacking of the COF layers into a more ordered structure.

Based on these results, we propose that protonation of the porphyrin macrocycles plays a key role in the solvent‐dependent stability of nanoCOF‐366. In water, solvation of the protonated porphyrins and associated counterions, together with hydrogen‐bonding interactions, stabilizes the tight, ordered stacking of the layers and preserves high crystallinity. When exposed to acetone, acetone molecules penetrate into the interlayer regions and exchange with associated water molecules, altering the local solvation environment of the framework. This solvent exchange facilitates partial deprotonation of the porphyrin centers, disrupting hydrogen‐bonding interactions and weakening interlayer *π*–*π* stacking and electrostatic interactions. The reduced interlayer interactions promote layer slippage and stacking disorder, ultimately resulting in decreased long‐range order, crystallinity, and porosity.

To further illustrate the mechanism of the solvent induced structure change of nanoCOFs‐366, we performed a range of comparison studies. First, solvothermally synthesized sample (ST‐COF‐366) was treated by acetone with the same method. As expected, no significant changes in either porosity or crystallinity was observed (Figures 3a,b and S15). This high stability can be attributed to strong interlayer interactions arising from its neutral framework, as evidenced by UV–vis spectroscopy, and its lower zeta potential compared to nanoCOF‐366 (Figures 3c and S16). In this neutral state, reduced electrostatic effects favor strong *π*–*π* stacking between adjacent COF sheets, along with covalent framework rigidity, which significantly enhances interlayer stacking and prevents the insertion of solvent molecules into the layered structure. As a result, ST‐COF‐366 is resistant to solvent‐induced structural disruption. Secondarily, thermal treatment of nanoCOF‐366 markedly enhances its structural stability toward acetone. As the heating temperature increases from 25°C to 400°C, the treated sample exhibits progressively enhanced stability in organic solvents. For example, after heating at 300°C and 400°C under vacuum, it shows strong resistance to acetone, with both crystallinity and porosity largely retained (Figures 3d,e, S17, and S18). This behavior can be attributed to deprotonation of the porphyrin units during drying and thermal treatment, which restores the neutral charge of the framework, strengthens *π*–*π* stacking, and thereby suppresses solvent intercalation between layers, preserving the ordered stacked structure, similar to that observed in non‐protonated ST‐COF‐366. The deprotonation process is evidenced by in situ DRIFTS measurements indicating the removal of NH^+^–counterion interactions, as well as color change and blue‐shifted UV–vis spectra (Figures 3f, S19, and 20; detailed discussions in the Supporting Information). Furthermore, we investigated the influence of organic solvent type on the stability of nanoCOF‐366. Specifically, the as‐synthesized nanoCOF‐366 was treated with acetone, methanol, ethanol, benzene, toluene, xylene, and mesitylene, respectively. All solvent‐treated samples exhibited some degree of reduced crystallinity and porosity (Figures 3g,h and S21). However, treatment with bulkier, aromatic, and less polar solvents resulted in substantially less structural degradation, yielding higher specific surface areas in the range of 400–800 m^2 ^g^−1^, compared to the much lower surface areas (100–200 m^2 ^g^−1^) observed for samples treated with small, highly polar solvents such as acetone, methanol, and ethanol (Figure 3h). This solvent‐dependent behavior can be explained by differences in solvent polarity and molecular size. Small, highly polar solvents readily intercalate into the interlayer regions of the 2D COF, effectively weakening π–π and van der Waals interactions and causing pronounced structural disruption. Similar to acetone, treatment with methanol and ethanol rapidly induced deprotonation of the porphyrin units, as evidenced by UV–vis spectroscopy and zeta potential measurements (Figures 3i and S22). In contrast, bulkier, less polar aromatic solvents are sterically hindered from intercalating between layers and exhibit weaker interactions with the framework, thereby preserving the protonated structure (Figure 3i) and the stacked order of nanoCOF‐366. Consequently, the framework maintained high crystallinity and porosity in toluene, xylene, and mesitylene over extended periods. Interestingly, although treatment with TEA induces deprotonation, the crystalline and porous structure of nanoCOF‐366 is largely preserved, likely due to the formation of Et_3_NH^+^ species that provide additional interlayer stabilization through hydrogen‐bonding and electrostatic interactions.

**FIGURE 3:**
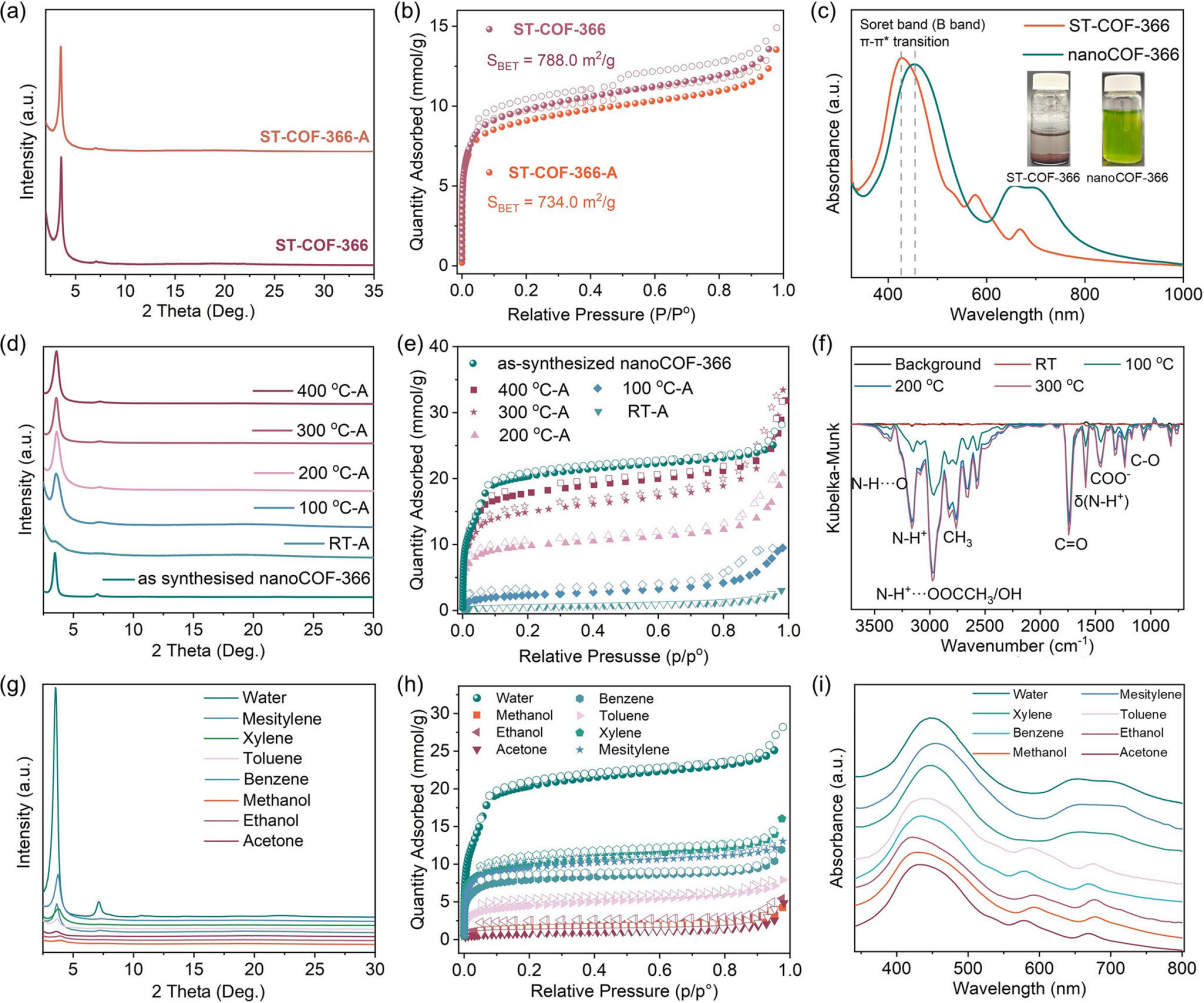
(a) PXRD patterns and (b) N_2_ sorption isotherms of as‐synthesized ST‐COF‐366 and the sample after soaking in acetone for 24 h (ST‐COF‐366‐A); (c) UV–vis spectra of aqueous suspensions of ST‐COF‐366 and nanoCOF‐366. Inset: optical images of the corresponding aqueous suspensions; (d) PXRD patterns and (e) N_2_ sorption isotherms of as‐synthesized nanoCOF‐366 and samples treated by heating at different temperatures (25°C–400°C) followed by acetone treatment; (f) in situ DRIFTS of nanoCOF‐366 during heating at different temperatures; (g) PXRD patterns and (h) N_2_ sorption isotherms of nanoCOF‐366 after treatment with different solvents; (i) UV–vis spectra of nanoCOF‐366 after treatment with various solvents; samples were redispersed in water prior to measurement.

To demonstrate the generality of solvent‐induced structural changes in protonated porphyrin‐based 2D COFs, as well as the influence of side groups on COF stability, we synthesized three additional 2D COFs using 4,4′‐biphenyldialdehyde (BD), 2,5‐dimethoxyterephthalaldehyde (DMTP), and 2,5‐dihydroxyterephthalaldehyde (DHTP) via a similar aqueous synthesis approach (Figure 4a). The resulting samples, TAPP‐BD COF, TAPP‐DMTP COF, and TAPP‐DHTP COF, exhibited high crystallinity and porosity. They also displayed similar protonated structures and nanorod morphologies, analogous to nanoCOF‐366 (Figures S23 and S24). Time‐resolved ex situ PXRD measurements of the COFs treated with acetone revealed that TAPP‐BD COF lost its crystallinity most rapidly (Figure 4b,f), which can be attributed to its larger pores that facilitate solvent intercalation between layers. In contrast, TAPP‐DMTP COF and TAPP‐DHTP COF, which contain methoxy and hydroxyl side groups, showed significantly less reduction in both crystallinity and porosity (Figures 4d–i and S25). This enhanced stability is attributed to intra‐ and interlayer hydrogen‐bonding networks introduced by the methoxy and hydroxyl groups, which strengthen interlayer interactions and prevent solvent‐induced structural disruption [6, 47]. Together, these results highlight the critical roles of porphyrin protonation state, solvent properties, and nanoCOF molecular structure in governing solvent‐induced structural evolution.

**FIGURE 4:**
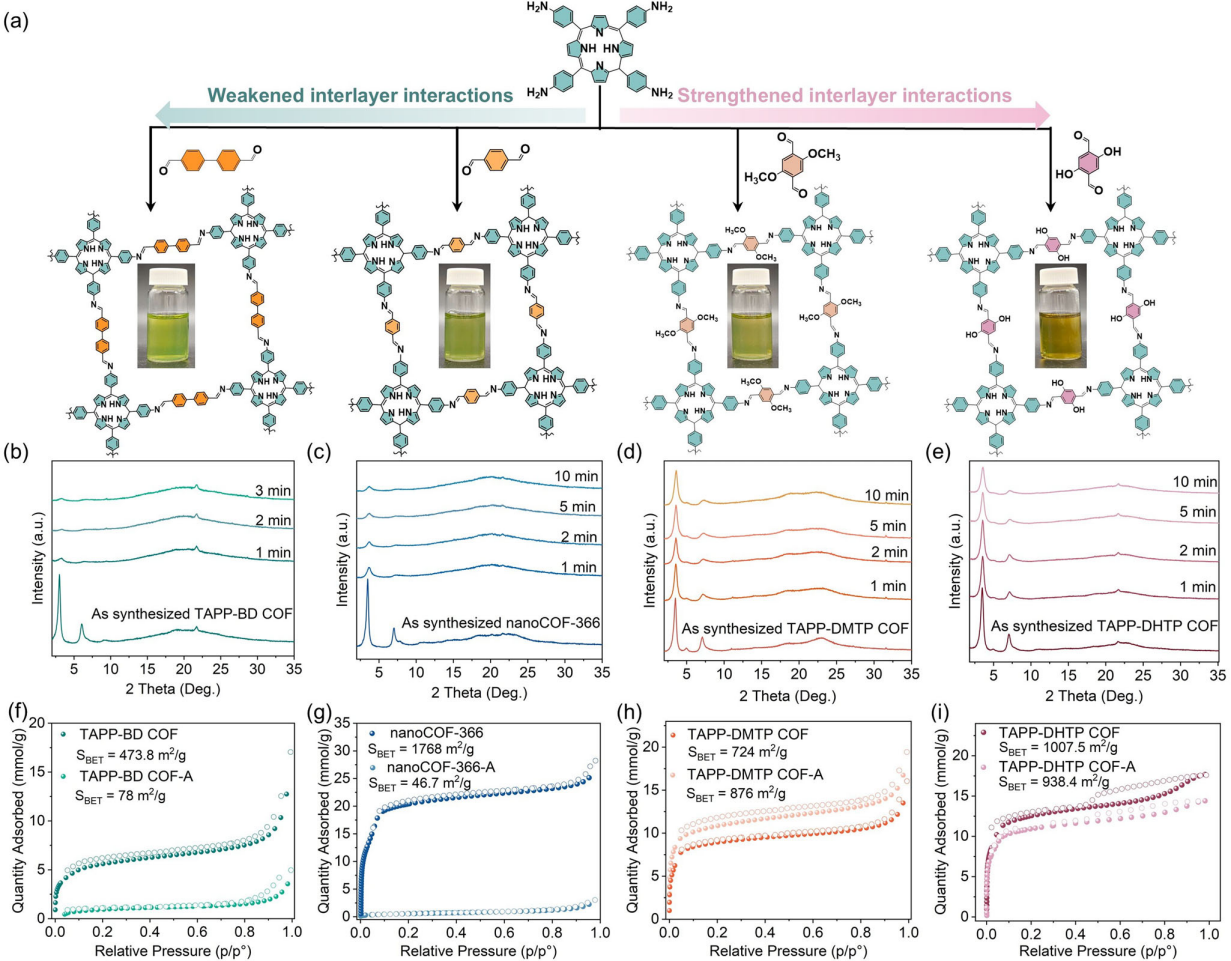
Synthesis of other TAPP‐based COFs. (a) Schematic synthesis route of porphyrin‐based COFs with linkers incorporating different side groups; (b–e) time‐dependent ex situ PXRD patterns of TAPP‐BD (b), nanoCOF‐366 (c), TAPP‐DMTP COF (d), and TAPP‐DHTP COF (e) during treatment with acetone vapor; (f–i) N_2_ adsorption–desorption isotherms of as‐synthesized and acetone‐treated TAPP‐BD COF (f), nanoCOF‐366 (g), TAPP‐DMTP COF (h), and TAPP‐DHTP COF (i).

### 2.3 Gas Sensing Performance

Porphyrin‐based COFs have been demonstrated as excellent chemiresistive NO_2_ sensors due to their highly conjugated porphyrin *π*‐systems and electron‐rich imine linkages [13, 43, 48], which facilitate strong charge‐transfer interactions with NO_2_. Given the protonation‐ and solvent‐dependent structural evolution of nanoCOF‐366, we hypothesized that tuning interlayer interactions and active‐site accessibility could further enhance its gas‐sensing performance. To test this, as‐synthesized nanoCOF‐366, acetone‐treated nanoCOF‐366‐A, and ST‐COF‐366 were deposited onto interdigital electrodes, and their chemiresistive responses were evaluated at room temperature. All materials exhibited pronounced responses to NO_2_, while negligible responses were observed toward other gases (e.g., CH_3_OH, C_3_H_6_O, C_6_H_6_, C_7_H_8_, C_2_H_6_, TEA, H_2_S, NH_3_, CO, H_2_, SO_2_, and NO), demonstrating good selectivity (Figure S26). This behavior is attributed to the comparatively weak adsorption and limited electronic interaction of these gases with the COF framework, compared with the strong electron‐withdrawing nature of NO_2_ enabling efficient charge transfer with the framework. Remarkably, nanoCOF‐366‐A exhibited an ultrahigh response of 1083.0 to 10 ppm NO_2_, more than 10 and 50 times higher than those of as‐synthesized nanoCOF‐366 (92.6) and ST‐COF‐366 (27.1), respectively (Figures 5a,b and S27–S29; Table S1), despite its reduced crystallinity and surface area. Notably, time‐dependent acetone treatment experiments demonstrate a gradual increase in NO_2_ sensing response with prolonged acetone exposure (Figure S30). This substantial enhancement is attributed to acetone‐induced disruption of interlayer packing together with porphyrin deprotonation, which increases the accessibility of electron‐rich N sites in porphyrin units and promotes stronger charge‐transfer interactions with NO_2_. In contrast, most porphyrin units are buried within the framework in as‐synthesized nanoCOF‐366 with an ordered, eclipsed‐stacked structure, rendering the porphyrin N sites largely inaccessible (Figures S31 and S32). The highly aggregated morphology of ST‐COF‐366 further restricts accessibility, resulting in even poorer sensing performance. To further elucidate the origin of this enhancement, NO_2_ adsorption in the 1–10 ppm range was quantified using microcantilever measurements, which revealed that nanoCOF‐366‐A exhibited significantly higher uptake than nanoCOF‐366 (Figures 5c and S33). This observation is supported by adsorption simulations (Figures S34 and S35), which indicate that interlayer slipping and structural disorder increase the accessibility and binding affinity of the porphyrin sites, thereby promoting stronger NO_2_ adsorption and charge transfer. Moreover, the conversion of protonated porphyrin species in nanoCOF‐366 to the neutral free‐base form upon acetone treatment restores the availability of the porphyrin nitrogen lone pairs, strengthening NO_2_ adsorption and facilitating more efficient electron transfer between NO_2_ and the COF framework. This interpretation is further supported by control experiments, where TEA‐treated nanoCOF‐366, which retains high crystallinity and porosity but undergoes deprotonation of the porphyrin sites, exhibits significantly enhanced NO_2_ sensing performance compared with as‐synthesized nanoCOF‐366 (Figure S36). It is noteworthy that nanoCOF‐366‐A exhibits a higher sensing response than the pristine TAPP linker. Although TAPP contains accessible porphyrin and amine sites, the lack of a porous architecture and extended π‐conjugated framework limits NO_2_ adsorption and charge transport (Figure S37). Collectively, these results indicate that acetone treatment enhances NO_2_ sensing through a synergistic combination of interlayer disordering and porphyrin deprotonation, which increase accessibility of porphyrin‐N sites, strengthen NO_2_ adsorption, and facilitate charge transfer between NO_2_ and the COF framework.

**FIGURE 5:**
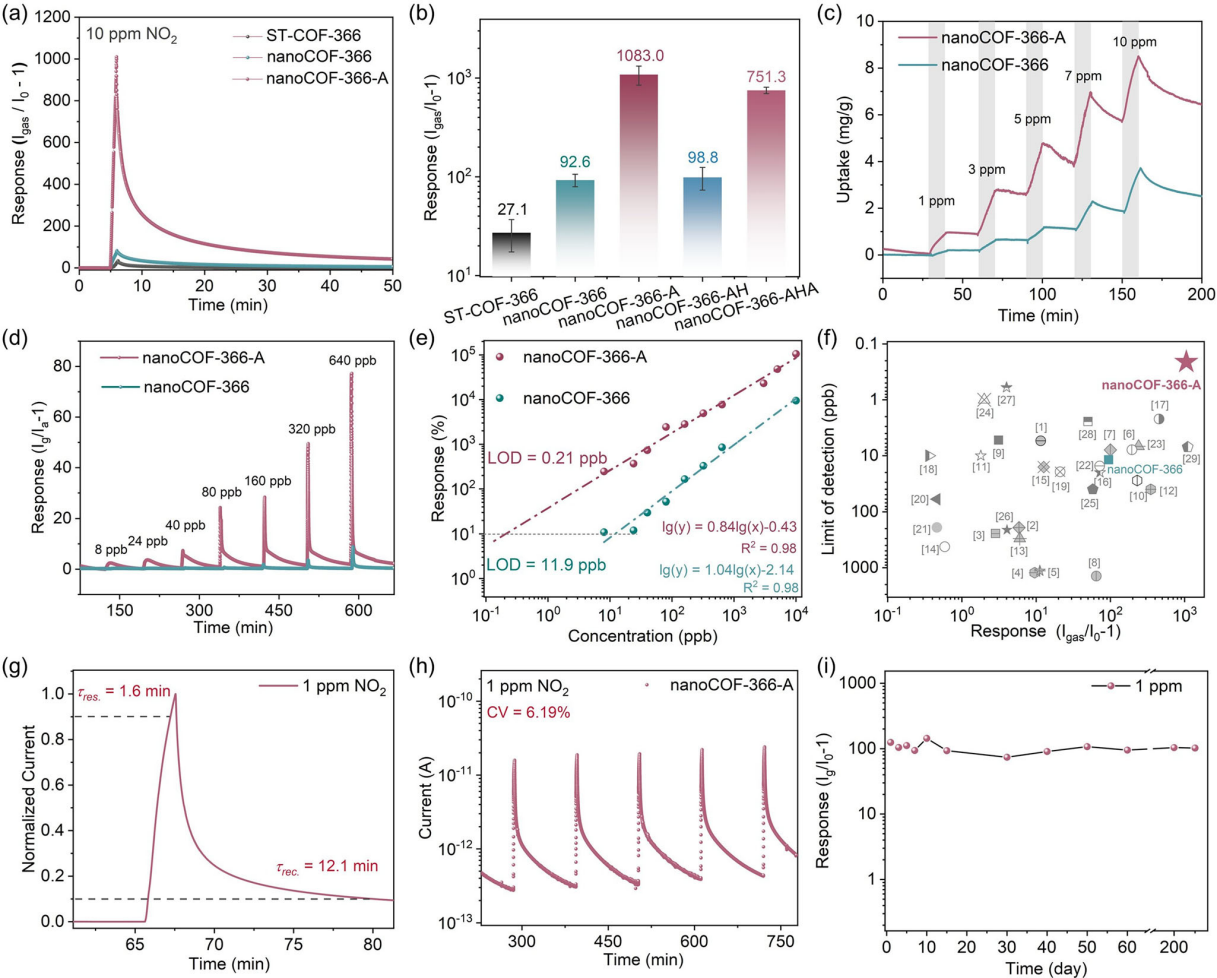
Gas‐sensing performances of the devices at room temperature. (a) Response–recovery curves to 10 ppm NO_2_ of nanoCOF‐366, nanoCOF‐366‐A, and ST‐COF‐366; (b) comparison of responses of different samples; (c) NO_2_ adsorption on nanoCOF‐366 and nanoCOF‐366‐A in the range of 1–10 ppm recorded with a microcantilever; (d) dynamic response curves of nanoCOF‐366 and nanoCOF‐366‐A toward 8–640 ppb NO_2_; (e) linear fit of log(response) versus log(NO_2_ concentration) for nanoCOF‐366 and nanoCOF‐366‐A, with calculated limits of detection (LOD); (f) comparison of NO_2_ sensing performance of nanoCOF‐366, including response to 10 ppm NO_2_ and LOD, with reported chemiresistive sensing materials (details and references are provided in Table S2, and the numbers in brackets refer to the corresponding sample numbers in Table S2). (g) Normalized response–recovery curves of nanoCOF‐366‐A to 1 ppm NO_2_; (h) response curves of nanoCOF‐366‐A over five successive cycles upon exposure to 1 ppm NO_2_; (i) long‐term stability of NO_2_ sensing performance of nanoCOF‐366‐A during aging under ambient conditions for 205 days.

Since the protonated porphyrin species and crystalline structure of nanoCOF‐366‐A can be partially restored after water soaking, likely due to re‐protonation of the porphyrin units and the re‐establishment of interlayer interactions, we further investigated its sensing behavior under alternating acetone and water treatments to establish the relationship between structural state and sensing performance. Water soaking reduced the NO_2_ response of nanoCOF‐366‐A‐H to 98.8 at 10 ppm, close to that of the as‐synthesized nanoCOF‐366 (92.6). Subsequent acetone retreatment restored the high response to 751.3 (Figures 5b, S38, and S39). Three consecutive treatment cycles exhibited the same trend, demonstrating reproducible and reversible modulation of sensing performance (Figure S40). These results demonstrate that solvent‐induced structural evolution and protonation–deprotonation of the porphyrin units are closely linked to the NO_2_ sensing behavior of nanoCOF‐366.

We next evaluated the sensing performance of nanoCOF‐366‐A in detail. NanoCOF‐366‐A exhibited pronounced responses across a wide NO_2_ concentration range (8 ppb–10 ppm), extending down to the ppb level (Figures 5d and S41). It exhibited a linear response–concentration relationship over the range of 8 ppb to 10 ppm (*R* ^2^ = 0.98), from which an ultralow LOD of 0.21 ppb was determined, in contrast to the higher LOD of approximately 11.9 ppb for nanoCOF‐366 (Figure 5e). Remarkably, to the best of our knowledge, nanoCOF‐366‐A exhibits one of the highest responses and lowest LOD reported for chemiresistive NO_2_ sensors (Figure 5f, Table S2). In addition to its high sensitivity, nanoCOF‐366‐A showed rapid and reversible sensing behavior, with a response time of 1.6 min and recovery time within 12.1 min to 1 ppm NO_2_ (Figure 5g). It also demonstrated excellent repeatability, with a coefficient of variation of 6.19% over five cycles (Figure 5h), outstanding long‐term stability, retaining 98% of its initial response after 205 days, and stable performance under different operating conditions, including high humidity and elevated temperature (Figures 5i and S42–S44). Collectively, the exceptional sensing performance of nanoCOF‐366‐A arises from synergistic structural and chemical features: 1) abundant electron‐rich imine and porphyrin nitrogen sites provide highly active centers for NO_2_ adsorption and charge transfer; 2) a partially loosened interlayer structure improves active‐site accessibility and facilitates gas diffusion; and (3) a well‐defined nanorod morphology shortens diffusion pathways and enhances charge transport. These combined features enable rapid, sensitive, and stable NO_2_ detection.

### 2.4 Mechanism Studies of NO_2_ Sensing

To elucidate the underlying sensing mechanism, time‐resolved in situ DRIFTS measurements were performed on nanoCOF‐366‐A during exposure to 100 ppm NO_2_ (Figure 6a). The time‐dependent DRIFTS spectra reveal the progressive emergence and growth of several characteristic vibrational bands, indicating strong interactions between NO_2_ and the COF framework. The most pronounced peaks at 1548 and 1383 cm^−1^ are assigned to the asymmetric and symmetric N─O stretching vibrations of surface‐bound nitrite (NO_2_^−^) and nitrate (NO_3_^−^) species, indicating chemical transformation of NO_2_ at the electron‐rich imine and porphyrin nitrogen sites of the COF framework [48, 49]. Additional bands at 1142, 1034, and 915 cm^−1^ correspond to N─O stretching and bending modes of coordinated nitro species, further supporting the formation of chemisorbed NO_2_ intermediates [50, 51]. A weak band at approximately 2255 cm^−1^ is assigned to the N–O stretching vibration of nitrosonium (NO^+^), providing direct evidence of electron transfer and surface‐mediated disproportionation of NO_2_ [52]. Broad absorption in the 2500–3000 cm^−1^ range, along with peaks at 3089 and 3266 cm^−1^, reflects perturbed N–H and aromatic C–H stretching vibrations of the framework, arising from hydrogen bonding and electronic interactions with adsorbed NO_2_. These results indicate interfacial electron transfer and redox‐like transformations during NO_2_ adsorption on nanoCOF‐366.

**FIGURE 6:**
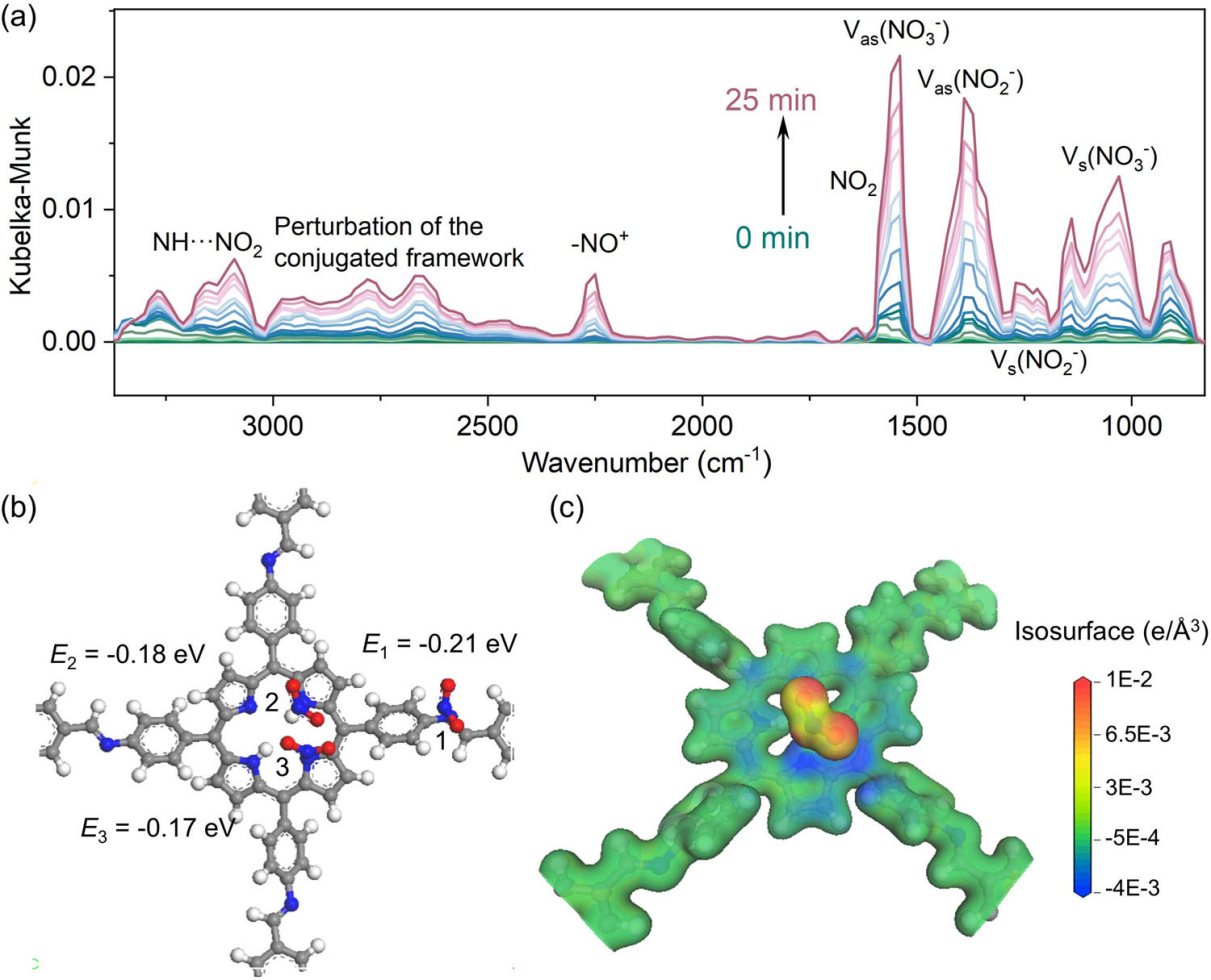
Mechanism studies. (a) Time‐resolved in situ DRIFTS difference spectra of nanoCOF‐366‐A upon exposure to 100 ppm NO_2_; (b) top views of NO_2_ adsorption configurations around the imine linkage and porphyrin moieties of COF‐366. Color coding: C, gray; H, white; N, blue; O, red; (c) electron density difference map showing charge redistribution upon adsorption of NO_2_ on COF‐366.

Building on these spectroscopic insights, density functional theory (DFT) calculations were performed to further clarify the adsorption sites and electronic interactions of NO_2_ with COF‐366. The results show that NO_2_ binds strongly at both the imine site (−0.21 eV) and the porphyrin‐N and porphyrin‐NH sites (−0.17 and −0.18 eV) (Figures 6b and S45), in agreement with DRIFTS observations indicating that NO_2_ preferentially adsorbs at nitrogen sites within the COF framework [48]. Electron density difference analysis reveals significant electron accumulation on NO_2_ and corresponding electron depletion across the porphyrin macrocycle and extended COF backbone (Figures 6c and S46), indicating electron transfer from the COF to NO_2_. These results indicate that the imine and porphyrin nitrogen sites act as electron donors, while NO_2_ serves as an electron acceptor, consistent with the formation of NO_2_‐derived intermediates inferred from the DRIFTS spectra. This electron transfer modulates the charge carrier concentration within the p‐type COF network, increasing hole density and thereby enhancing electrical conductivity. As a result, an increased current is observed upon NO_2_ exposure during the chemiresistive sensing measurements. Together, the DRIFTS and DFT results demonstrate strong adsorption and electron transfer between NO_2_ and the imine and porphyrin nitrogen sites in COF‐366, which modulate the electronic structure of the conjugated framework and govern its high sensitivity toward NO_2_.

## 3 Conclusion

This study demonstrates the ambient aqueous synthesis of porphyrin‐based nanoCOFs and presents an effective strategy for regulating their structure through simple post‐treatment with organic solvents. The as‐synthesized samples, containing protonated porphyrin units with well‐defined nanoscale dimensions and good dispersibility, facilitate the insertion of solvent molecules between COF layers. As a result, treatment with organic solvents induces deprotonation of the porphyrin units, weakens interlayer interactions, and disrupts the ordered interlayer stacking, leading to a more disordered structure. A series of comparative experiments demonstrate that the protonated structure and solvent properties play a crucial role in the structural modulation. Despite a significant reduction in crystallinity and surface area upon solvent treatment, the resulting structural changes expose porphyrin units that are buried within the more crystalline as‐synthesized material. Meanwhile, deprotonation restores the electron‐rich character of the porphyrin centers, enhancing their interaction with guest molecules and facilitating charge transfer. Owing to the synergistic effects, the solvent‐treated nanoCOF‐366 exhibit 10‐fold higher chemiresistive NO_2_ sensing performance than the as‐synthesized sample. These results demonstrate that aqueous synthesis not only offers a green and sustainable route but also provides an additional handle for structural and electronic regulation of COFs. We anticipate that this approach can be further developed to enable facile, scalable, and environmentally friendly COF synthesis while producing materials with properties distinct from those obtained via conventional methods, thereby enriching the toolbox for structure–property engineering and enhancing COF performance across various applications.

## Author Contributions

**Yu Pan**: conceptualization, methodology, validation, investigation, data curation, formal analysis, visualization, writing – original draft. **Xueying Kong**: conceptualization, methodology, formal analysis, data curation, validation, investigation, visualization. **Guangling Liang**: investigation, validation. **Samson Afewerki**: investigation, methodology, validation, data curation, formal analysis. **Chao Xu**: conceptualization, methodology, formal analysis, supervision, resources, project administration, funding acquisition, writing – original draft, writing – review and editing.

## Conflicts of Interest

The authors declare no conflicts of interest.

## Supporting information




**Supporting File 1**: anie74119‐sup‐0001‐SuppMat.pdf.

## Data Availability

The data that support the findings of this study are available from the corresponding author upon reasonable request.
