# Supplementary material for: Solvent‐Induced Structural Modulation in Nanoscale Covalent Organic Frameworks Enables High‐Performance NO2 Sensing
Source: Angew Chem Int Ed Engl. 2026 Aug 13;65(41):e2684732. doi: 10.1002/anie.2684732 (PMC13638090; doi:10.1002/anie.2684732)
Supplement: Supplementary file 1 [file ANIE-65-e2684732-s001.pdf]

Supporting information

Solvent-Induced Structural Modulation in Nanoscale Covalent Organic Frameworks Enables High-Performance NO_2 Sensing

Yu Pan[†‡], *Xueying Kong*[‡]*, *Guangling Liang*[†], *Samson Afewerki*[‡§], *Chao Xu*[‡]*

[†] State Key Laboratory of Structural Chemistry, Fujian Institute of Research on the Structure of Matter, Chinese Academy of Sciences (CAS), Fuzhou 350002, China; University of Chinese Academy of Sciences (UCAS), Beijing 100049, PR China

[‡] Division of Nanotechnology and Functional Materials, Department of Materials Science and Engineering, Uppsala University, Uppsala, SE-75121, Sweden.

[§] Department of Chemistry, United Arab Emirates University, Box 15551, Al Ain, UAE

1. Materials and Syntheses

1.1 Materials

All chemicals and solvents were of analytical grade and used without further purification unless otherwise specified. 5,10,15,20-Tetrakis(4-aminophenyl)porphyrin (TAPP), terephthalaldehyde (TPA), 2,5-dimethoxyterephthalaldehyde (DMTP), 2,5-2,5-dihydroxyterephthalaldehyde (DHTP), and 4,4′-biphenyldicarboxaldehyde (BD) were purchased from Aladdin and used as received. Acetic acid (HOAc, 99.7%) was used without further purification.

1.2 Syntheses

Synthesis of COF-366

Both an aqueous approach and a solvothermal approach were employed for the synthesis of COF-366. The sample synthesized via the aqueous method was denoted as nanoCOF-366, while the sample prepared using the solvothermal method was denoted as ST-COF-366.

For the synthesis of nanoCOF-366, TPA (0.20 mmol) was added to 1 mL of glacial acetic acid, and the mixture was stirred for 30 min to form a clear solution. Meanwhile, TAPP (0.10 mmol) was dispersed in 1 mL of deionized water and stirred for 30 min to obtain a homogeneous suspension. Subsequently, the TPA solution was added dropwise to the TAPP suspension, and the mixture immediately turned dark brown. The reaction mixture was allowed to stand at room temperature for 72 h without stirring. The resulting dark precipitate was collected by filtration using a PVDF membrane and thoroughly washed with deionized water. The product was either redispersed in water to form a homogeneous suspension or dried in a vacuum oven to obtain the solid material.

Note that the purification procedure significantly influences the surface area of the final product. We found that washing the sample by filtration is more effective than repeated centrifugation. The filtration method yielded a much higher BET surface area of 1768 m^2/g, compared to 1500 m^2/g for the sample washed by repeated centrifugation.

The synthesis of ST-COF-366 followed the protocol reported in the literature.[1] Briefly, TAPP (0.02 mmol) and TPA (0.04 mmol) were charged into a Pyrex tube, followed by the addition of a solvent mixture consisting of 0.5 mL ethanol and 0.5 mL mesitylene, with 0.1 mL of 6 M aqueous acetic acid as the catalyst. The reaction mixture was subjected to three freeze–pump–thaw cycles and then sealed under vacuum. The tube was heated at 120 °C for 3 days. The resulting precipitate was collected and purified by Soxhlet extraction with acetone for 24 h, followed by drying under vacuum at 80 °C.

Synthesis of Functionalized Porphyrin COFs

Porphyrin-based COFs incorporating different dialdehyde linkers were synthesized in aqueous acetic acid solution using the same method as for nanoCOF-366, where BD, DMTP, and DHTP were used to replace TPA, respectively. These syntheses resulted in TAPP-BD COF, TAPP-DMTP COF, and TAPP-DHTP COF.

Synthesis of nanoCOF-366-A and nanoCOF-366-AH

For the solvent-induced structural transformation, 10 mg of as-synthesized nanoCOF-366 was dispersed in 10 mL of acetone and allowed to stand for 24 h. The solid was then collected and dried under vacuum to obtain nanoCOF-366-A. For the recovery experiments, the solvent-treated sample (nanoCOF-366-A) collected from acetone by centrifugation (without drying) was redispersed in 10 mL of deionized water and allowed to stand for 48 h. The resulting product was then collected, washed with water, and dried, yielding nanoCOF-366-AH.

Note that it is very important to keep the acetone-treated sample in a wet state and avoid drying or fully evaporating the residual solvent prior to water treatment for recovery. Once the acetone-treated sample is fully dried, the recovery of crystallinity in water becomes less efficient.

Characterization

The morphology of the samples was examined using scanning electron microscopy (SEM, Zeiss LeoGemini 1530) and transmission electron microscopy (TEM, JEM-F200). N_2 adsorption–desorption isotherms were measured at 77 K using a Micromeritics ASAP 2020 analyzer, and pore size distributions were calculated from the adsorption branches using density functional theory (DFT) models. Prior to sorption measurements, all samples were degassed at 30 °C under high vacuum (<10^{-5} mmHg) for 6 h. Powder X-ray diffraction (PXRD) patterns were recorded on a Bruker D8 Focus diffractometer with Cu Kα radiation (λ = 1.5418 Å). Zeta potential measurements were performed using a Zetasizer (Malvern Instruments). UV–Vis absorption spectra of

solutions were collected on an Agilent Cary 60 spectrophotometer, while solid-state UV–Vis spectra were measured using a Shimadzu UV-2600 spectrophotometer. Solid-state ^{13}C NMR spectra were acquired on a Bruker Avance 400 MHz spectrometer. Diffuse reflectance infrared Fourier transform spectroscopy (DRIFTS) spectra were recorded on a Nicolet 6700 FT-IR spectrometer equipped with an MCT/A detector cooled with liquid nitrogen. Thermogravimetric analysis (TGA) was performed on a Mettler Toledo SDTA851e analyzer under a nitrogen or air flow of 60 mL/min.

2. Gas sensing measurements

The gas sensing performance of the COF samples was evaluated using a chemiresistive device configuration.

Device fabrication:

nanoCOF-366 based devices: After thoroughly washing the as-synthesized nanoCOF-366 with water several times, the material was dispersed in a small amount of aqueous solution to obtain a concentrated dispersion. Subsequently, 0.1 mL of this dispersion was drop-cast onto Ag–Pt interdigitated electrodes and dried under a gentle flow of dry air to form the sensing layer.

nanoCOF-366-A based devices: The nanoCOF-366-A device was obtained by slowly introducing a drop of acetone onto the nanoCOF-366 device, enabling solvent infiltration into the active material and triggering a structural change.

Performance testing:

A constant bias voltage of 5 V was applied to the device, and the current was monitored in real time using a source meter (Keithley 2602B or Tonghui 2690). Prior to each measurement, the chamber was purged with dry synthetic air (21% O_2 / 79% N_2) to establish a stable current baseline. 1-10 ppm NO_2 is obtained by controlling the flow rate of dry synthetic air and the purchased 100 ppm NO_2 standard gas using mass flow controllers (MFCs). Lower concentrations of NO_2 are obtained by controlling the flow

rate ratio of the carrier gas and the 4 ppm NO_2 standard gas using MFCs. The flow rates of both the synthetic air and analyte gases (diluted in air) were precisely controlled at 600 sccm using MFCs.

The sensor response (R) was defined as:

$R = (I_{gas}-I_0)/I_0$

where I_{gas} is the current in the presence of target gas and I_0 is the baseline current in air. The response and recovery times were defined as the times required for the sensor to reach 90% of the total current change upon gas exposure and removal, respectively.

In this study, the limit of detection (LOD) is defined as the NO_2 concentration that produces a 10% sensor response. Specifically, the LOD was estimated from the log–log calibration curve of response versus NO_2 concentration, which followed a power-law relationship (Response = aC^b). A linear fit was performed on the log(Response) versus log(Concentration) data to determine the constants a and b. Using the fitted equation, the NO_2 concentration corresponding to a 10% response was calculated and taken as the LOD.

4. The mass–transduced microcantilever

A commercial microcantilever sensor (RMC-100, High-End MEMS Technology Co., Ltd., China) was used to evaluate the NO_2 adsorption performance of nanoCOF-366 and nanoCOF-366-A. Adsorption of gas molecules onto the microcantilever surface induces an increase in mass, resulting in a proportional shift in the resonance frequency of the microcantilever. The measured frequency shift can be converted to the adsorbed mass using a calibration factor of 0.24 pg Hz^{-1}.

NO_2 concentrations ranging from 1 to 10 ppm were generated by mixing dry synthetic air with a commercially supplied 100 ppm NO_2 standard gas using mass flow controllers (MFCs). The flow rates of both the synthetic air and the diluted NO_2 gas were precisely maintained at 200 sccm throughout the measurements.

5. Computational methods

5.1 DFT calculations

All first-principles calculations were performed using density functional theory (DFT) with the plane-wave pseudopotential method as implemented in the CASTEP module[2] of *Materials Studio 2020*. The exchange–correlation interactions were treated with the Perdew–Burke–Ernzerhof (PBE) functional within the generalized gradient approximation (GGA)[3]. A plane-wave kinetic energy cutoff of 500 eV was used. Due to the large supercell size of the COF models, the Brillouin zone was sampled using a 1×1×1 Gamma-centered grid k-points. A vacuum layer of 20 Å was applied along the c-axis to eliminate spurious interactions between periodic images. Structural optimizations were carried out until the total energy and forces converged to 2.0×10^{-5} eV and 0.05 eV $Å^{-1}$, respectively. Tkatchenko–Scheffler (TS) dispersion corrections were applied throughout both structural optimizations and total energy calculations.

The binding energy (E_{bind}) of NO_2 on COF-366 was calculated according to the following equation:

$$E_{\text{bind}} = E_{\text{total}} - E_{\text{COF}} - E_{NO_2}$$

where E_{total}, E_{COF}, and E_{NO2} represent the total energies of the NO_2-adsorbed COF-366 system, the isolated COF-366 model, and the isolated NO_2 molecule, respectively. All calculations were performed using identical computational settings.

5.2 Sorption simulations

Gas adsorption isotherms of different COF stacking configurations were calculated using grand canonical Monte Carlo (GCMC)[4] simulations implemented in the Sorption module of *Materials Studio 2020*. A periodic supercell (2×2×8) was constructed from the optimized AA-stacked unit cell and used as a common simulation box for all models to ensure identical cell dimensions and eliminate finite-size effects. Slipped stacking configurations were generated by applying rigid in-plane random translations to

individual COF layers within the supercell while preserving the intralayer geometry. After translation, all atoms were wrapped back into the simulation cell under periodic boundary conditions. Multiple independent slipped configurations were generated to represent structural disorder.

The COMPAS III force field was employed to describe both framework and guest molecules, with the framework treated as rigid during simulations[5]. Atomic partial charges of the COF framework were obtained using the charge equilibration (QEq) method[6] implemented in Materials Studio, while those of NO_2 molecules were derived from DFT calculations using Mulliken population analysis[7] in DMol3[8]. van der Waals interactions were modeled using Lennard–Jones potentials with a cutoff distance of 12.5 Å, and long-range electrostatic interactions were evaluated using the Ewald summation method[9]. Adsorption isotherms were simulated at 298 K over a pressure range of 10^{-6}–101 kPa. Each pressure point consisted of 1,000,000 Monte Carlo equilibration steps followed by 1,000,000 production steps.

6. Additional Figures, Tables, and discussions.

Additional discussions on the chracerization of nanoCOF-366: After the purification procedure, the as synthesized nanoCOF-366 can be readily dispersed in water to form a transparent yellow–green solution, owing to its protonated structure and nanoscale morphology (Figure 1b and 1c inset). The red-shifted Soret and Q bands, together with the enhanced Q-band absorption in the UV–vis spectra of nanoCOF-366 compared to the TAPP linker, confirm the protonation of the porphyrin rings in nanoCOF-366 (Figure 1c). Scanning electron microscopy (SEM) image reveals a nanorod morphology with good uniformity, featuring average dimensions of approximately 50 × 180 nm (Figure 1b and S1a-b). This behavior is in sharp contrast to the severely aggregated particles and poor aqueous dispersibility observed for the solvothermally synthesized sample (hereafter, the aqueous-synthesized sample is denoted as nanoCOF-366, and the solvothermally synthesized sample as ST-COF-366) (Figure S1c). Powder X-ray diffraction (PXRD) analysis of the dried nanoCOF-366 showed intense reflections at 3.45, 4.85, 6.98 and 10.48°, which can be indexed to the (100), (110), (200) and (300) diffraction planes of COF-366, respectively (Figure 1d). These peaks are in good agreement with both the reported experimental data and the simulated diffraction pattern based on an eclipsed stacking model. High-resolution transmission electron microscopy (HRTEM) images reveal a well-defined grid-like framework viewed along the [001] direction, exhibiting a periodicity of approximately 2.5 nm corresponding to the d(100) spacing of COF-366 (Figure 1d inset and Figure S2). The observed pore size of ~2 nm is consistent with the crystallographic structure. The molecular structure of nanoCOFs-366 was studied by infrared (IR) and solid-state ^{13}C NMR spectroscopies. The sample exhibits a characteristic band at 1620 cm^{-1} in the IR spectra, assigned to C=N stretching vibrations, along with a resonance at ~160 ppm in the solid-state ^{13}C NMR spectra, confirming the formation of a polyimine network (Figure 1e and 1f). Nitrogen sorption measurements further confirm the presence of a rich microporous structure in nanoCOF-366, displaying a typical type-I adsorption isotherm (Figure 1g).

The material exhibits a high specific surface area of 1768 $m^2\ g^{-1}$ and a micropore size distribution centered at 1.5−2 nm, in good agreement with the pore dimensions observed by TEM and those predicted from the crystallographic model.

To assess reproducibility, we synthesized nanoCOF-366 in three independent batches and measured their N_2 sorption isotherms. The corresponding BET surface areas were 1768, 1575, and 1591 $m^2\ g^{-1}$ (Figure S3), respectively, with a relative standard deviation (RSD) of 5.32%, demonstrating good reproducibility of both the synthesis and the resulting porosity characteristics.

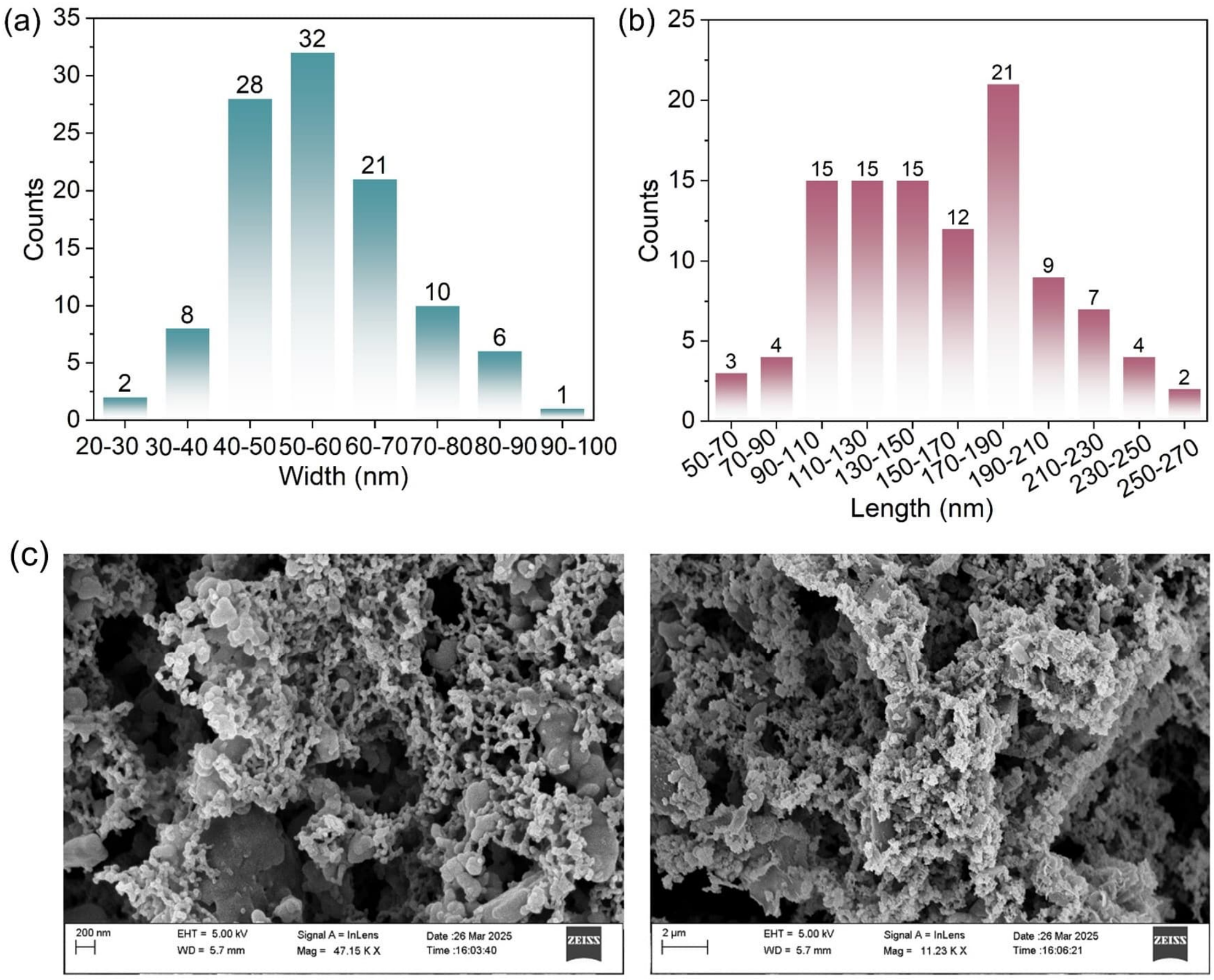

Figure S1. (a-b) Size distribution of nanoCOF-366 analyzed form SEM images, (c) SEM images of ST-COF-366 at different magnifications.

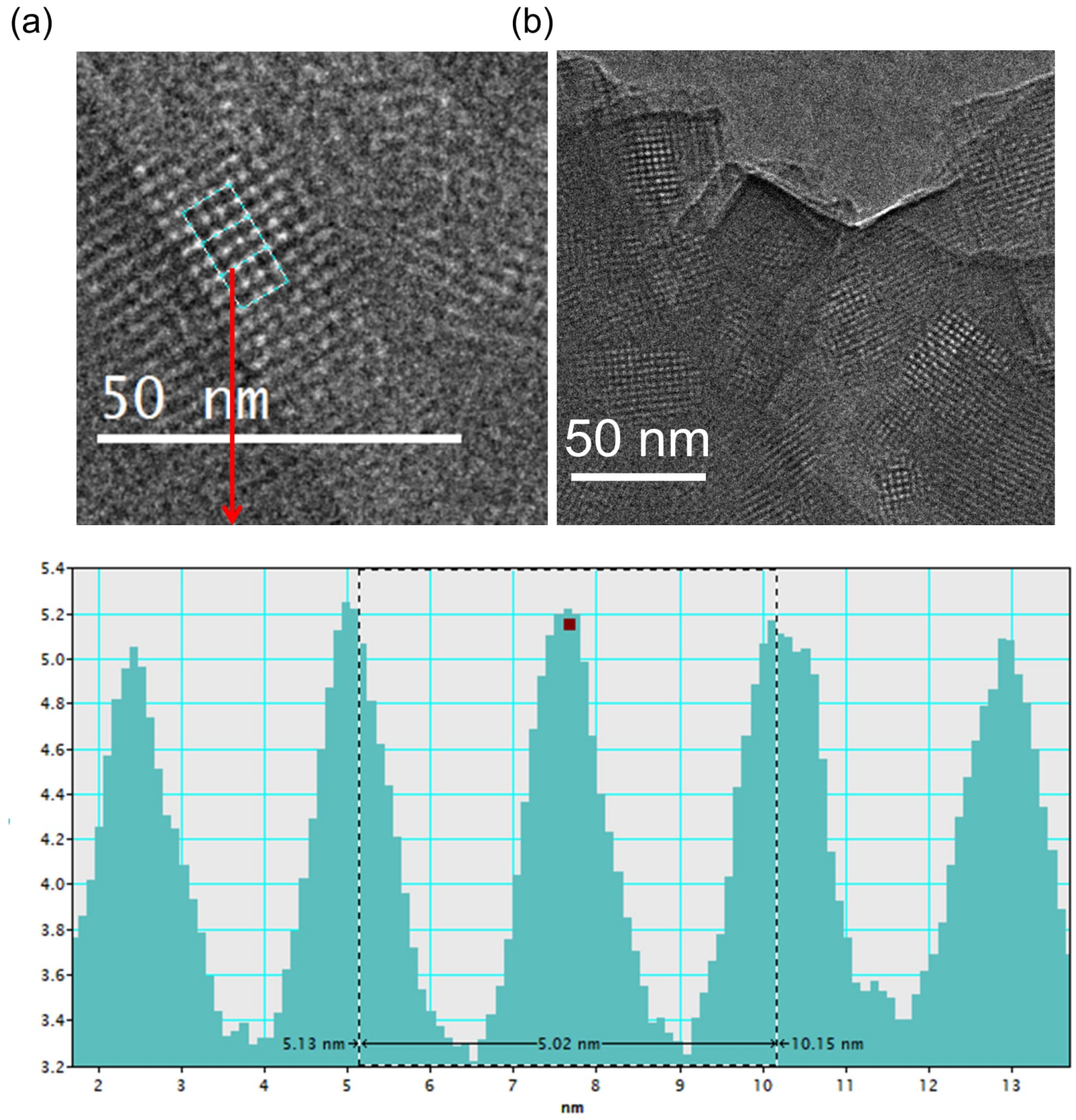

Figure S2. HRTEM images of nanoCOF-366.

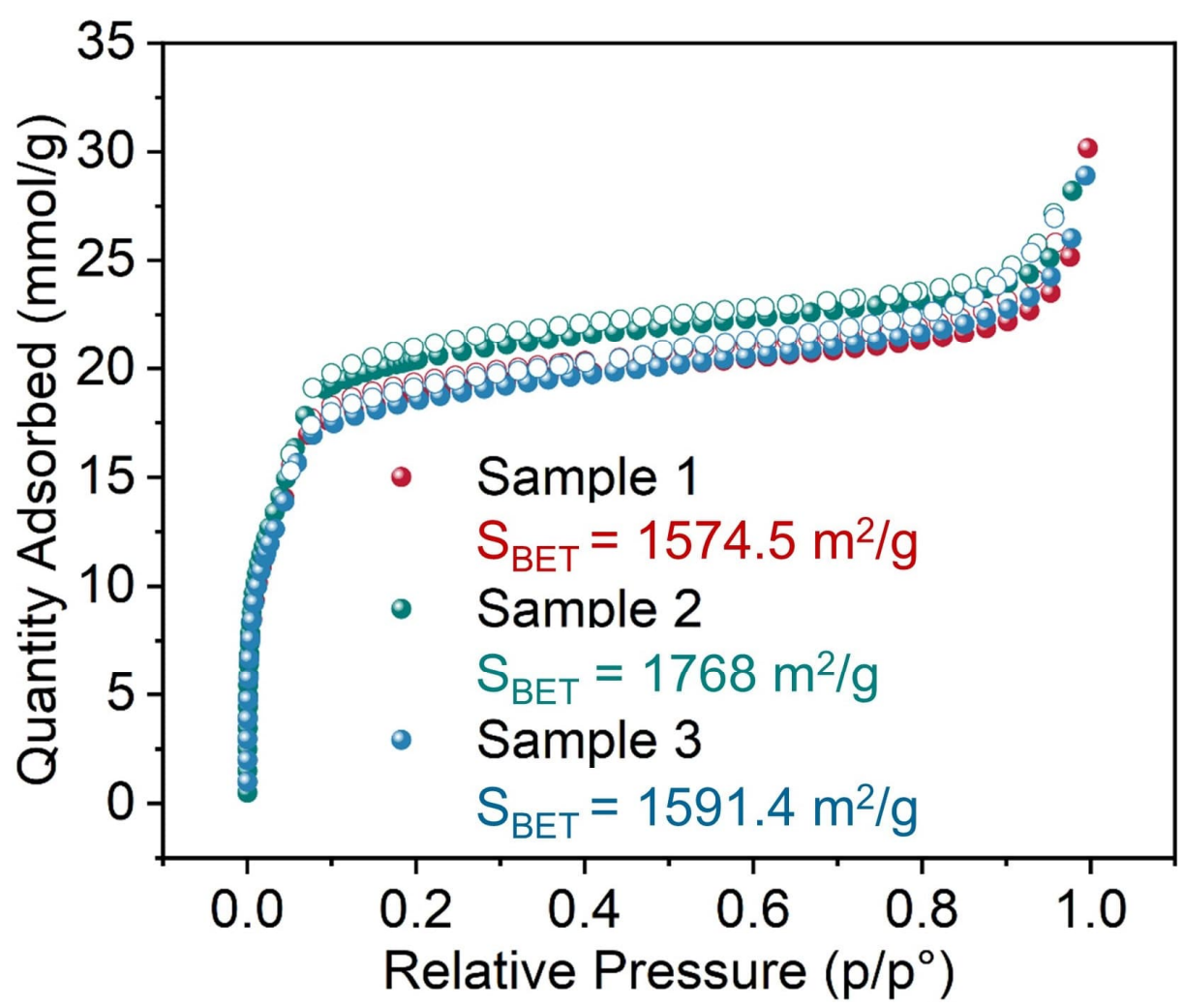

Figure S3. N_2 sorption isotherms of nanoCOF-366 synthesized in three independent batches. The corresponding BET surface areas are 1768, 1575, and 1591 $m^2\ g^{-1}$, with a relative standard deviation (RSD) of 5.32%, demonstrating good reproducibility.

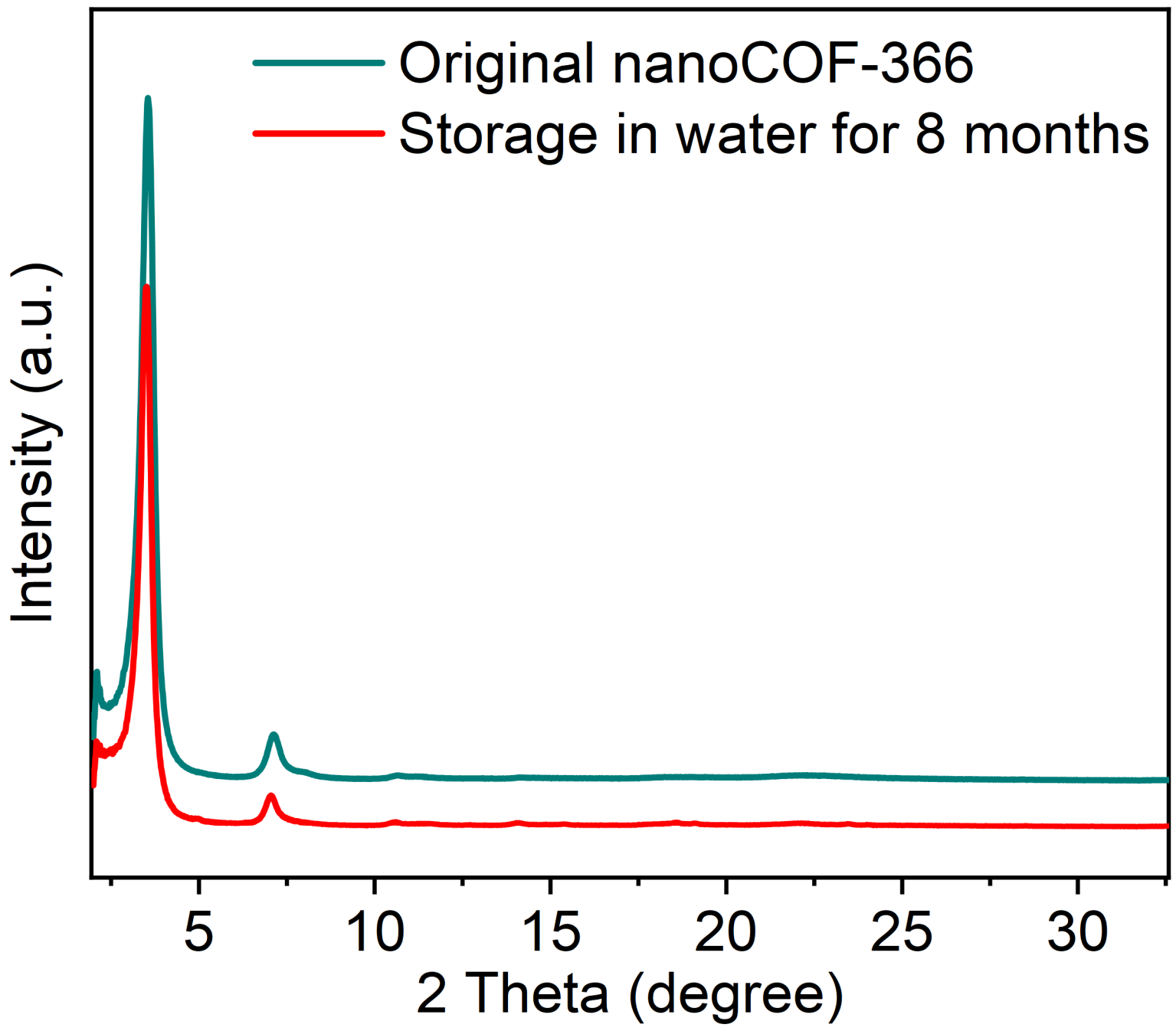

Figure S4. PXRD patterns of as-synthesized nanoCOF-366 and the sample after storage in water for 8 months.

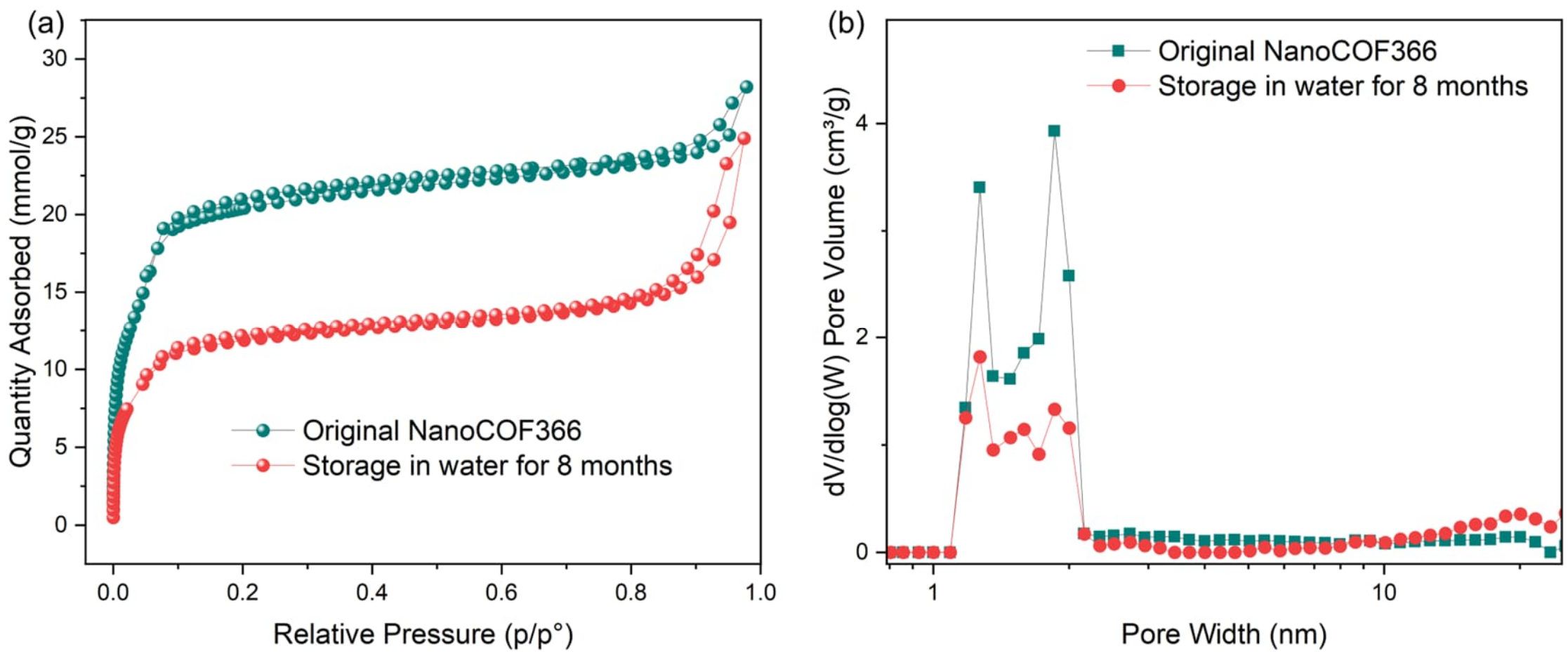

Figure S5. (a) N_2 absorption-desorption isotherms and (b) pore size distribution of as-synthesized nanoCOF-366 and the sample after storage in water for 8 months.

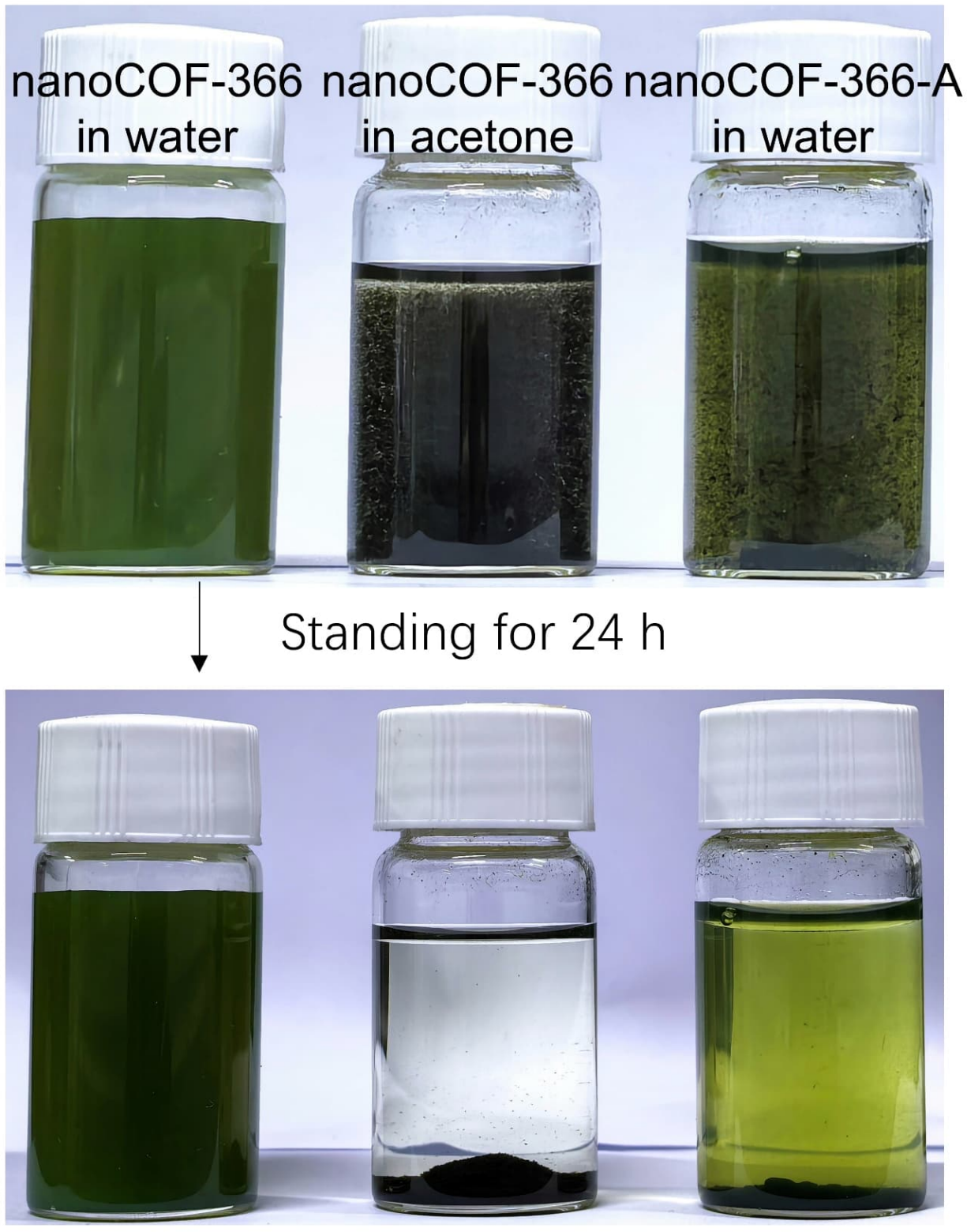

Figure S6. Optical images of as-prepared suspensions of nanoCOF-366 in water and acetone, and nanoCOF-366-A in water, along with the corresponding suspensions after standing for 24 h without stirring. The images show that the aqueous suspension of nanoCOF-366 exhibits high colloidal stability, with no significant precipitation after 24 h, whereas significant precipitation occurs in the acetone suspension of nanoCOF-366 and the aqueous suspension of nanoCOF-366-A, indicating poor colloidal stability.

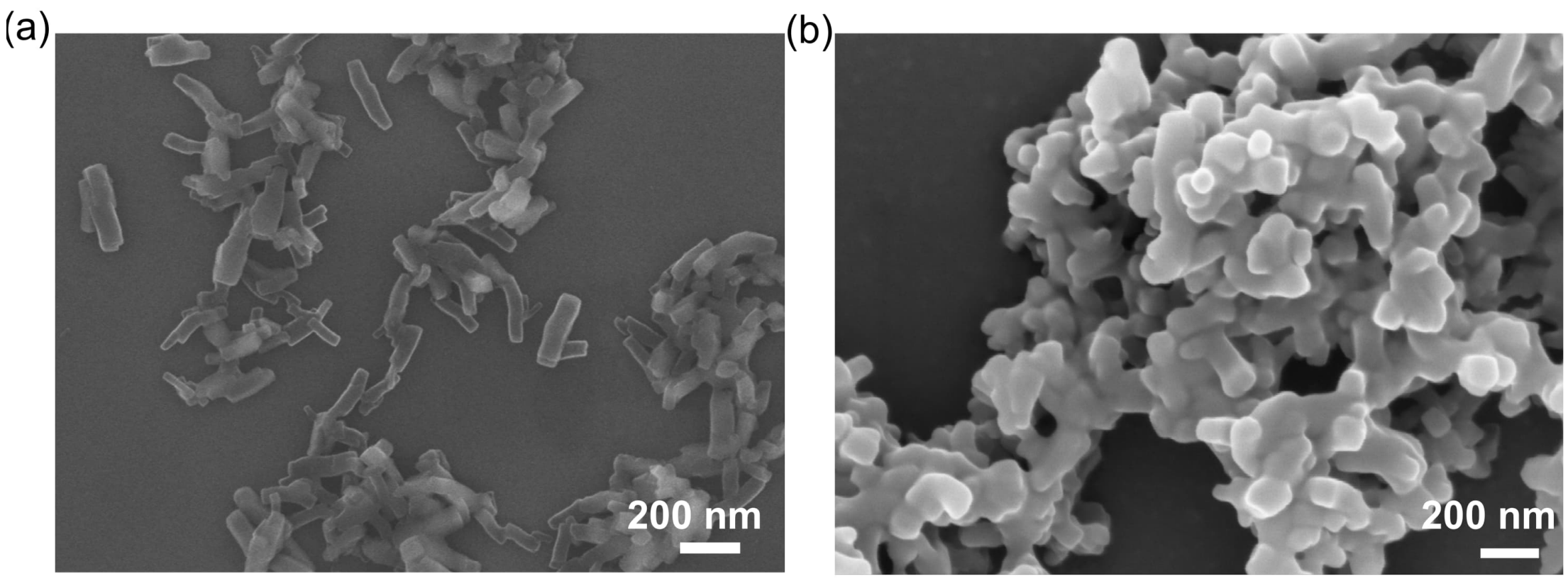

Figure S7. SEM of nanoCOF-366 and nanoCOF-366-A.

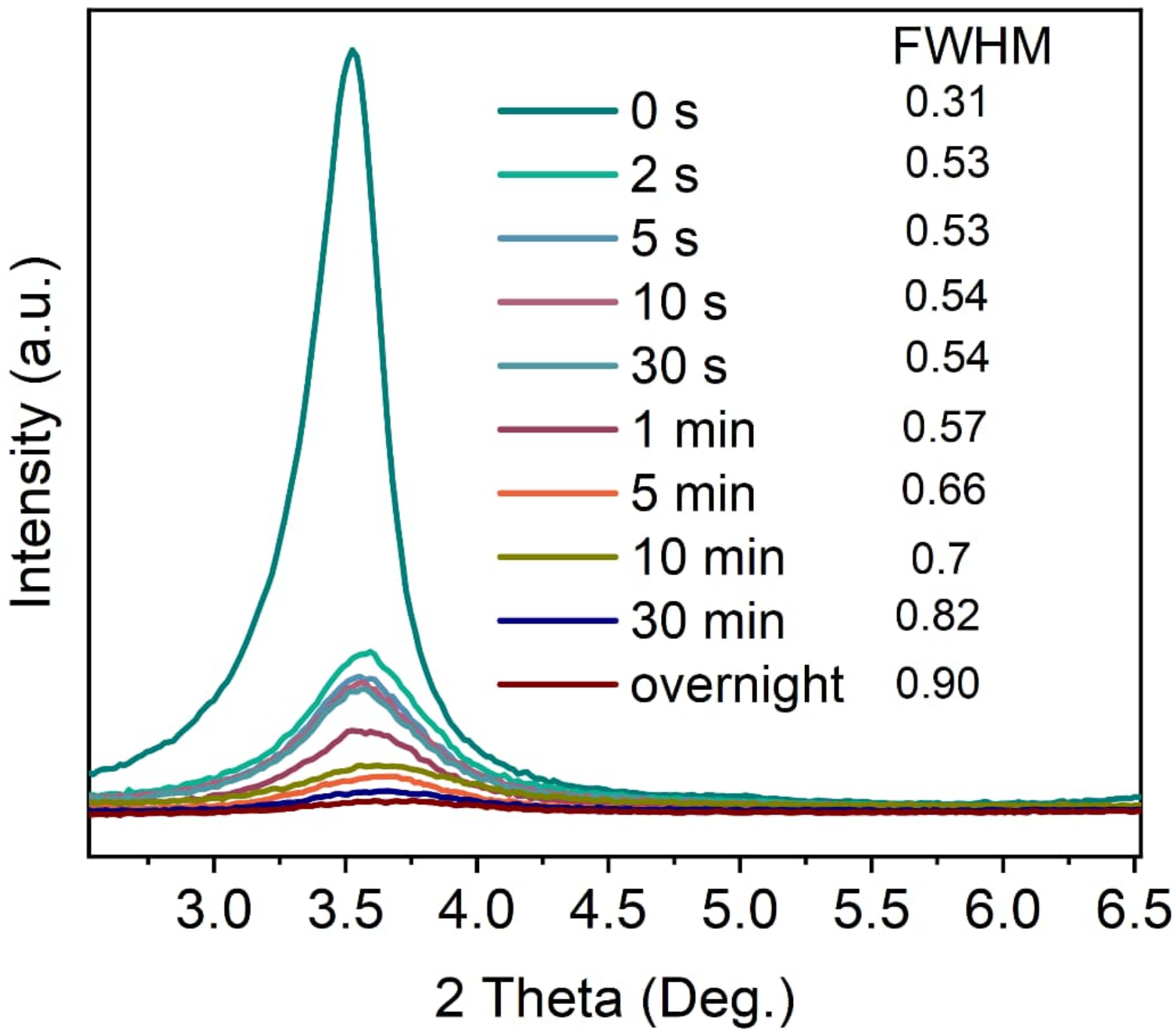

Figure S8. Time-dependent FWHM of (100) of as-synthesized nanoCOF-366 treated with acetone vapor.

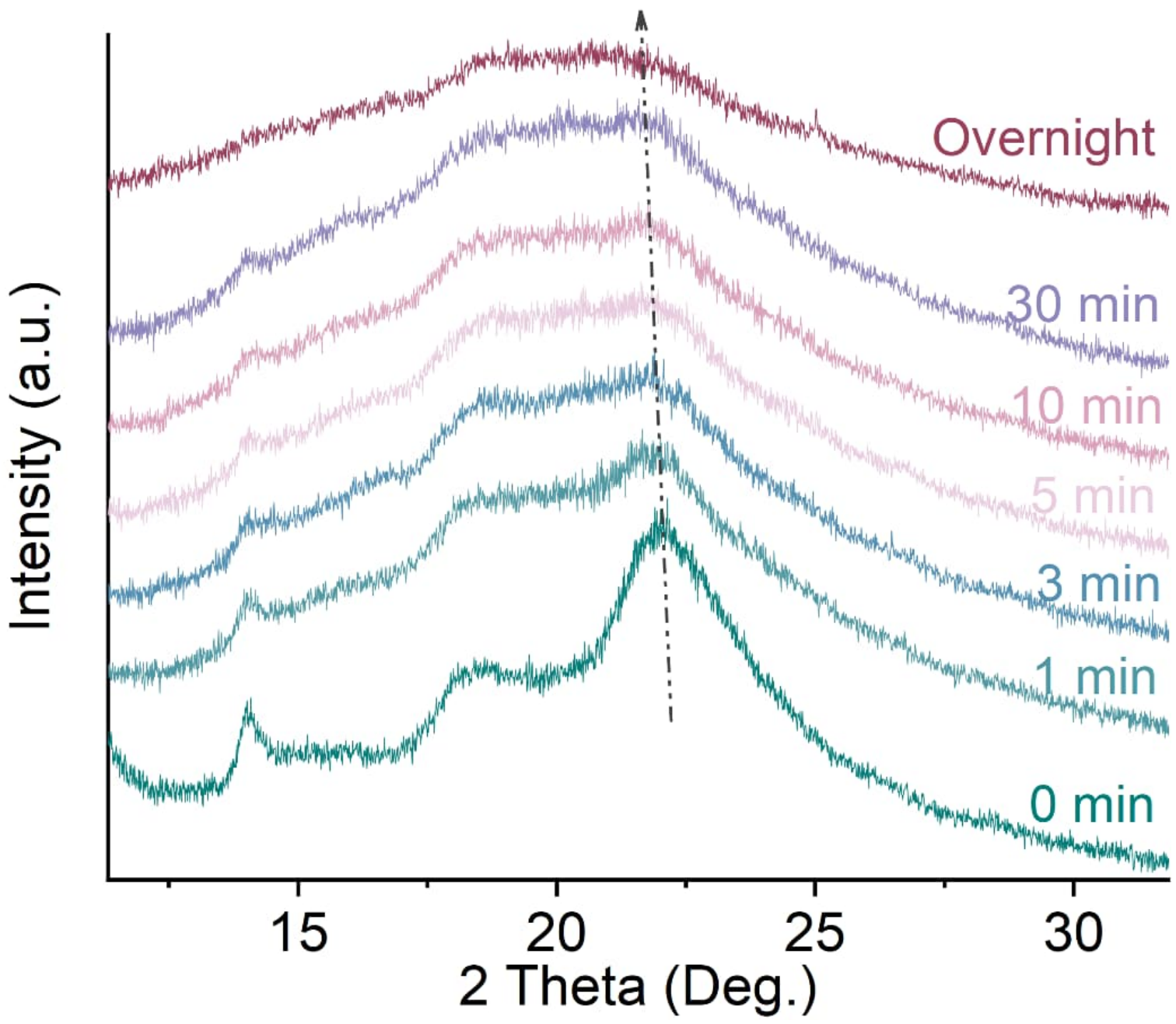

Figure S9. Time-dependent PXRD patterns of as-synthesized nanoCOF-366 during acetone vapor treatment, showing the magnified diffraction patterns in the 2θ range of 11–32°.

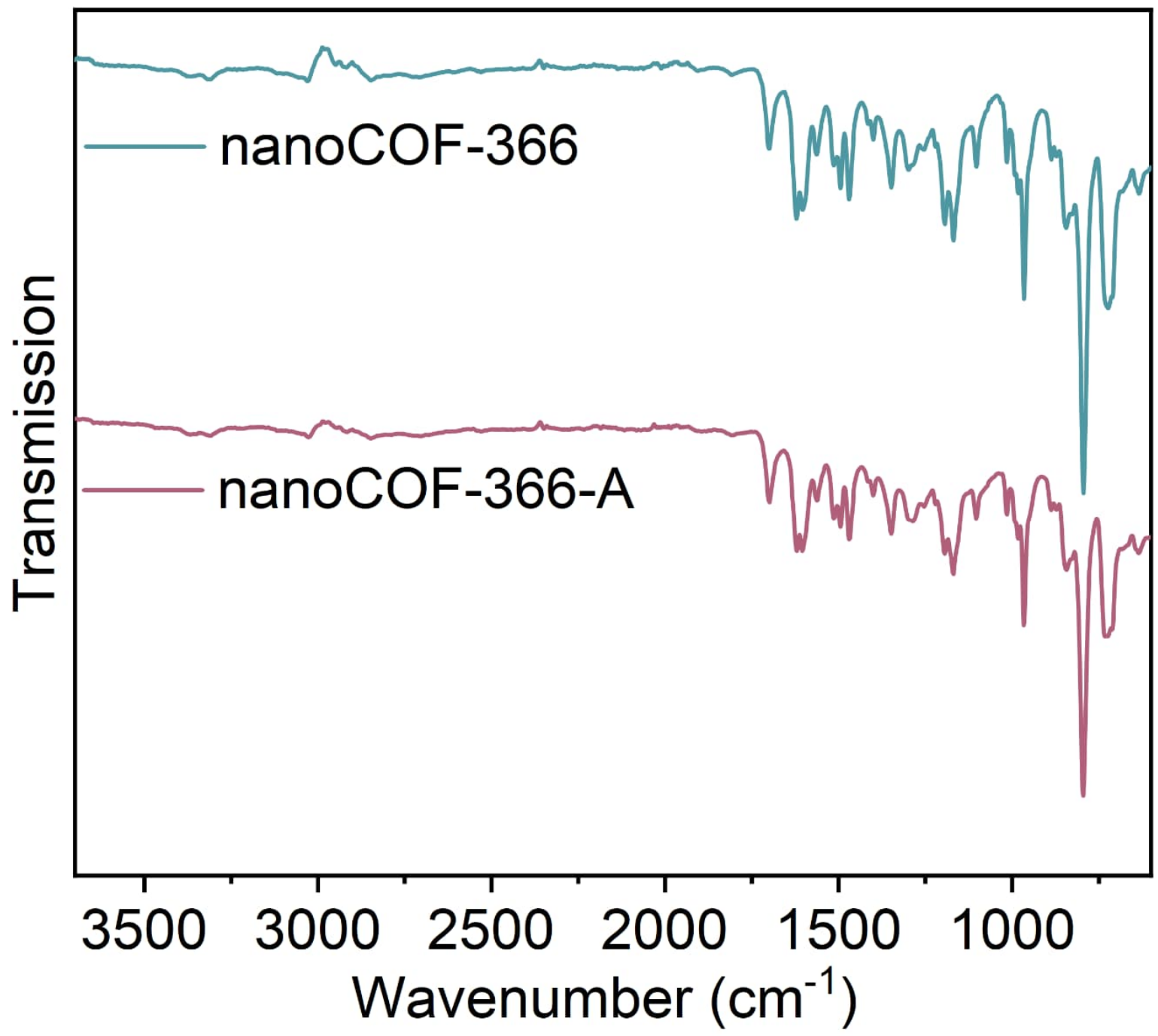

Figure S10. FT-IR spectra of nanoCOF-366 and nanoCOF-366-A.

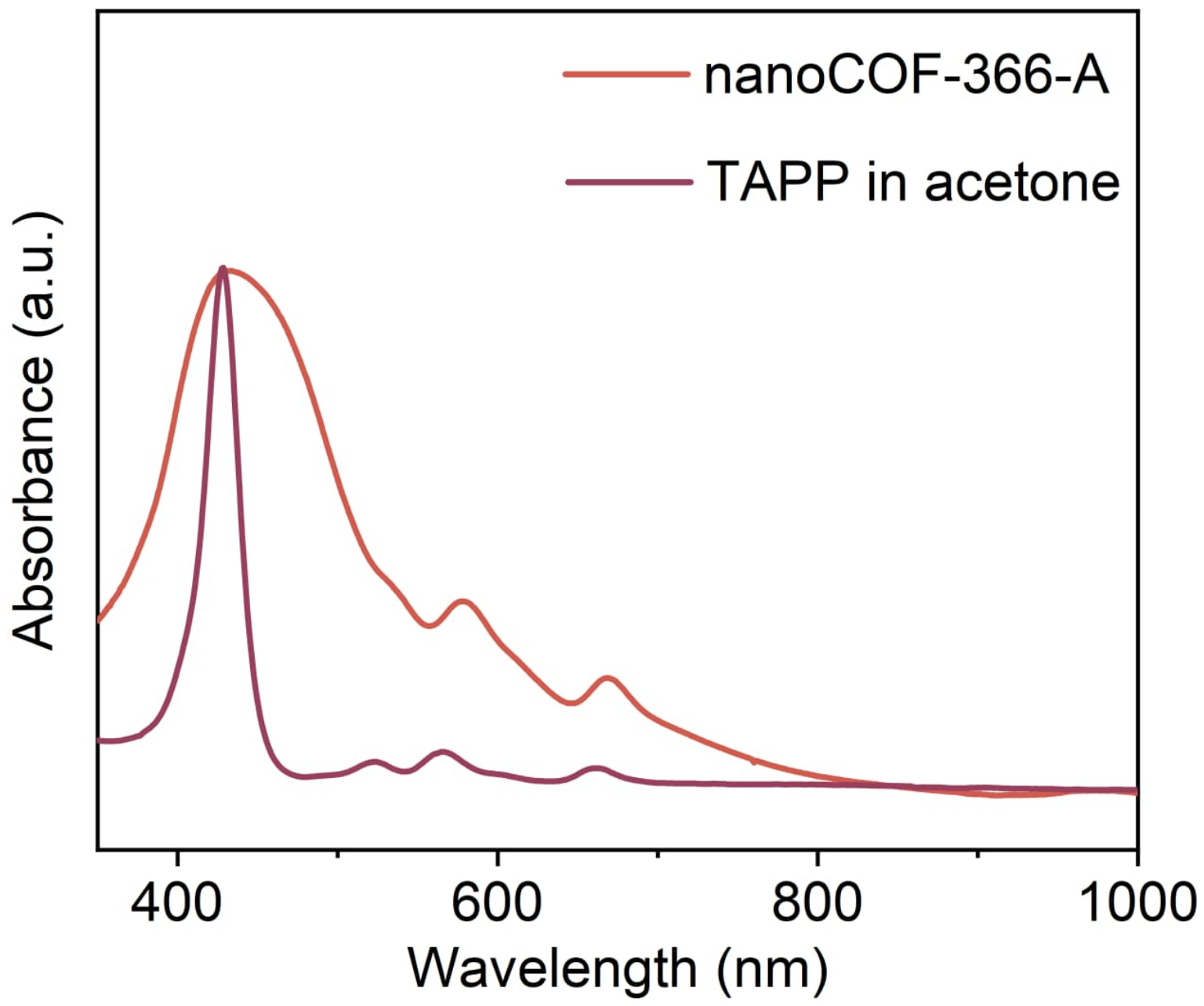

Figure S11. UV–Vis absorption spectra of TAPP and nanoCOF-366-A in acetone.

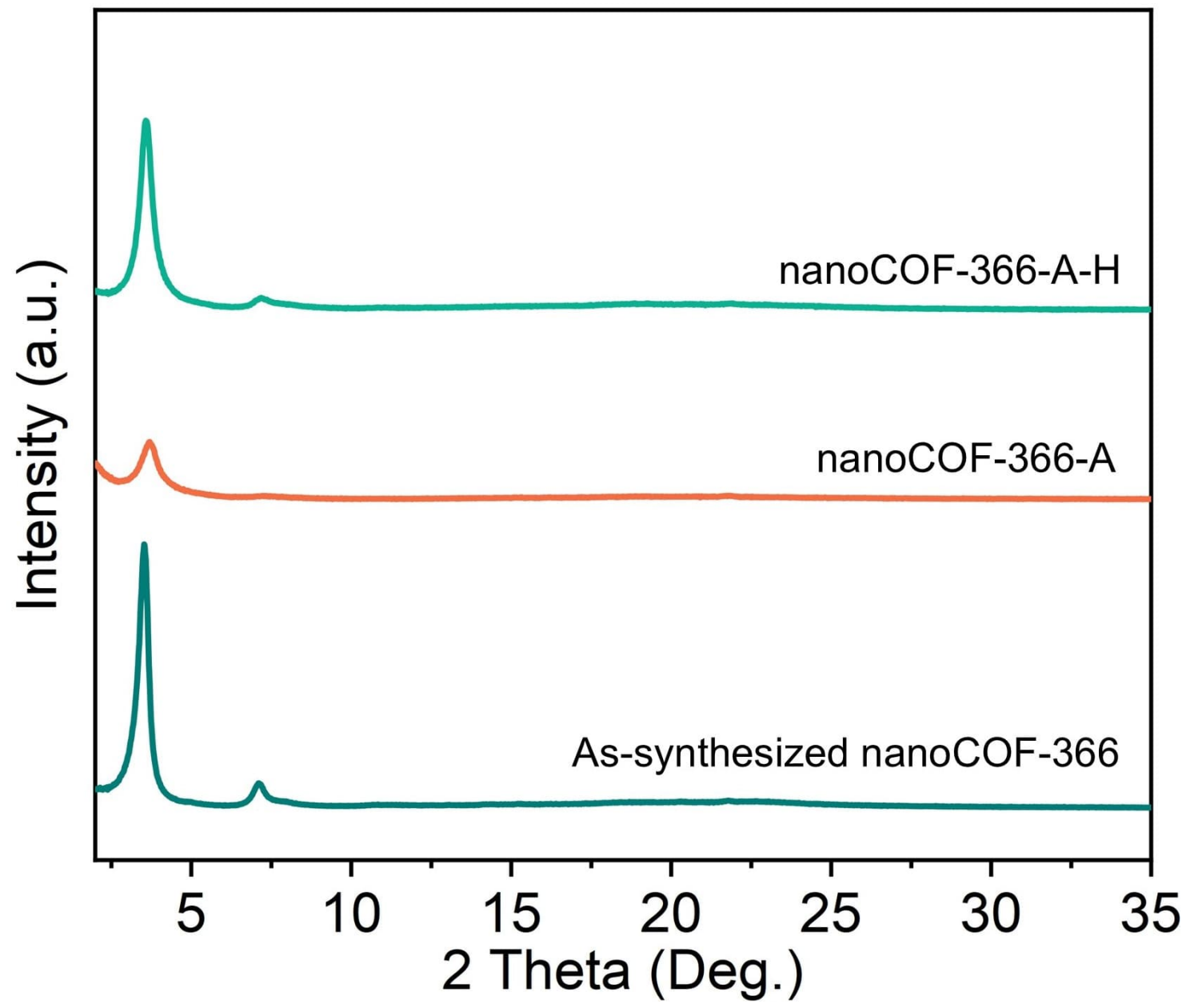

Figure S12. PXRD patterns of as-synthesized nanoCOF-366 (bottom), acetone-treated nanoCOF-366 (nanoCOF-366-A, middle), and water-treated nanoCOF-366-A (nanoCOF-366-A-H). Treatment of as-synthesized nanoCOF-366 with acetone significantly reduced its crystallinity, while subsequent soaking of nanoCOF-366-A in water for a few days partially restored the crystallinity.

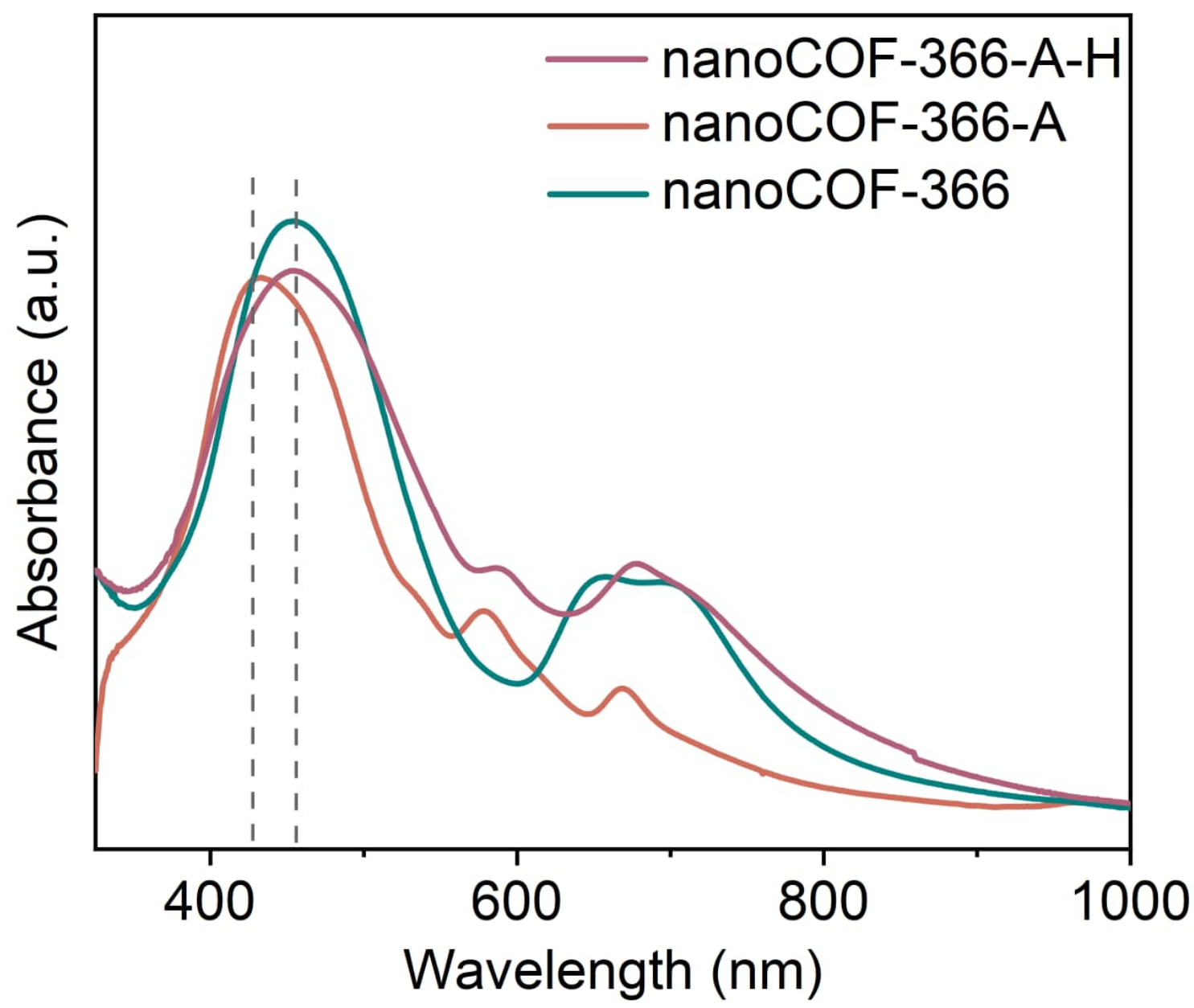

Figure S13. UV–Vis absorption spectra of aqueous suspensions of nanoCOF-366, nanoCOF-366-A, and nanoCOF-366-AH. Upon treatment of as-synthesized nanoCOF-

366 with acetone, the sample exhibited a blue-shifted spectrum accompanied by the emergence of several Q bands, indicating the deprotonation of protonated porphyrin units in nanoCOF-366. Subsequent treatment of nanoCOF-366-A with water resulted in a red shift of the absorption spectrum, indicating reprotonation of the porphyrin units.

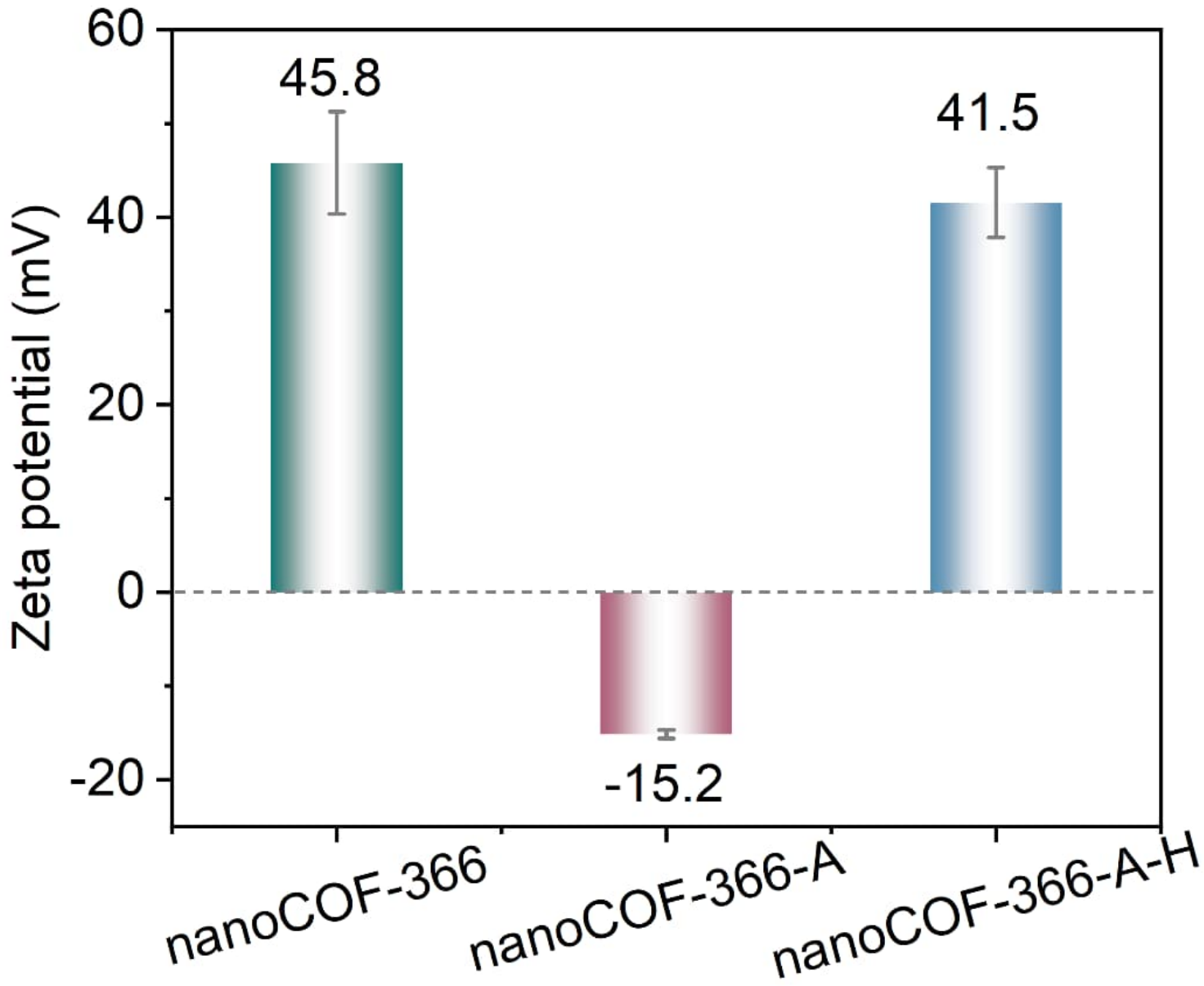

Figure S14. Zeta potentials of aqueous suspensions of nanoCOF-366, nanoCOF-366-A, and nanoCOF-366-A-H. NanoCOF-366 exhibits a high positive zeta potential due to protonation of the porphyrin units by acetic acid during synthesis. Upon treatment with acetone, the resulting nanoCOF-366-A sample shows a significantly decreased zeta potential, indicating a deprotonation process. Further treatment of nanoCOF-366-A with water leads to an increase in zeta potential, suggesting reprotonation of the porphyrin rings.

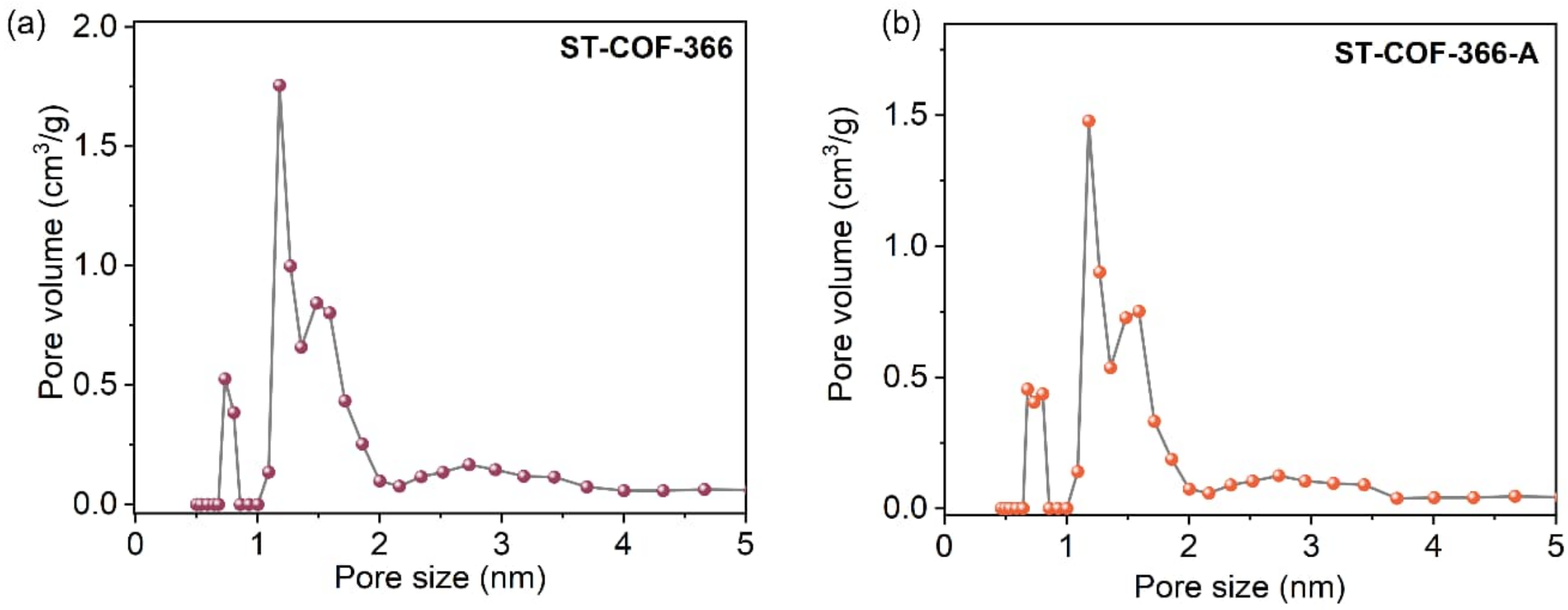

Figure S15. Pore size distributions of ST-COF-366 (a) and acetone-treated ST-COF-366 (b) calculated from the N_2 adsorption isotherms.

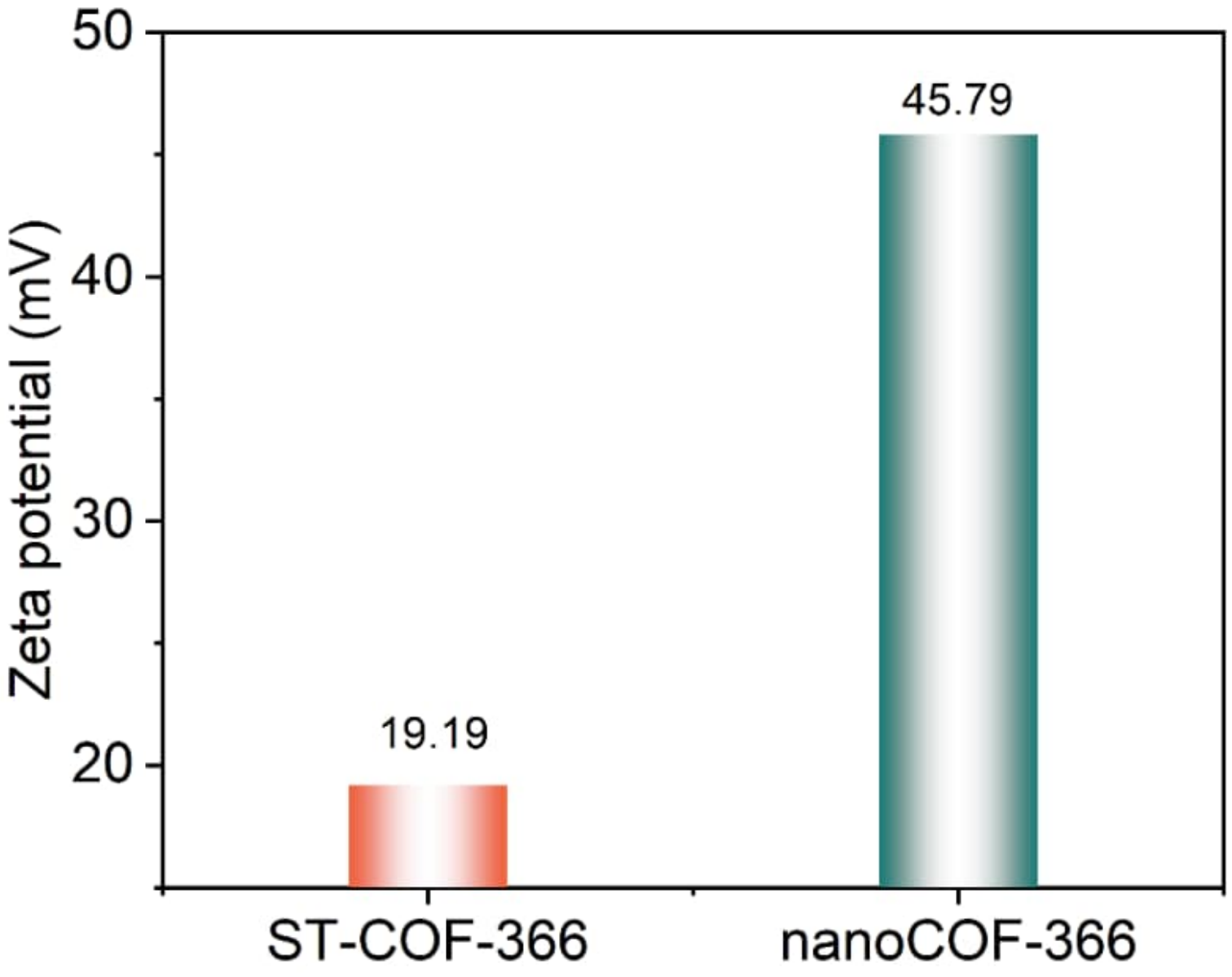

Figure S16. Comparison of Zeta potential of aqueous suspensions of ST-COF-366 and nanoCOF-366.

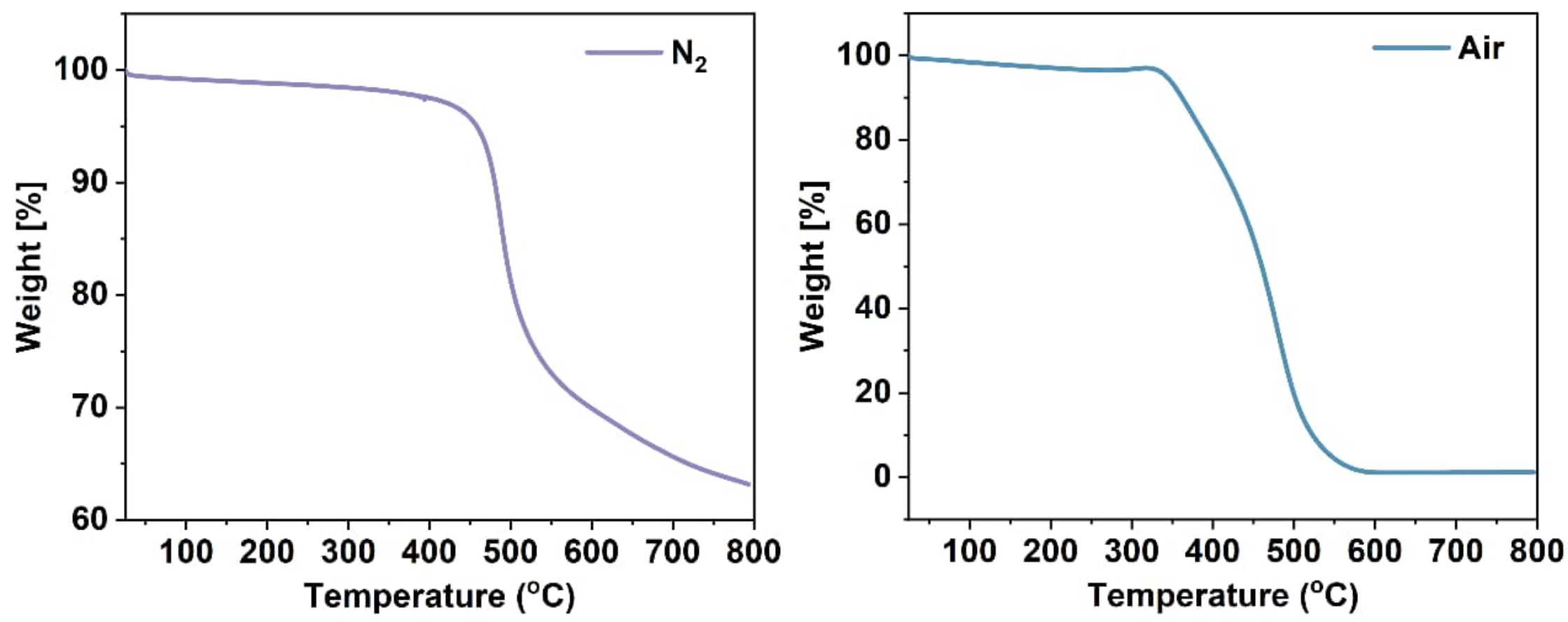

Figure S17. TGA curves of nanoCOF-366 recorded under N_2 (left) and air (right) atmospheres. The samples show no significant weight loss up to ~450 °C under N_2 and ~350 °C in air, indicating high thermal stability.

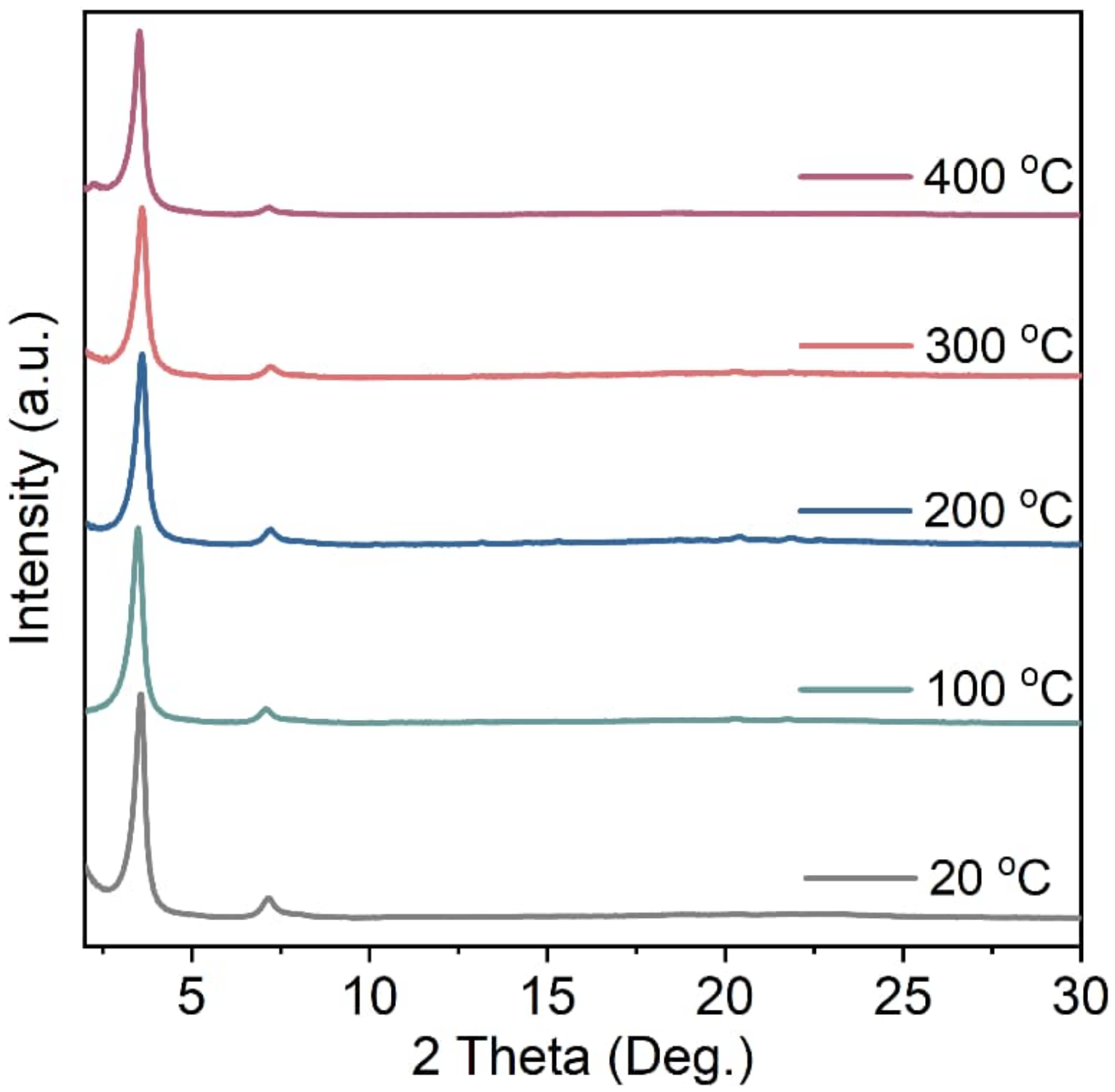

Figure S18. PXRD patterns of nanoCOF-366 after vacuum activation at temperatures from 20 to 400 °C. The results show that the sample retains excellent crystallinity after heating up to 400 °C under vacuum, indicating high structural stability and resistance to high temperatures.

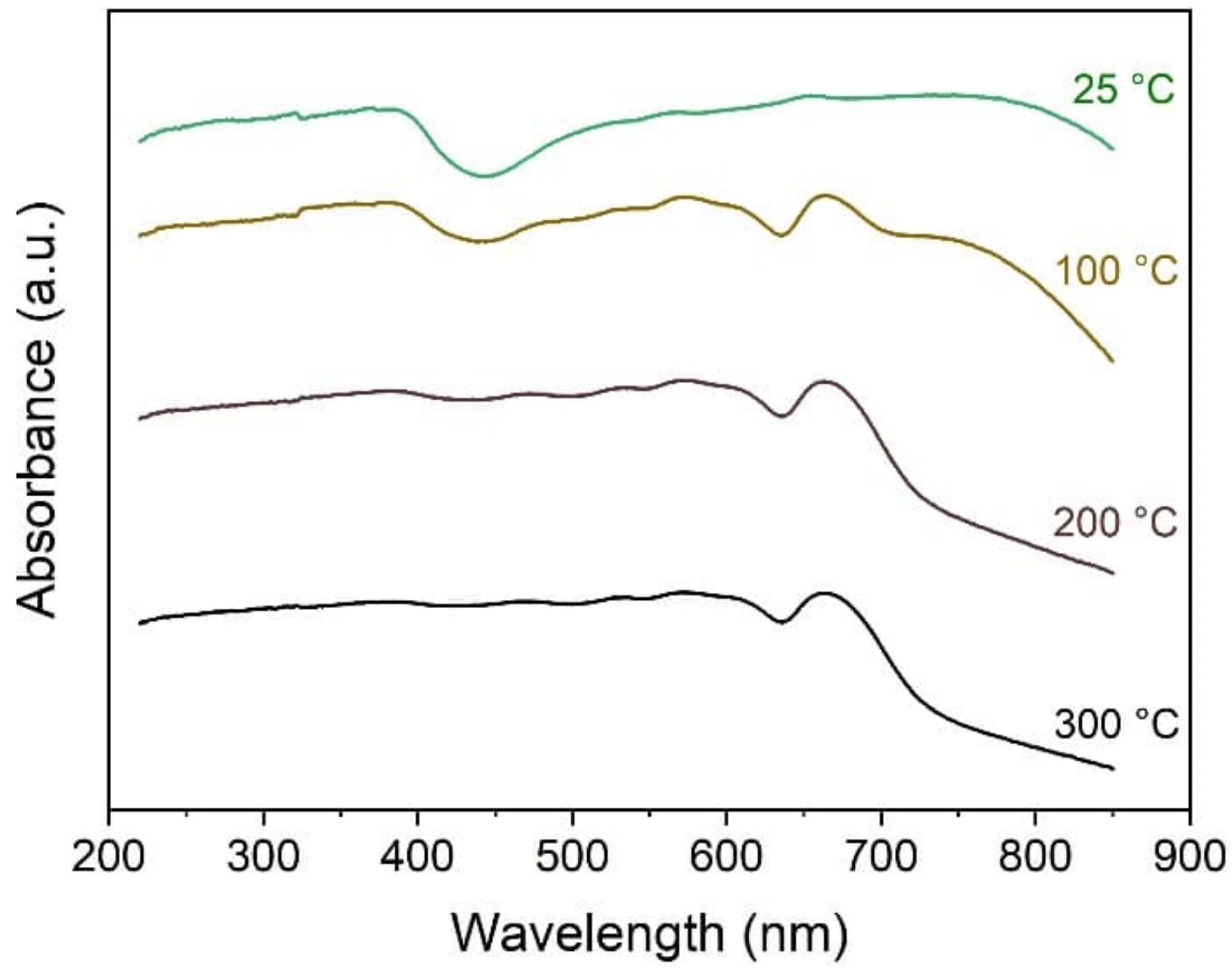

Figure S19. Solid-state UV–Vis absorption spectra of as-synthesized nanoCOF-366 after activation at different temperatures. The spectra show a blue shift with increasing heat-treatment temperature, accompanied by a color change from green to brown, indicating deprotonation of porphyrin units in nanoCOF-366.

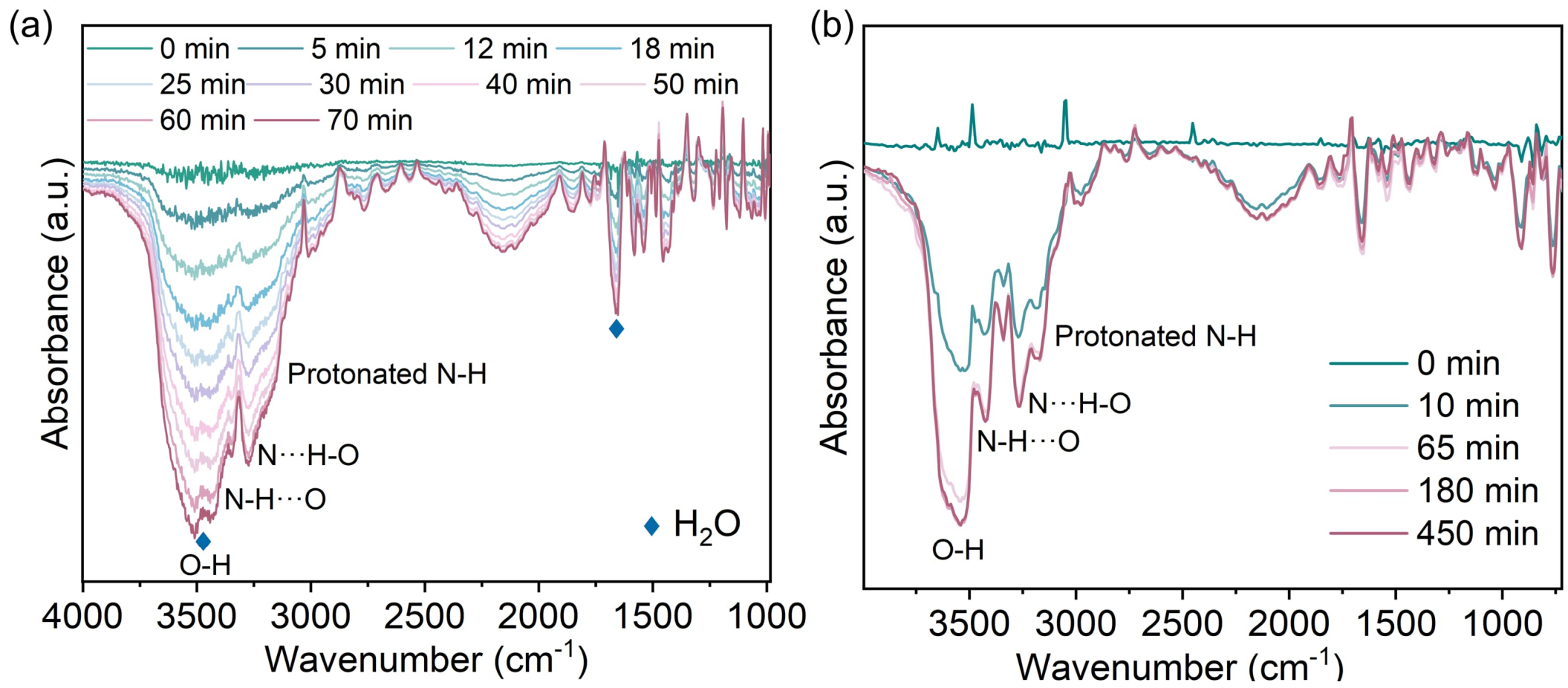

Figure S20. Time-resolved in situ infrared Fourier transform spectroscopy (DRIFTS) difference spectra of as-synthesized nanoCOF-366 during drying under Ar at room temperature. (a) The sample was dried in ambient air before measurement. (b) The sample was pre-flushed with Ar for 1 h prior to measurement; this Ar-treated sample was used as the background (0 min) to minimize the influence of the broad water peak and observe infrared changes during the continued Ar flow.

Additional discussion of the DRIFTS results: In situ DRIFTS was employed to monitor the structural evolution of nanoCOF-366 during activation as the temperature increased from 25 °C to 300 °C under Ar (Figure S20 and 3f). During drying at 25 °C, in addition to bands at ~3500 and ~1600 cm^{-1} associated with removal of physically adsorbed water, a broad band at 3400–3200 cm^{-1} appeared, which can be attributed to the disruption of hydrogen bonding between protonated porphyrins and water molecules. Subsequent in situ heating up to 300 °C was performed until no significant spectral changes were observed. Unlike the weak hydrogen-bonding interactions removed at low temperature, increasing the activation temperature facilitated the removal of protonated NH^+ sites and the strong interactions between these sites and counterions, such as OH^- and acetate (Figure 3f). This deprotonation reduces protonation-induced electrostatic interactions, allowing strong π–π stacking interactions to maintain the integrity of the ordered stacked structure. As a result, solvent intercalation between layers is suppressed, preserving the ordered stacked structure, similar to the behavior observed in non-protonated ST-COF-366.

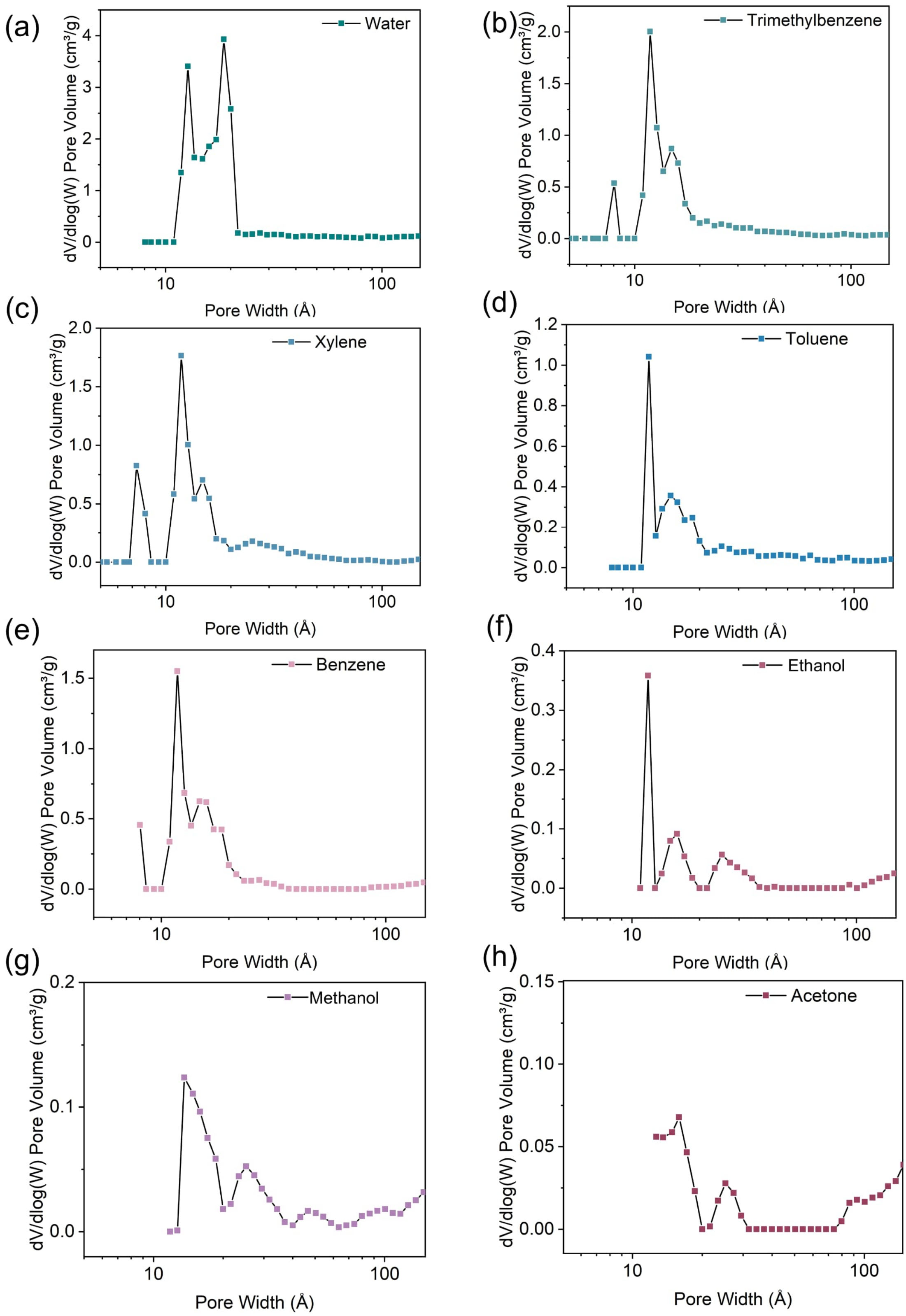

Figure S21. Pore size distributions of nanoCOF-366 after treatment with different solvents, calculated from their N_2 adsorption isotherms.

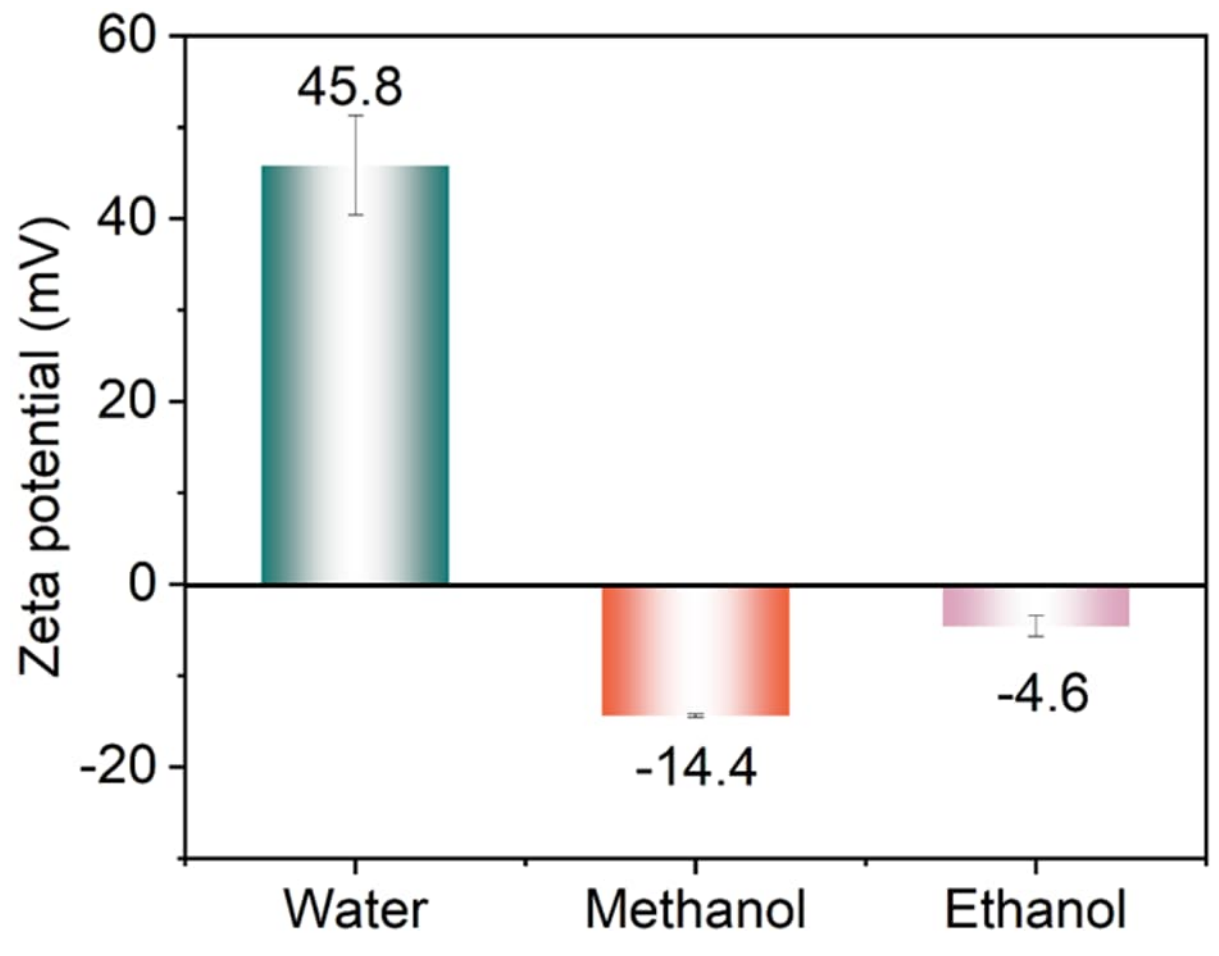

Figure S22. Zeta potential of aqueous suspension of nanoCOF-366 after treatment with methanol and ethanol.

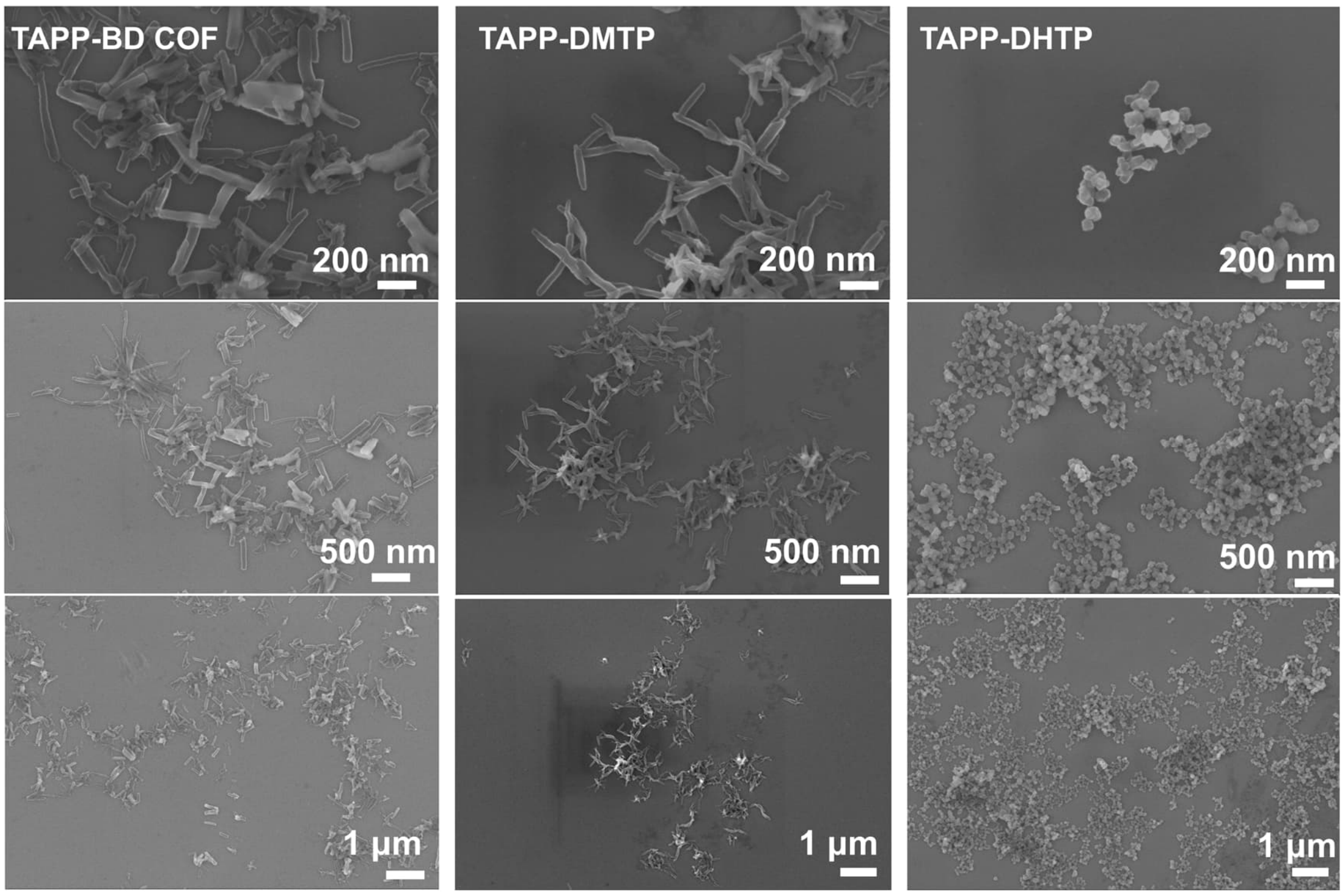

Figure S23. SEM images of as synthesized TAPP-BD COF, TAPP-DMTP COF and TAPP-DHTP COF. All samples display well-defined nanorod or nanocube morphologies.

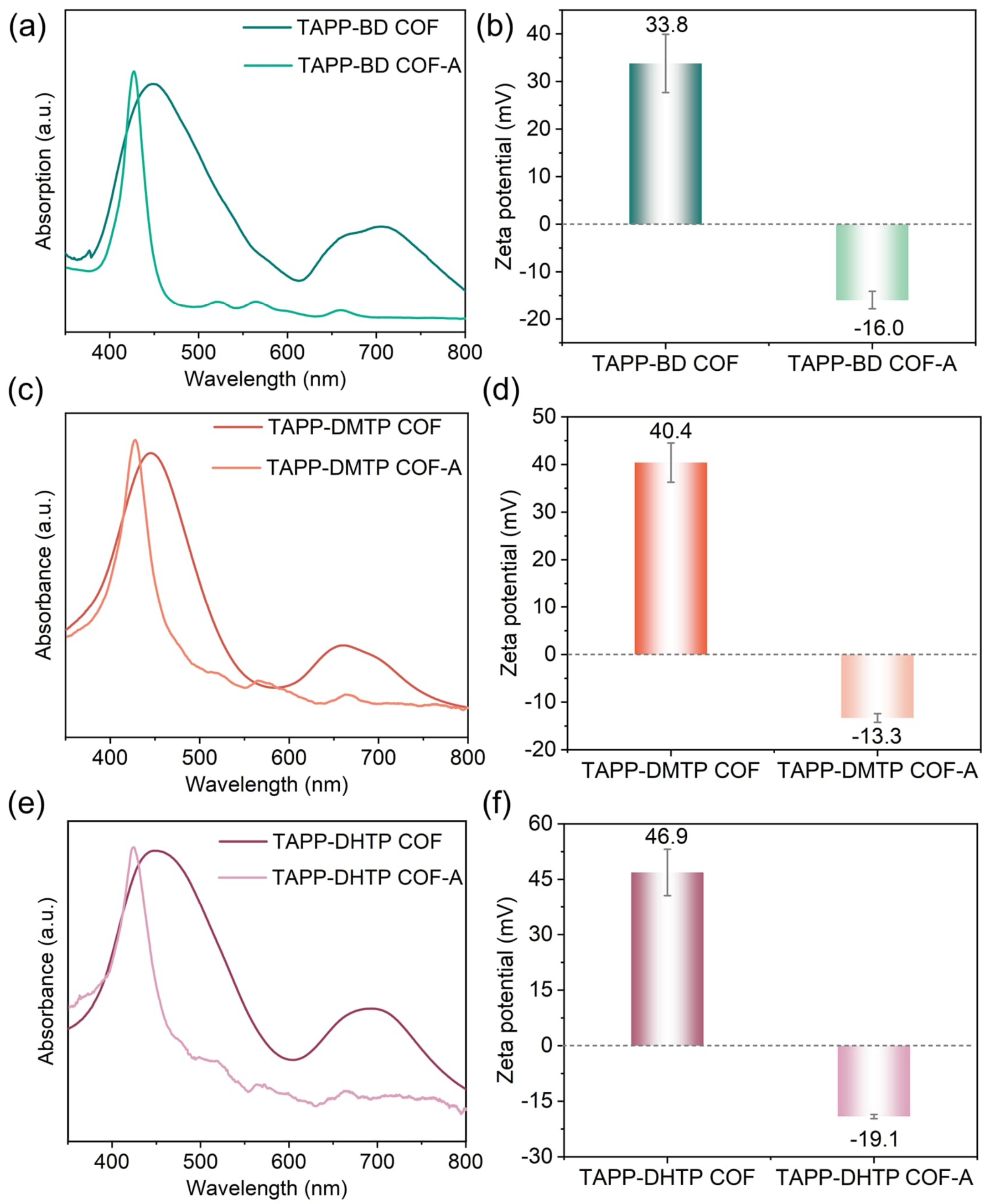

Figure S24. UV–Vis spectra (a–c) and zeta potentials (d–f) of as-synthesized TAPP-BD, TAPP-DMTP, and TAPP-DHTP COFs and their acetone-treated counterparts. The as-synthesized COFs show red-shifted UV–Vis absorption spectra compared to the TAPP linker and exhibit highly positive zeta potentials, indicating that the porphyrin units are protonated. Upon acetone treatment, the resulting samples display blue-shifted UV–Vis spectra and significantly decreased zeta potential values due to deprotonation. These results are consistent with those observed for nanoCOF-366.

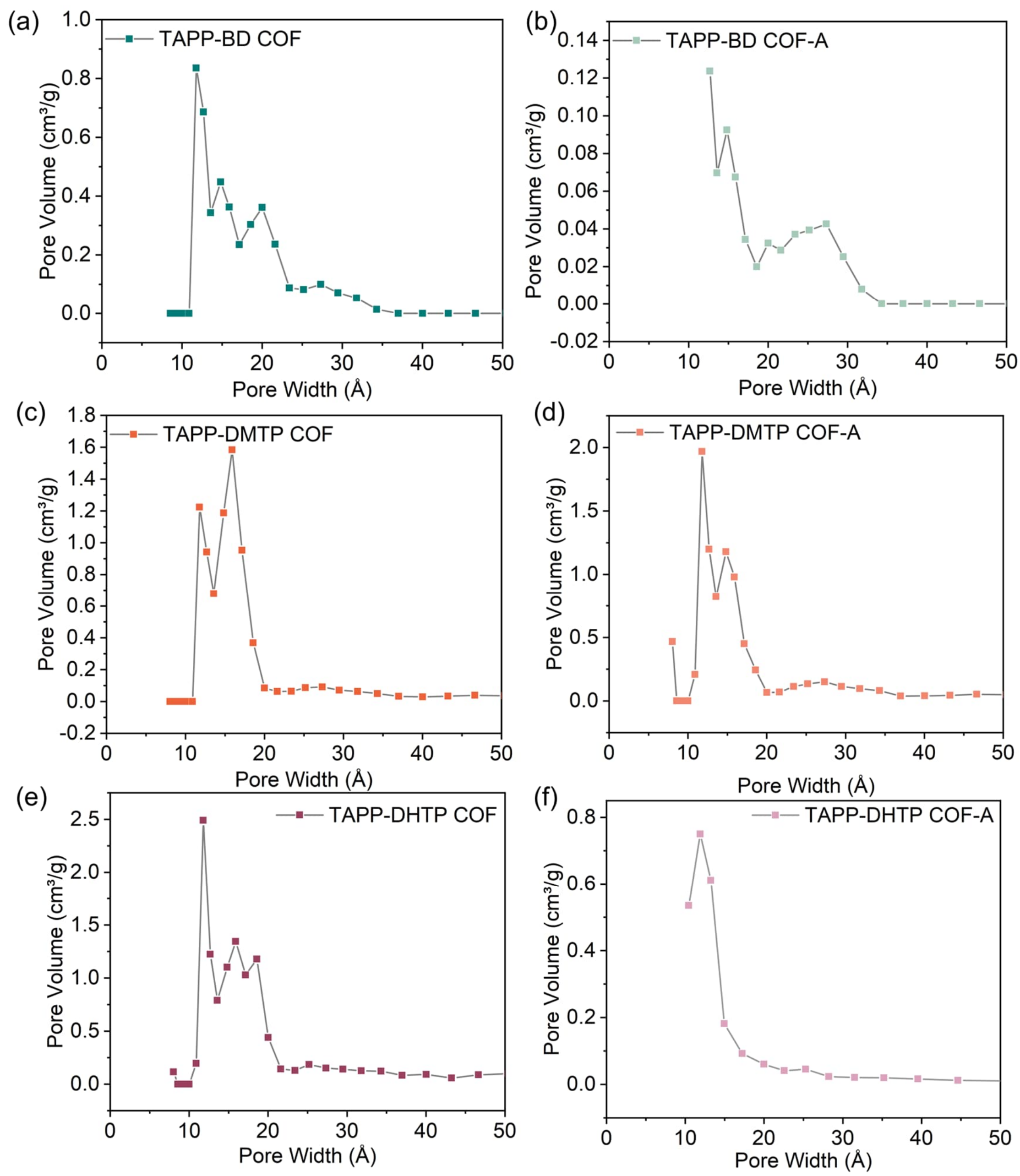

Figure S25. Pore size distributions of as-synthesized TAPP-BD COF, TAPP-DMTP COF, and TAPP-DHTP COF, and of their corresponding acetone-treated samples. The results were calculated from their N_2 adsorption isotherms.

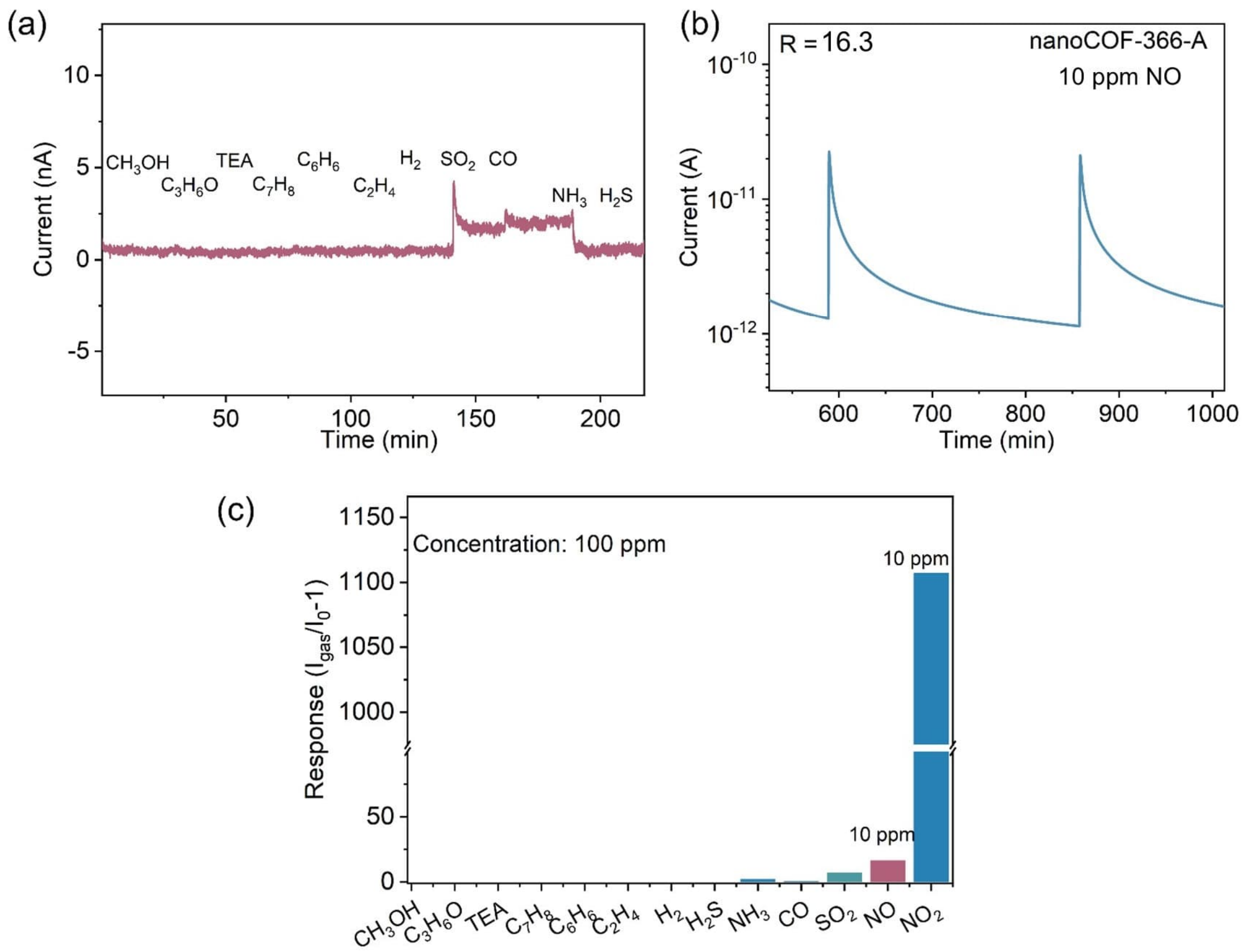

Figure 26. (a) Current responses of the nanoCOF-366-A sensor upon exposure to different analyte gases (100 ppm) diluted in air and (b) 10 ppm NO_2 diluted in N_2. (c) Sensor responses of nanoCOF-366-A toward different analyte gases, demonstrating its high selectivity for NO_2 detection.

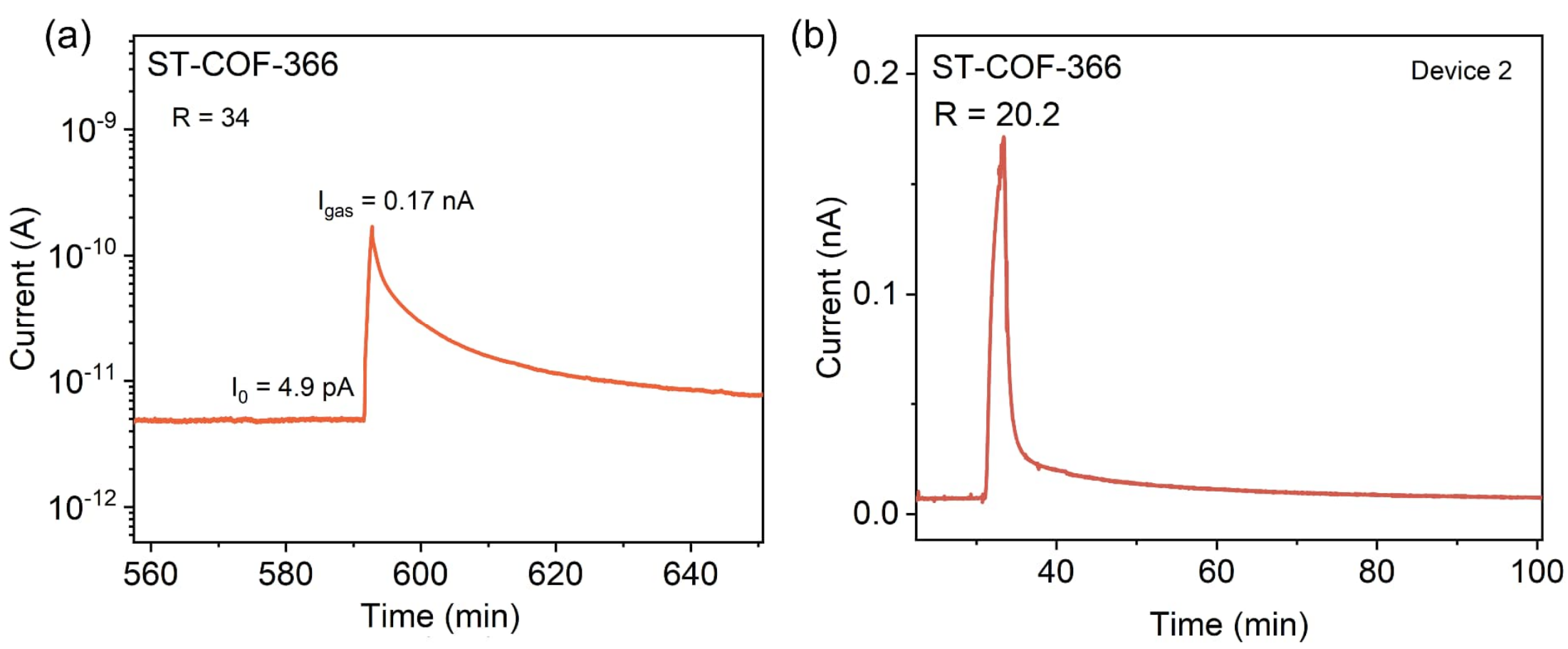

Figure S27. Response–recovery curve of the ST-COF-366 sensing device upon exposure to 10 ppm NO_2. Two parallel devices were prepared and tested under the same conditions to demonstrate reproducibility.

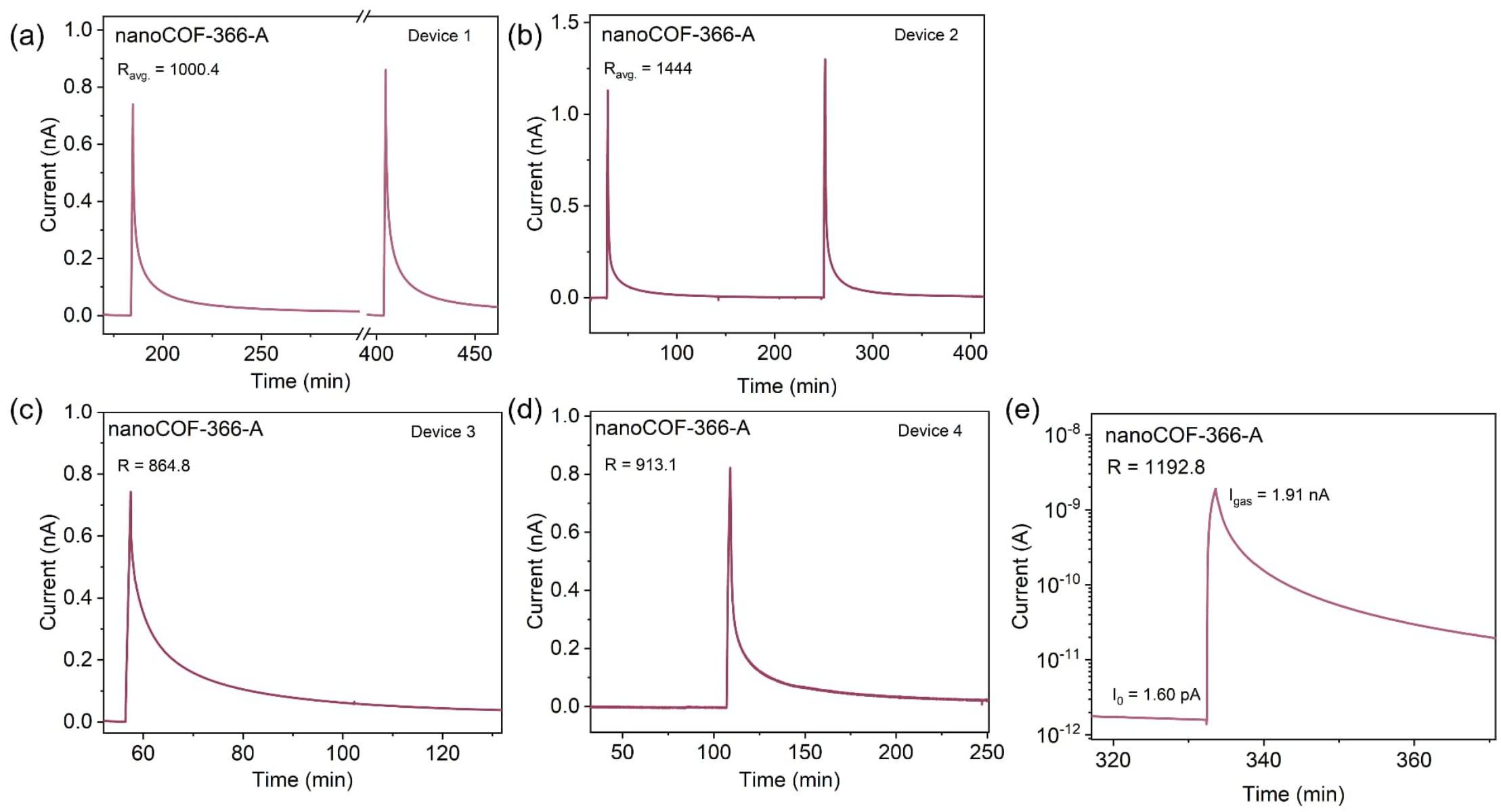

Figure S28. Response–recovery curve of nanoCOF-366-A upon exposure to 10 ppm NO_2. Five identical devices were prepared and tested under the same conditions. The panel in the top right shows three consecutive measurements, demonstrating good cyclic performance with a low coefficient of variation.

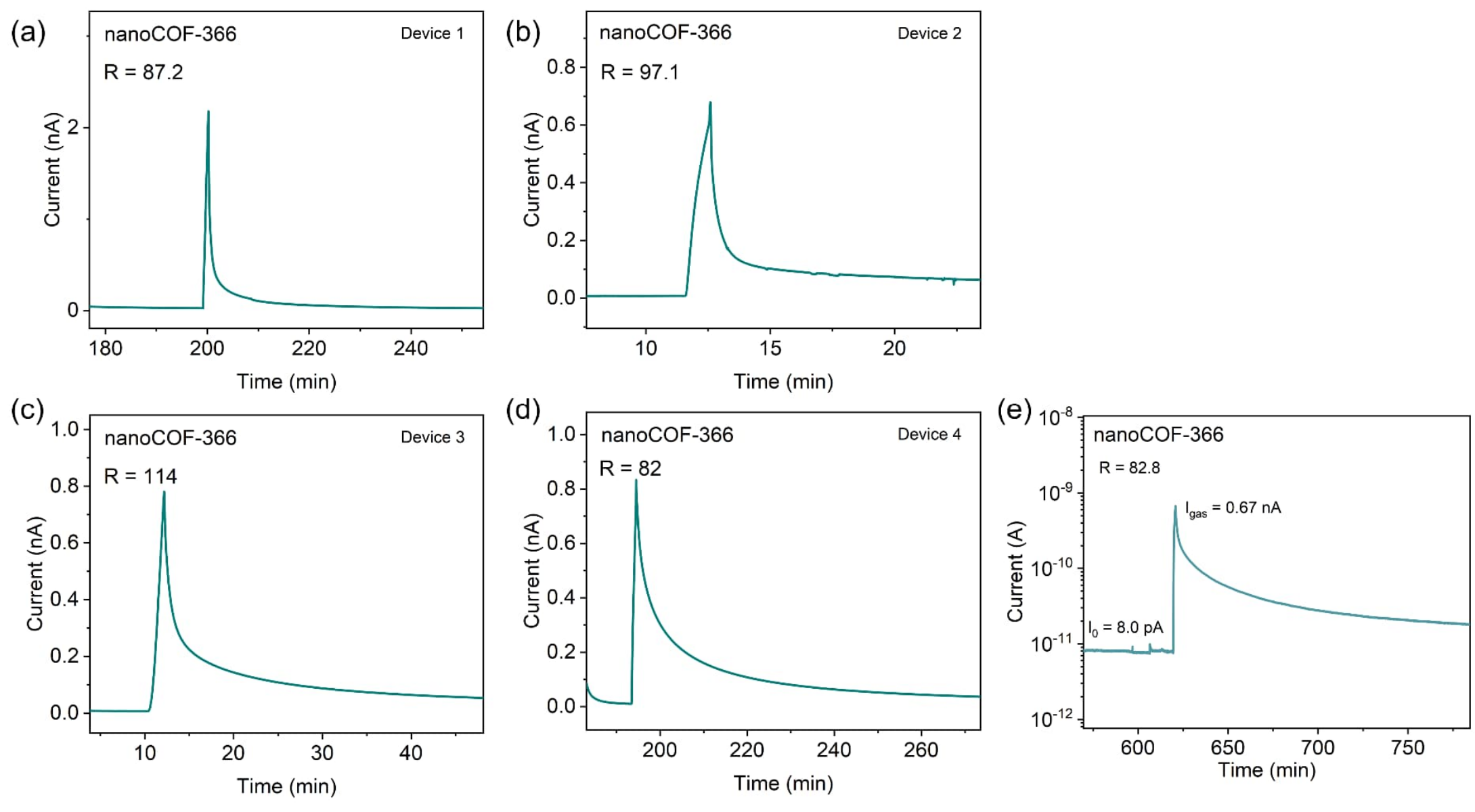

Figure S29. Response–recovery curve of the nanoCOF-366 sensing device upon exposure to 10 ppm NO_2. Five identical devices were prepared and tested under the same conditions to demonstrate reproducibility.

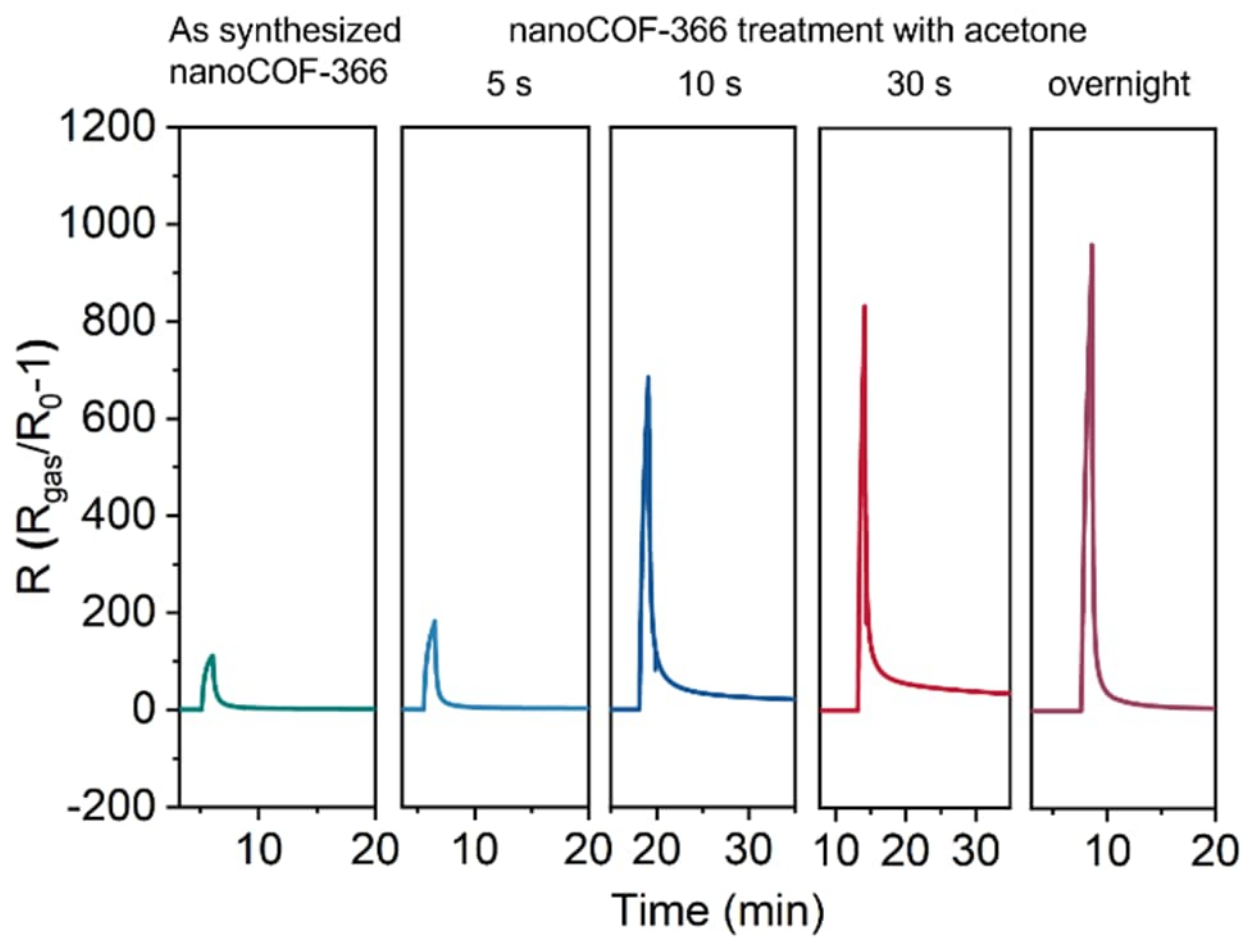

Figure S30. Chemiresistive NO_2 sensing responses of nanoCOF-366 samples treated with acetone for different durations. The NO_2 sensing response of nanoCOF-366 gradually increases with increasing acetone treatment time. The pristine nanoCOF-366 exhibits a response of approximately 100 toward 10 ppm NO_2, while the response increases to approximately 200, 700, and 800 after acetone treatments for 5, 10, and 30 s, respectively. Prolonged acetone exposure overnight results in a further increase of the response to approximately 1000. These results are consistent with the corresponding structural evolution observed by PXRD, N_2 adsorption, and TEM analyses (Figure 2). Longer acetone treatment induces progressively greater disruption of the ordered interlayer stacking structure, leading to enhanced exposure of previously less accessible porphyrin sites. Meanwhile, prolonged solvent treatment induces further deprotonation of the porphyrin centers, increasing their electron-rich character and facilitating charge transfer with adsorbed NO_2 molecules. Consequently, the enhanced accessibility of active nitrogen sites and improved charge-transfer interactions collectively contribute to the gradual increase in NO_2 sensing response.

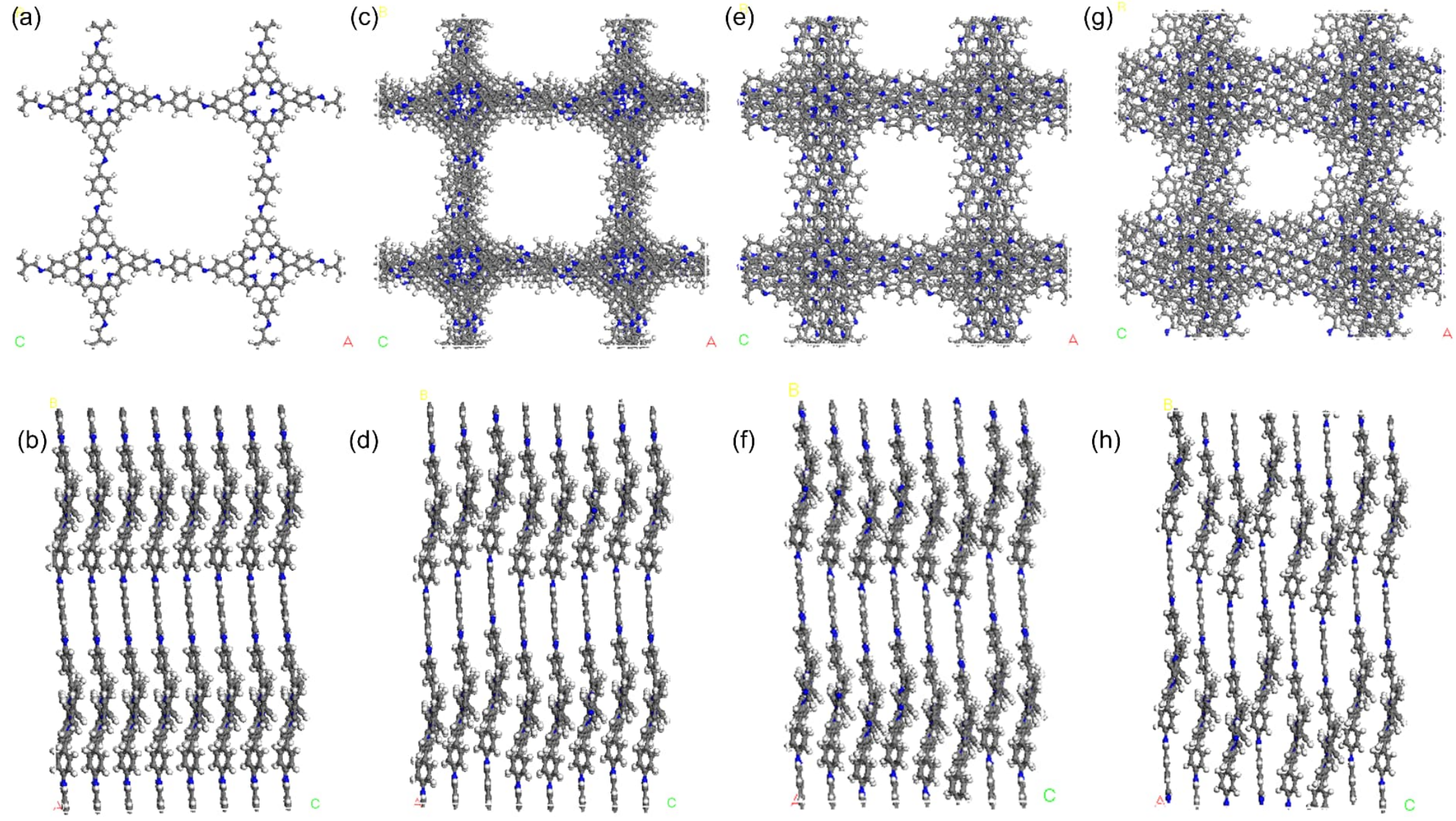

Figure S31. Illustration of the COF-366 structure showing varying layer displacements relative to the fully eclipsed AA stacking: 0 Å (a, b), 1–2 Å (c, d), 2–4 Å (e, f), and 3–5 Å (g, h).

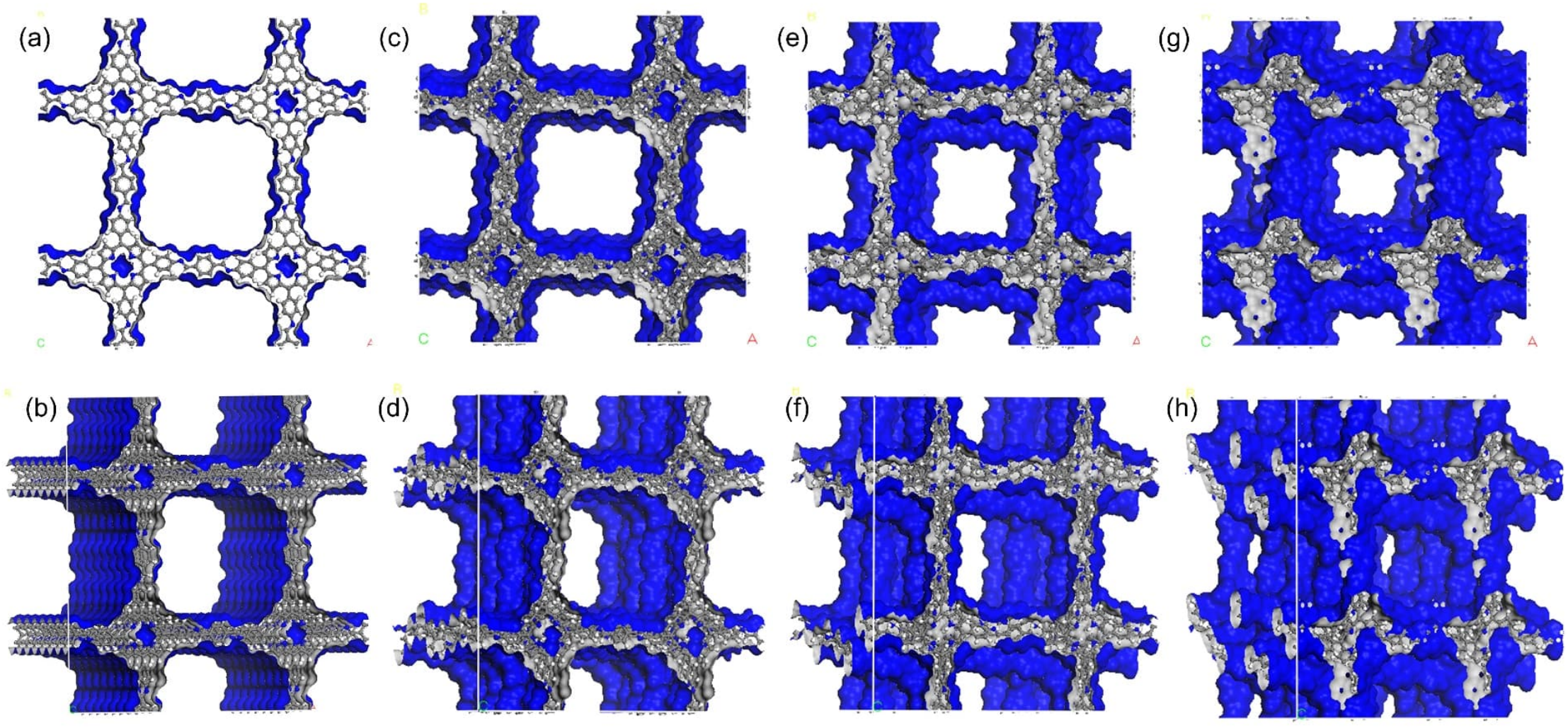

Figure S32. Layer stacking of COF-366 with varying displacements relative to the fully eclipsed AA stacking: 0 Å (a, b), 1–2 Å (c, d), 2–4 Å (e, f), and 3–5 Å (g, h). The accessible adsorption surfaces for NO_2 are highlighted in blue. Increasing layer displacement leads to greater structural disorder and enhanced accessible surface area.

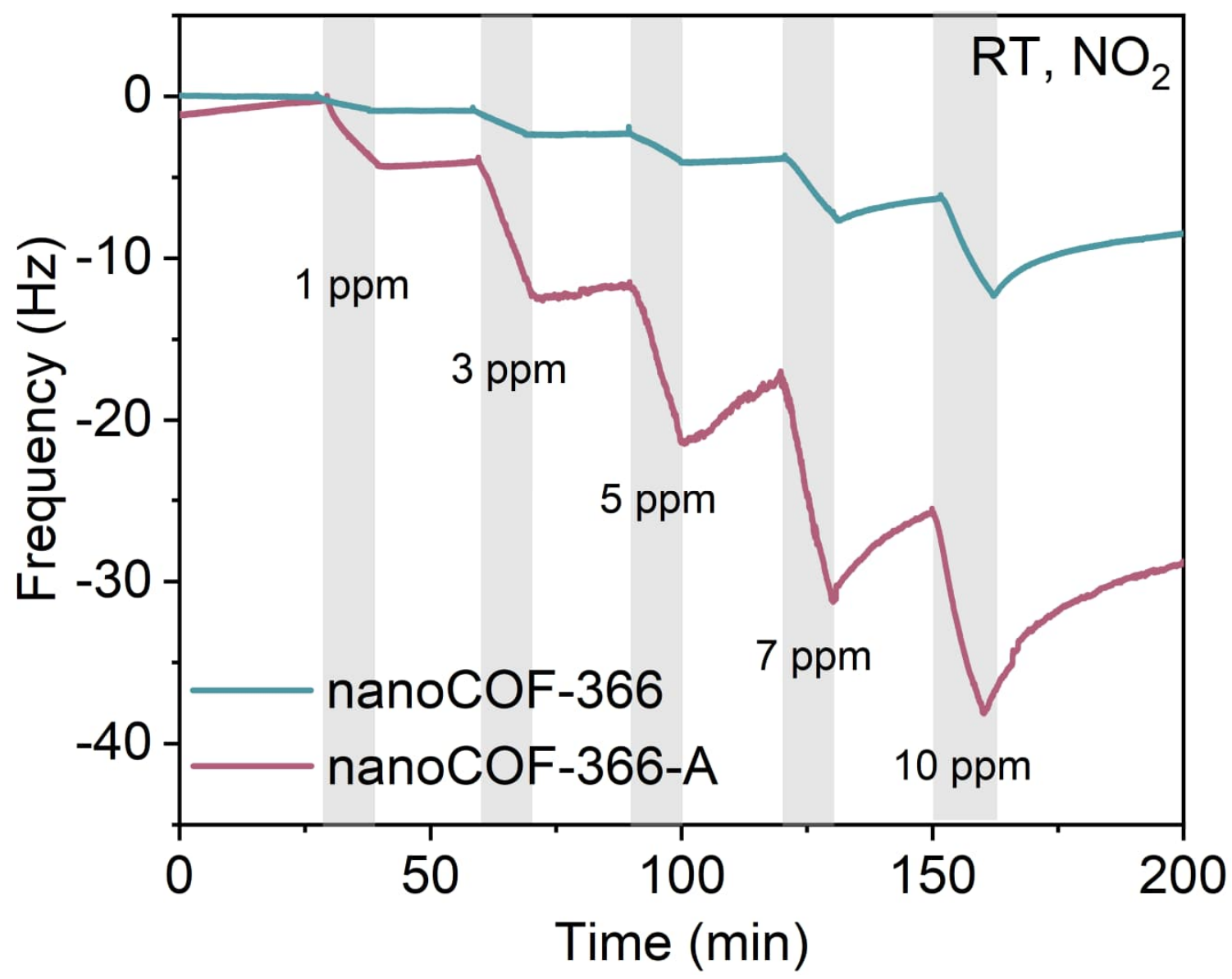

Figure S33. Real-time NO_2 sensing by nanoCOF-366 and nanoCOF-366-A using a MEMS resonant microcantilever, which monitors adsorption-induced resonance frequency shifts for high-sensitivity detection. Measurements were performed at room temperature under NO_2 concentrations of 1–10 ppm. The corresponding NO_2 adsorption capacities, derived from these shifts, are shown in Figure 5c.

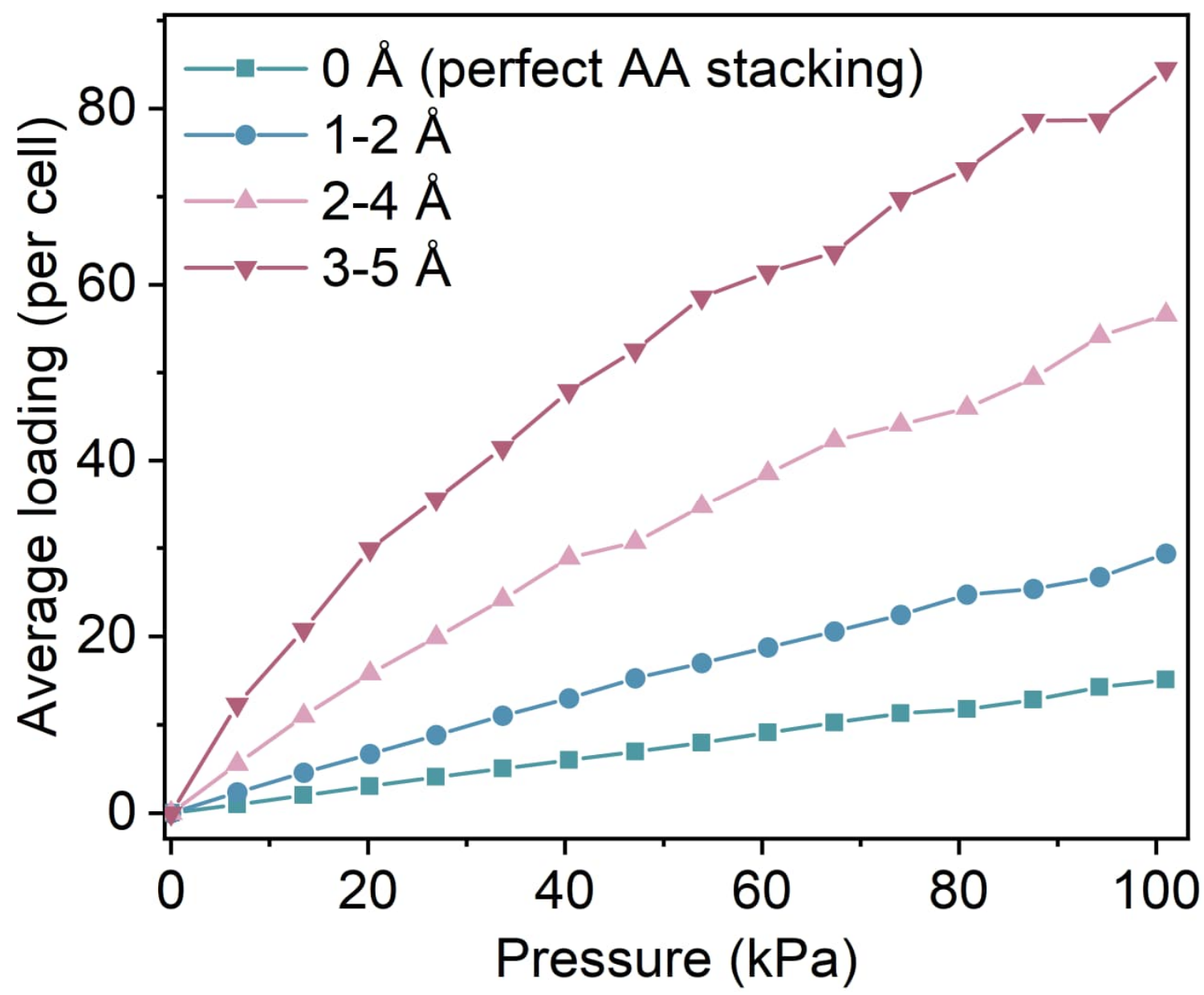

Figure S34. Simulated NO_2 adsorption isotherms on COF-366 at 298 K for different layer displacements relative to the fully eclipsed AA stacking (0 Å), including 1–2 Å, 2–4 Å, and 3–5 Å offsets.

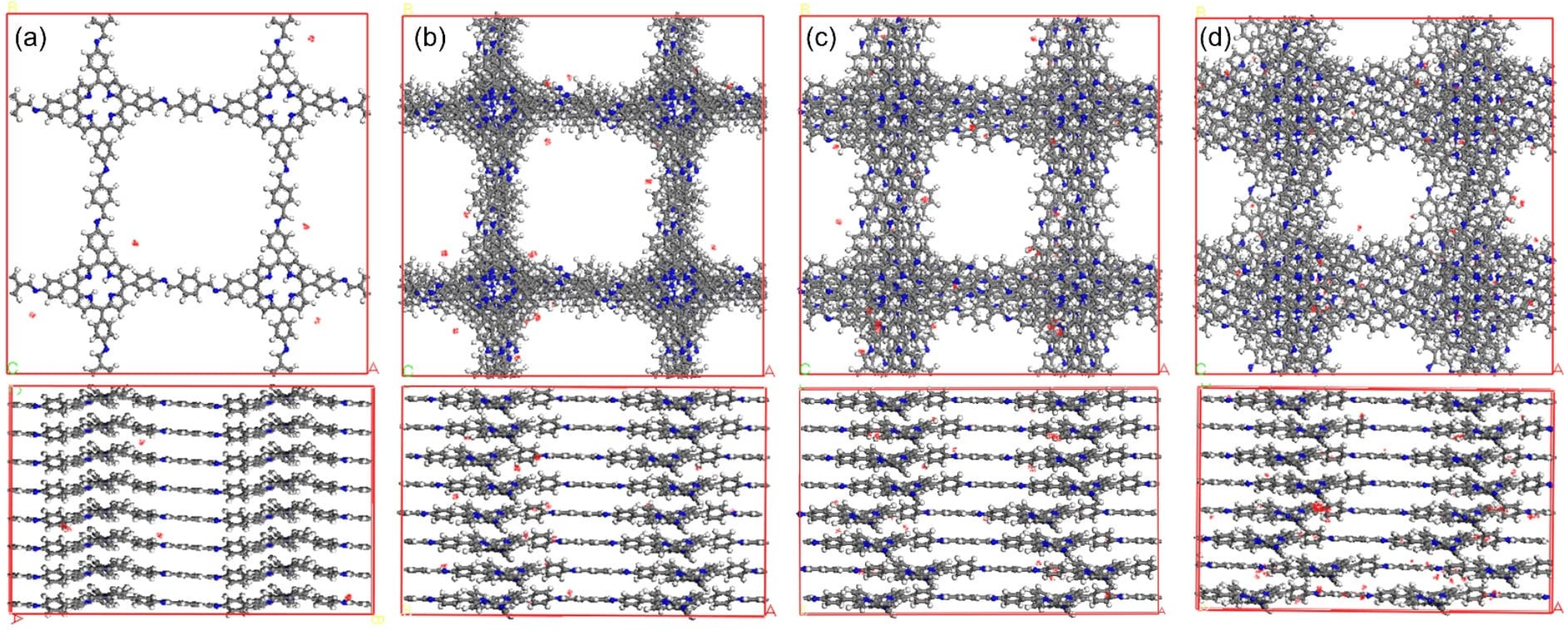

Figure S35. Snapshots of NO_2 adsorption (0.001 Pa, ~10 ppm) on COF-366 structures with layer displacements of 0 Å (a), 1–2 Å (b), 2–4 Å (c), and 3–5 Å (d). Red spheres represent NO_2 molecules.

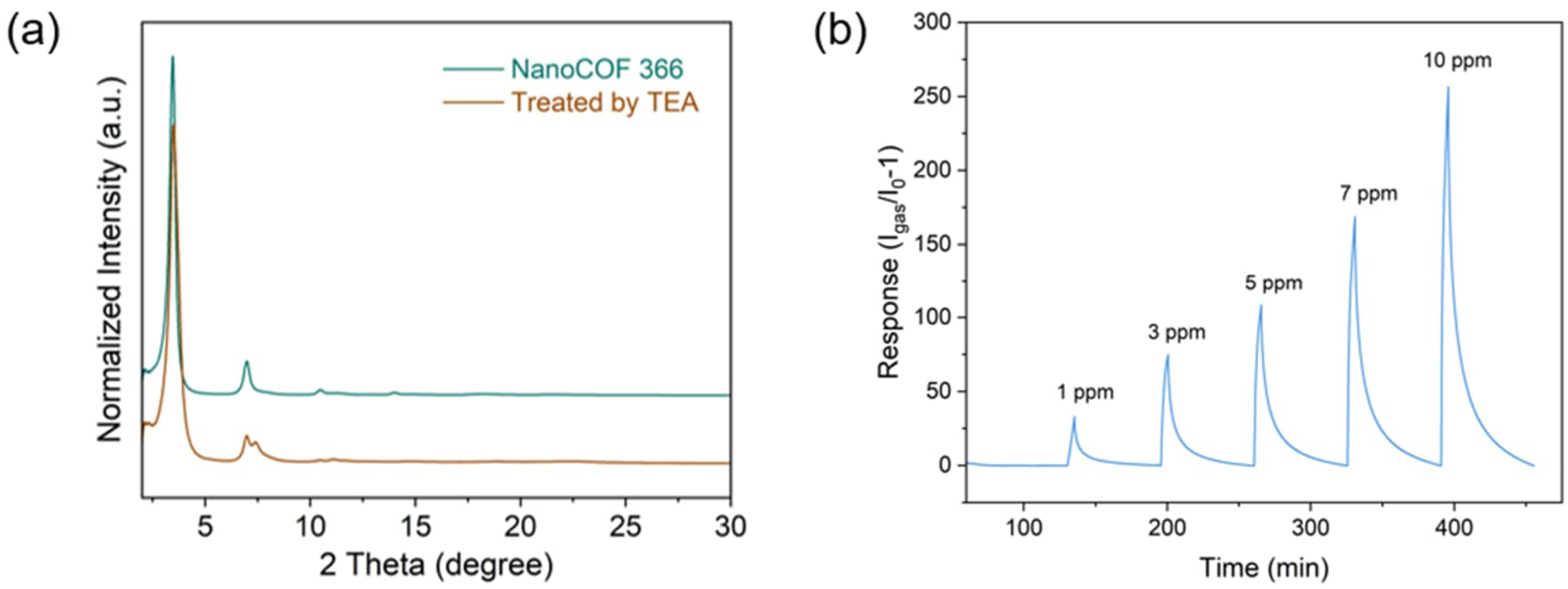

Figure S36. (a) XRD patterns of as synthesized nanoCOF-366 and the sample treated by 50% aqueous triethylamine (TEA) solution. (b) Dynamic response curves of TEA-treated nanoCOF-366-A toward 1-10 ppm NO_2. The preserved structural integrity after TEA treatment is likely attributed to the formation of protonated triethylammonium species (Et_3NH^+), which remain within the interlayer regions and maintain interlayer stabilization through hydrogen-bonding and electrostatic interactions.

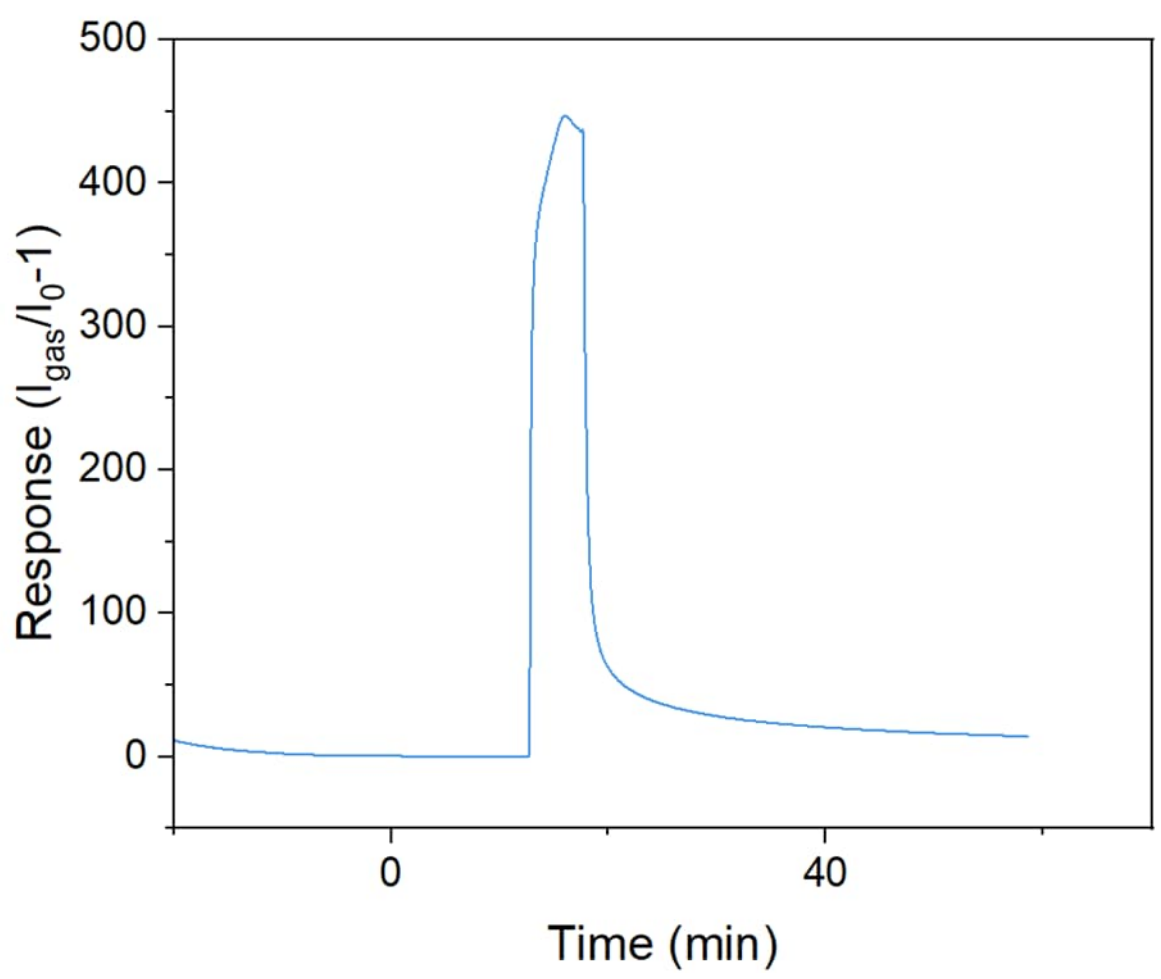

Figure S37. Response–recovery curves to 10 ppm NO_2 of TAPP linker.

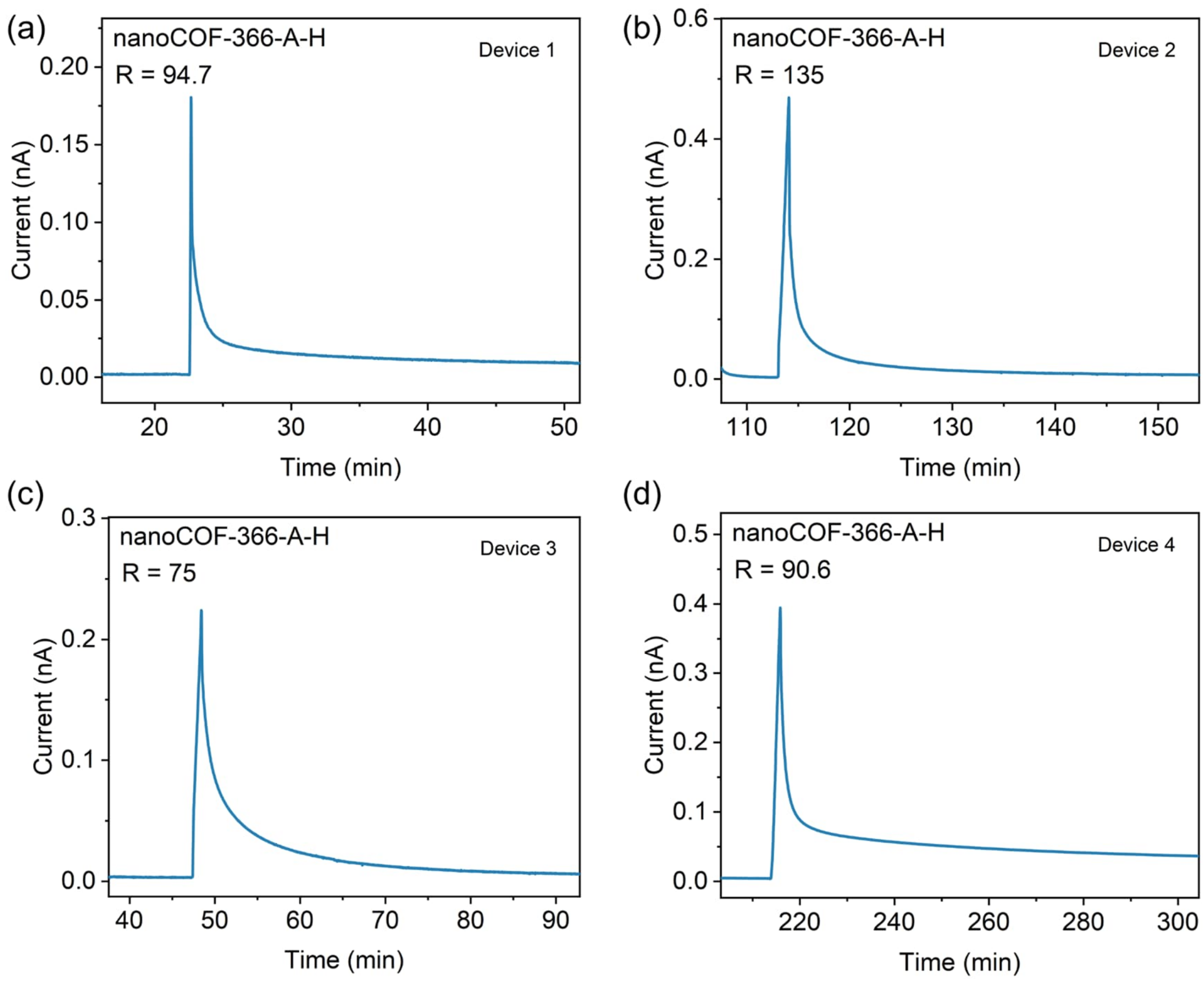

Figure S38. Response–recovery curve of the nanoCOF-366-A-H sensing device upon exposure to 10 ppm NO_2. Four identical devices were fabricated and tested under the same conditions to confirm reproducibility.

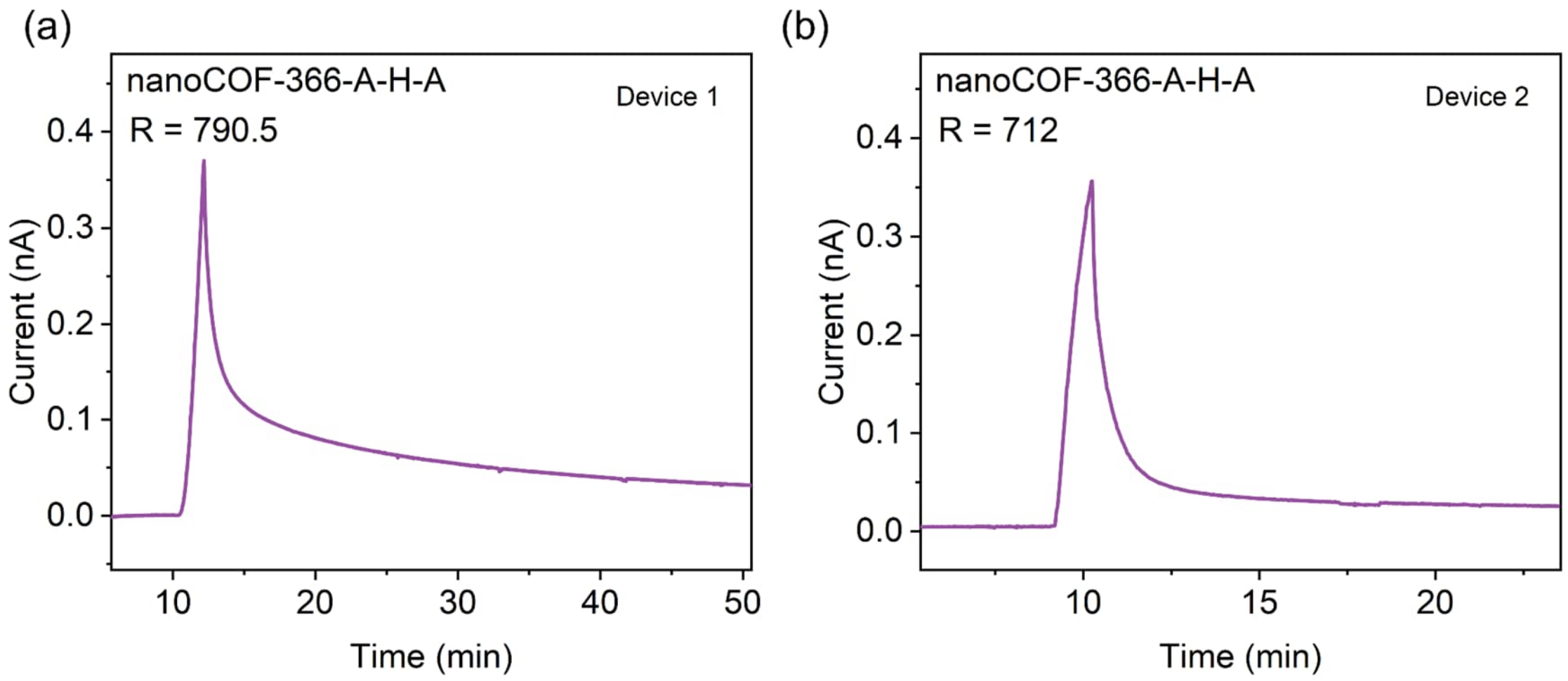

Figure S39. Response–recovery curve of the nanoCOF-366-AHA sensing device upon exposure to 10 ppm NO_2. Two identical devices were fabricated and tested under the same conditions to confirm reproducibility.

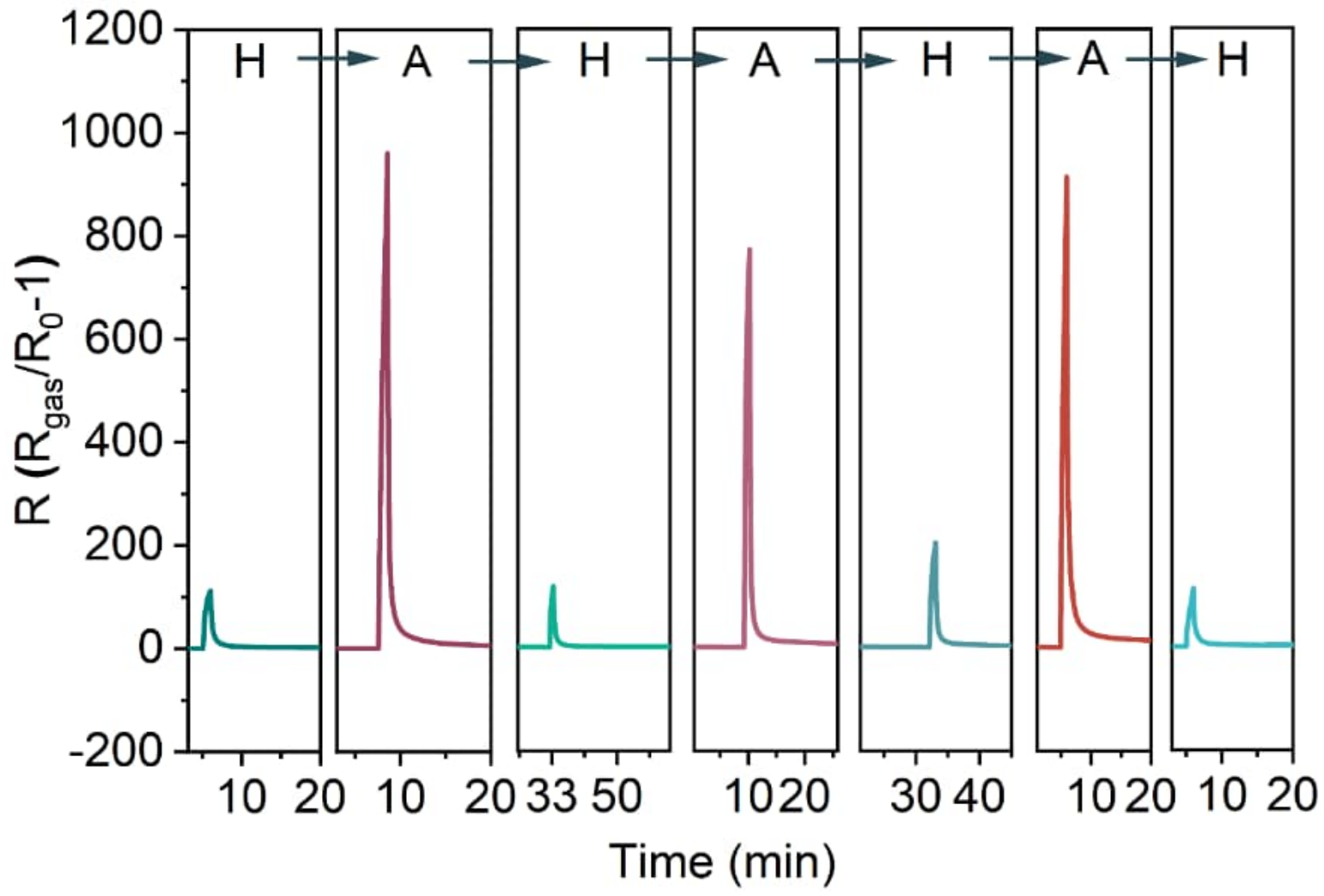

Figure S40. Comparison of the NO_2 sensing responses of nanoCOF-366 samples subjected to three consecutive cycles of alternating water (H) and acetone (A) treatments. The sensing responses were measured upon exposure to 10 ppm NO_2.

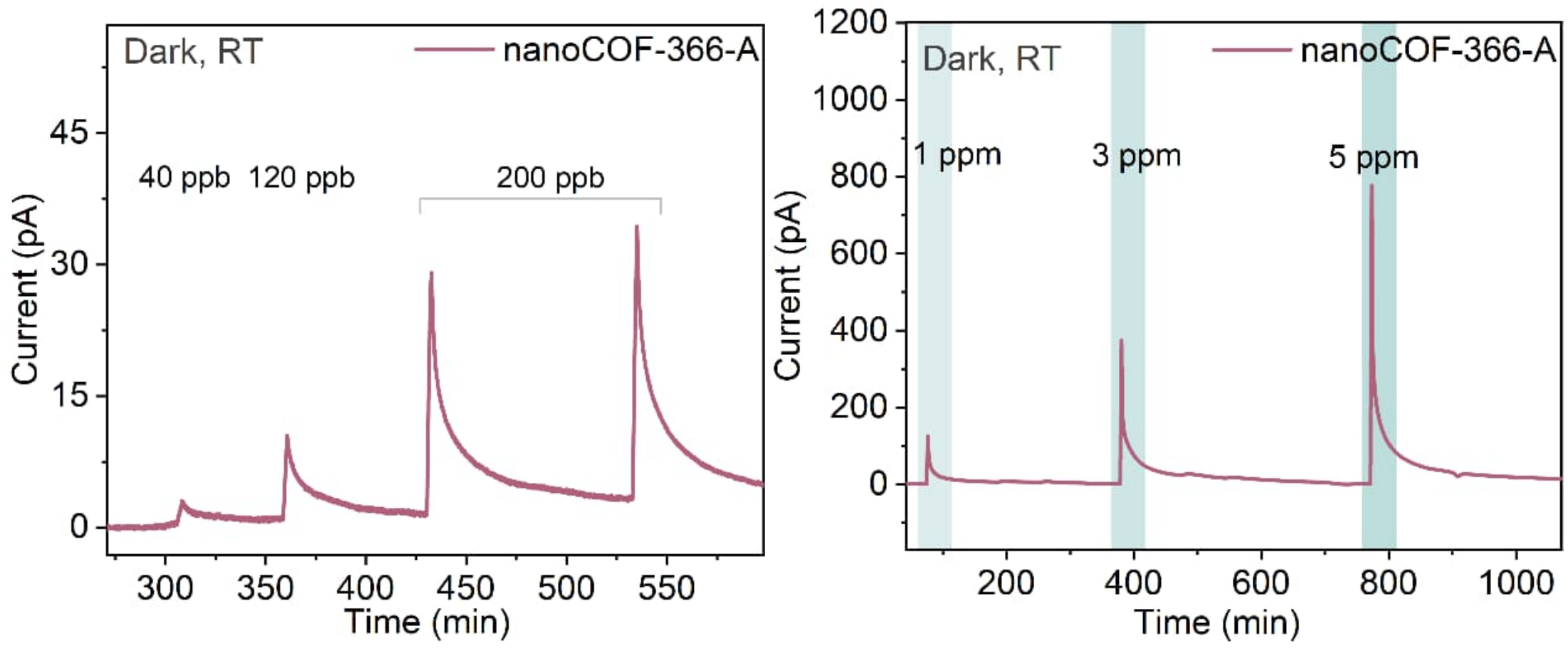

Figure S41. Response–recovery curves of nanoCOF-366-A upon exposure to NO_2 at concentrations ranging from 40 ppb to 5 ppm.

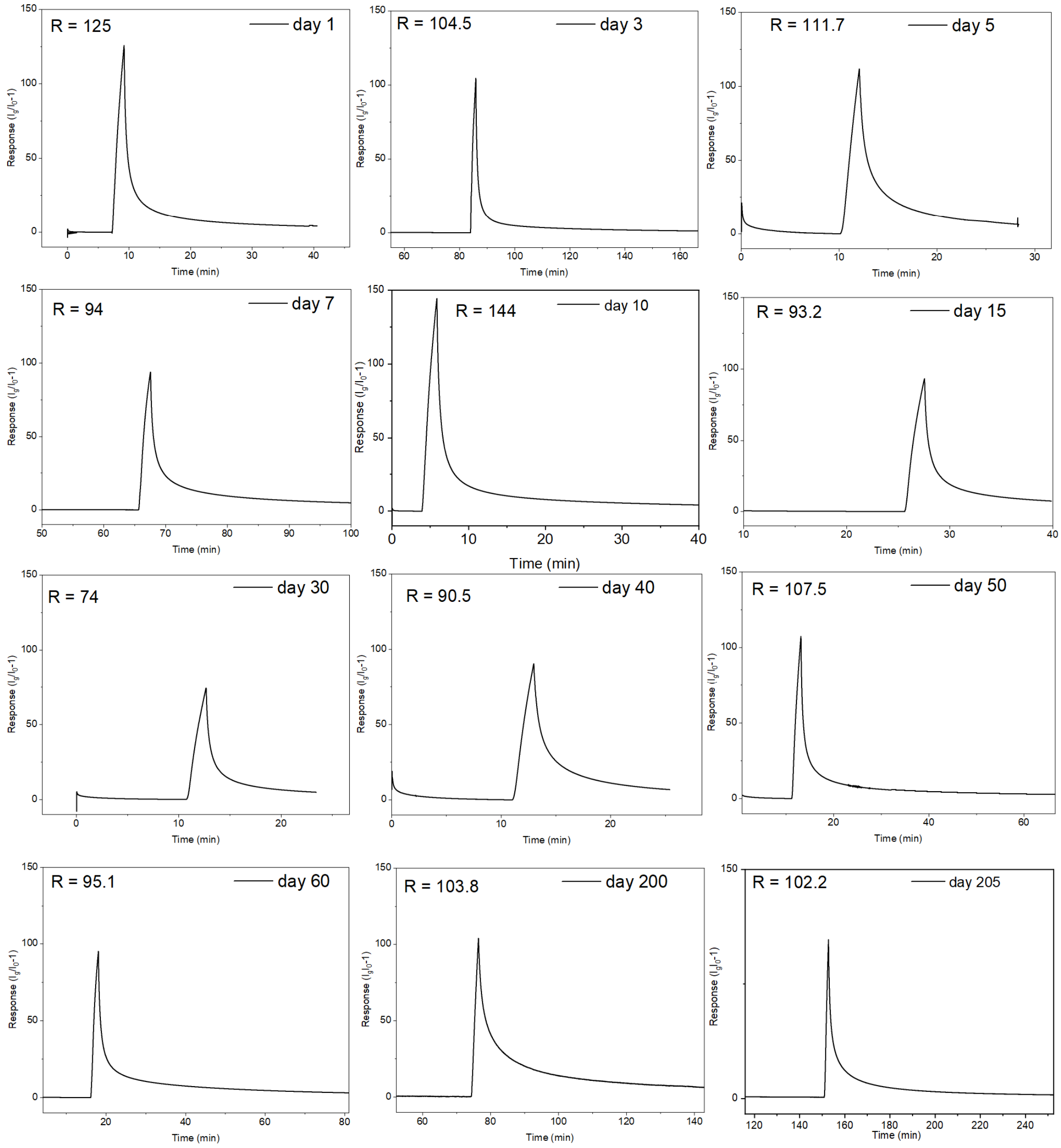

Figure S42. Response–recovery curves of the nanoCOF-366-A sensing device measured at different intervals during 205 days of aging, demonstrating the long-term stability of the sensor. For each measurement, the device was exposed to 1 ppm NO_2.

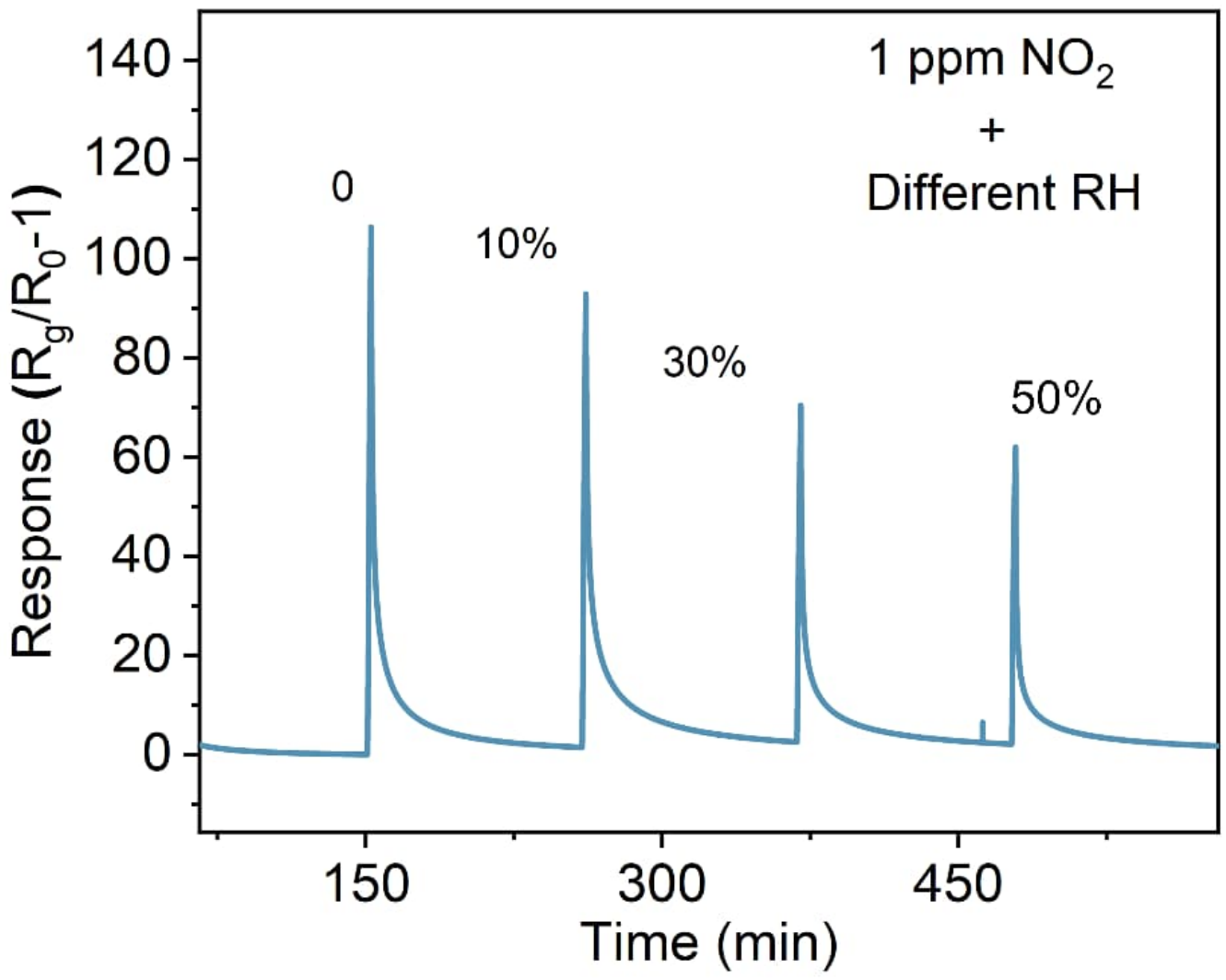

Figure S43. Response–recovery curves of the nanoCOF-366-A sensing device measured at different humidity.

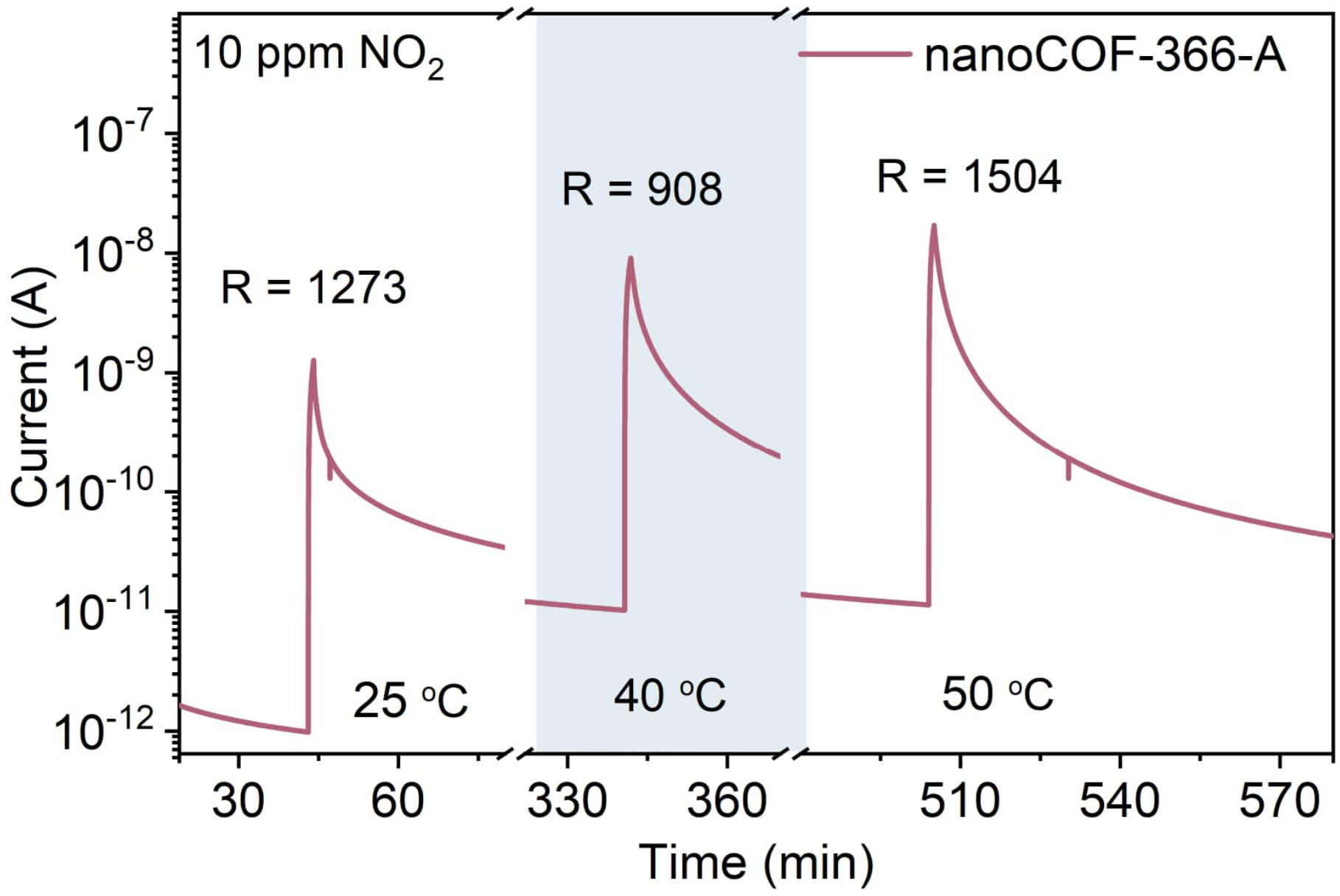

Figure S44. Response–recovery curves of the nanoCOF-366-A sensing device measured at different temperature.

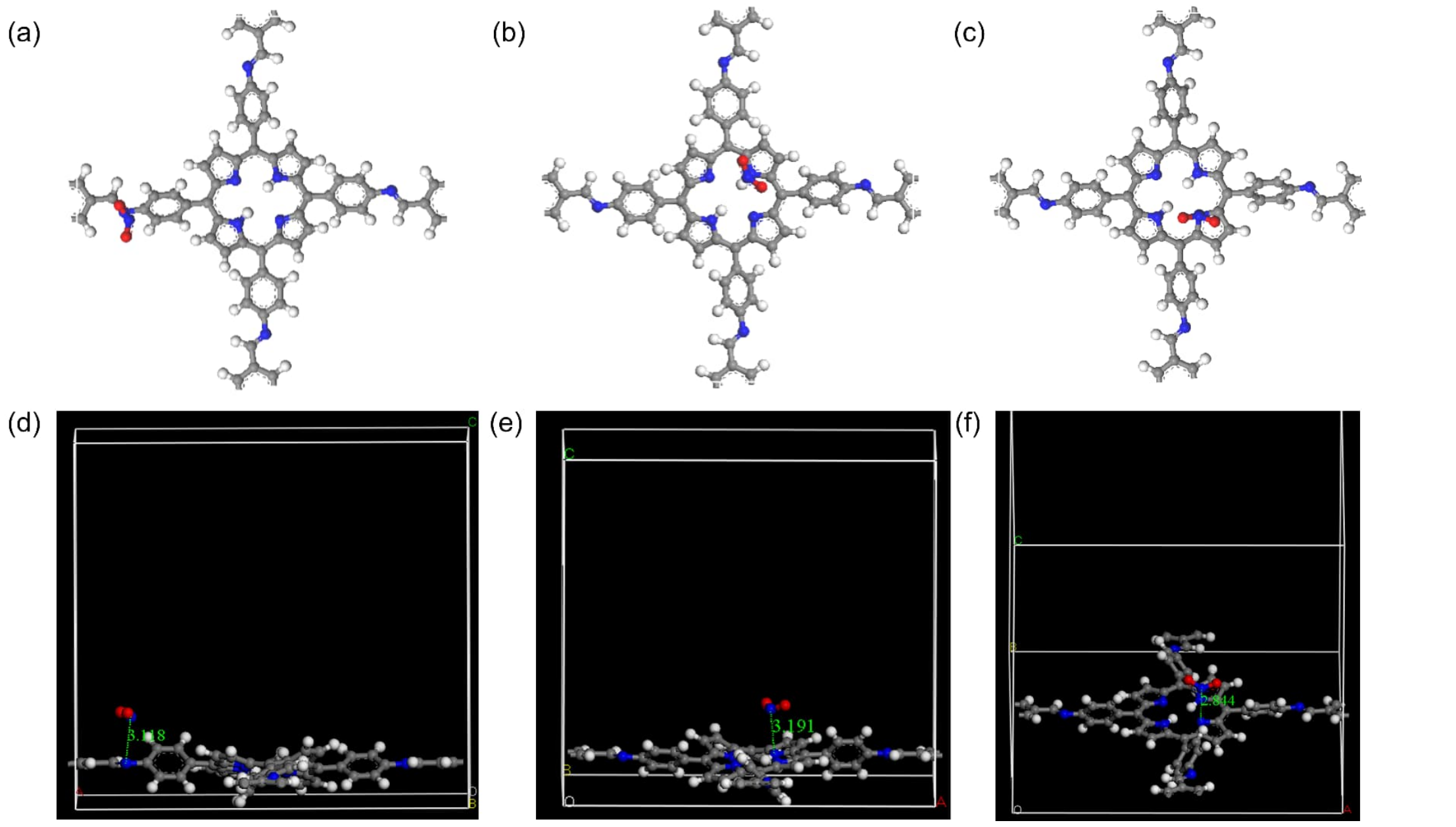

Figure S45. Top (a–c) and side (d–f) views of NO_2 adsorption configurations on the N sites of the imine linkages and porphyrin moieties of COF-366. The numbers indicate the distances (Å) between NO_2 molecules and N atoms of the imine and porphyrin units upon NO_2 adsorption. Color scheme: C, gray; H, white; N, blue; O, red.

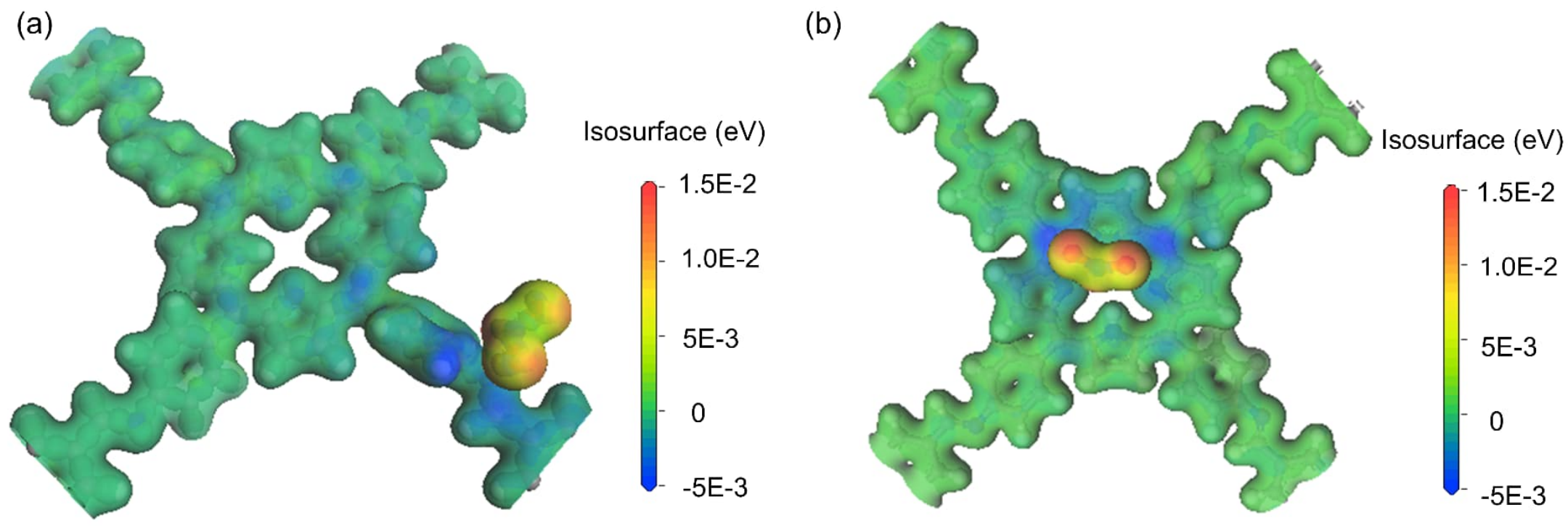

Figure S46. Electron density difference of NO_2 adsorbed on the imine linkages (left) and porphyrin moieties (right) of COF-366.

Table S1. Baseline current (I_0) and gas response current (I_g) for all three samples (nanoCOF-366, ST-COF-366, and COF-366-A)

**Sample**	**I_0**	**I_g**
ST-COF-366	4.9 pA	0.17 nA
nanoCOF-366	8.0 pA	0.67 nA
nanoCOF-366-A	1.6 pA	1.91 nA

Table S2. Comparison of NO_2 sensing performance of nanoCOF-366 with reported chemiresistive sensing materials.

No.	Material	Conc./ppm	Condition	Response	LOD	Refs
[1]	COF-CuNiPc	10	UV	11.50	5.4 ppb	10
[2]	COF-CuPc	10	UV	5.88	191.5 ppb	
[3]	COF-NiPc	10	UV	2.84	244.8 ppb	
[4]	H_2-TPCOF	10	RT	9.55	1.23 ppm	11
[5]	Cu-TPCOF	10	RT	11.2	1.14 ppm	
[6]	Co-TPCOF	10	RT	239.88	6.8 ppb	
[7]	Zn-TPCOF	10	RT	100	7.9 ppb	12
[8]	TiO_2@COF-316	10	VL	64.56	1.41 ppm	13
[9]	AgBMT	10	RT	1.31	5.3 ppb	14
[10]	TiO_2 @NH_2-MIL-125	10	Green Light	2	1 ppb	15
[11]	TAPB–PDA@Cs_2PdBr_6	10	RT	1.80	10 ppb	16
[12]	TpPa-1/Cs_2PdBr_6	10	RT	350	40 ppb	17
[13]	NiPc-CoTAA	10	RT	6.02	300 ppb	18
[14]	COF-Az	10	RT	0.59	0.42 ppm	19
[15]	COF-DC-8	10	RT	12.64	16 ppb	20
[16]	CON-10	10	200 ℃	72.5	20 ppb	21
[17]	T-2DP	10	RT	452.6	2.2 ppb	22
[18]	Vio-CTFs	10	RT	0.36	10 ppb	23
[19]	$(NH_2)_{1.24}$-UiO-66@TDCOF	10	VL	230	27.8 ppb	24
[20]	MIL-101(Cr) PEDOT	1/10	RT	~0.04/0.46	60 ppb	25

[21]	Au/ZIF-8	1/10	RT	~0.0079/0.034	190 ppb	26
[22]	Por-COF-Me	10	RT	21	19.39 ppb	
[23]	Por-COF-H	10	RT	71.7	15.3 ppb	27
[24]	POR-COF-F	10	RT	195	7.82 ppb	
[25]	FIR-120	1/10	VL	1.44/58	40 ppb	28
[26]	HIOTP-Ni	10	RT	4.05	0.21 ppm	29
[27]	CeO_2/CuO	10	100 ℃	4	0.6 ppb	30
[28]	porous P3HT film	10	RT	50	2.45 ppb	31
[29]	Cu2O/CuO-C	10	RT	1101	7 ppb	32
[30]	ZIF-8-8	10	350 ℃	30	–	33
[31]	Mg-MOFs-II	10	200 ℃	~1.24		34
[32]	NH_2-UiO-66	10	150 ℃	0.08	–	35
[33]	Cu-MOF-250	0.14/2	40 ℃	1.05/~1.36	~80 ppb	36
[34]	$Cu_3(HHTP)_2$	1	RT	0.05	–	37
[35]	Pd@$Cu_3(HHTP)_2$	1	RT	0.144	–	
[36]	Pt@$Cu_3(HHTP)_2$	1	RT	0.12	–	38
[37]	Pt@$Cu_3(HHTP)_2$	3	150 ℃	0.9	100 ppb	
[38]	$Cu_3(HHTP)_2$	5	RT (Dark)	0.8	–	
[39]	$Cu_3(HHTP)_2$	5	Blue light	0.69	–	39
[40]	Fe_2O_3-$Cu_3(HHTP)_2$	5	Blue light	~0.62	11 ppb	
[41]	ZnCo-ZIF/GN-120	100	RT	54.61	10 ppb	40
[42]	BP@MIL-53	1	70 ℃	~0.13	1 ppb	41
[43]	Pd1-cMOF	2	RT	0.09	–	42
[44]	CPs@CoHHTP 1:4	2.5	RT	28.63	0.25 ppm	
[45]	CPs@NiHITP 1:1	2.5	RT	22.82	0.25 ppm	43
[46]	CPs@NiHHTP	2.5	RT	17.16	0.25 ppm	

	8:1					
[47]	PCN-222-Cu	80 ppb	RT	0.8	0.93 ppb	44
[48]	NiO/CdS-CdO	100	150 ℃	214.	100 ppb	45
[49]	In-ZnO	100	175 ℃	45.	5 ppm	46
[50]	Cu_2O–CuO	1	RT	13.30	10 ppb	47
[51]	Graphene	1	RT	0.06	15 ppb	48
[52]	MoS_2/Graphene	1	200 ℃	0.12	50 ppb	49
[53]	TiO_2/Graphene	1.75	UV	3.30	70 ppb	50
[54]	3D TiO_2	1	UV	3.33	202 ppt	51
[55]	GaN/TiO_2	1	UV	0.13	1 ppb	52
[56]	ZnO	1	UV	6.10	2 ppb	53
[57]	ZnO	1	UV	7.08	20 ppb	54
[58]	rGO@ZnO_{1-x}	0.1	VL	4.66	<50 ppb	55
[59]	ZnO/rGO	4	125 ℃	56	-	56
[60]	Au@In_2S_3/In_2O_3	50	RT	1500	-	57
[61]	$MoSe_2$-rGO	4	RT	0.38	-	58
[62]	rGO@COF-V	5	RT	0.4	250 ppb	59
[63]	SA-TAPB COF	1	RT	20.	30 ppb	60
[64]	IM-x/P-y	1	60 ℃	2.4	0.1 ppm	61
[65]	H_2SO_4-doped Py-1P	5	RT	1141.4	<500 ppb (Experimental)	
[66]	H_3PO_4-doped Py-1P			3025.2		62
**[67]**	**nanoCOF-366**	**10**	**RT**	**92.6**	**11.9 ppb**	**This**
**[68]**	**nanoCOF-366-A**	**10**	**RT**	**1083**	**0.21 ppb**	**work**

References

(1) Wan, S.; Gándara, F.; Asano, A.; Furukawa, H.; Saeki, A.; Dey, S. K.; Liao, L.; Ambrogio, M. W.; Botros, Y. Y.; Duan, X.; Seki, S.; Stoddart, J. F.; Yaghi, O. M. Covalent Organic Frameworks with High Charge Carrier Mobility. *Chem. Mater.* **2011**, *23*, 4094-4097.

(2) Clark, S. J.; Segall, M. D.; Pickard, C. J.; Hasnip, P. J.; Probert, M. I. J.; Refson, K.; Payne, M. C. First principles methods using CASTEP. *Z. Kristallogr. Cryst. Mater.* **2005**, *220*, 567-570.

(3) Perdew, J. P.; Burke, K.; Ernzerhof, M. Generalized Gradient Approximation Made Simple. *Phys. Rev. Lett.* **1996**, *77*, 3865-3868.

(4) D Frenkel, B. S. *Understanding molecular simulation: from algorithms to applications*; Elsevier, 2023.

(5) Sun, H. COMPASS: An ab Initio Force-Field Optimized for Condensed-Phase ApplicationsOverview with Details on Alkane and Benzene Compounds. *J. Phys. Chem. B* **1998**, *102*, 7338-7364.

(6) Rappe, A. K.; Goddard, W. A., III. Charge equilibration for molecular dynamics simulations. *J. Chem. Phys.* **1991**, *95*, 3358-3363.

(7) Mulliken, R. S. Electronic Population Analysis on LCAO–MO Molecular Wave Functions. I. *J. Chem. Phys.* **1955**, *23*, 1833-1840.

(8) Delley, B. From molecules to solids with the DMol 3 approach. *J. Chem. Phys.* **2000**, *113*, 7756-7764.

(9) Ewald, P. P. Die Berechnung optischer und elektrostatischer Gitterpotentiale. *Ann. Phys.* **1921**, *369*, 253-287.

(10) Chen, X.; Zeng, M.; Yang, J.; Hu, N.; Duan, X.; Cai, W.; Su, Y.; Yang, Z. Two-Dimensional Bimetallic Phthalocyanine Covalent-Organic-Framework-Based Chemiresistive Gas Sensor for ppb-Level NO_2 Detection. *Nanomaterials* **2023**, *13*,

1660.

(11) Liu, M.; Chen, Y. J.; Huang, X.; Dong, L. Z.; Lu, M.; Guo, C.; Yuan, D.; Chen, Y.; Xu, G.; Li, S. L.; Lan, Y. Q. Porphyrin-Based COF 2D Materials: Variable Modification of Sensing Performances by Post-Metallization. *Angew. Chem. Int. Ed.* **2022**, *61*.

(12) Chen, Y.-J.; Liu, M.; Chen, J.; Huang, X.; Li, Q.-H.; Ye, X.-L.; Wang, G.-E.; Xu, G. Dangling bond formation on COF nanosheets for enhancing sensing performances. *Chem. Sci.* **2023**, *14*, 4824-4831.

(13) Chen, Y.-J.; Wen, Y.-Y.; Li, W.-H.; Fu, Z.-H.; Wang, G.-E.; Xu, G. TiO_2@COF Nanowire Arrays: A “Filter Amplifier” Heterojunction Strategy to Reverse the Redox Nature. *Nano Lett.* **2023**, *23*, 3614-3622.

(14) Gong, W.; Pan, Y.; Shi, Y.; Wang, G.; Xu, G. Imidazole-amino modified organic-metal chalcogenide for enhanced recyclability and recovery rate in gas sensing. *Sens. Actuator B-Chem.* **2025**, *445*.

(15) Deng, W.-H.; Zhang, M.-Y.; Li, C.-S.; Yao, M.-S.; Xu, G. Energy-Level Alignment at TiO_2@NH_2-MIL-125 Interface for High-Performance Gas Sensing. *Angew. Chem. Int. Ed.* **2025**, *64*, e202419195.

(16) Ye, W.; Li, M.; Li, G.; Jiang, L.; Tian, S.; Dong, S.; Xu, Q.; Chen, D.; Nazeeruddin, M. K.; Dyson, P. J.; Abate, A.; Lu, J.-M. Covalent Organic Framework-Enhanced Metal Halide Perovskites for Selective and Sensitive Gas Sensing. *Adv. Funct. Mater.* **2025**, *35*, 2418897.

(17) Ye, W.; Zhao, L.; Lin, H.-Z.; Ding, L.; Cao, Q.; Chen, Z.-K.; Wang, J.; Sun, Q.-M.; He, J.-H.; Lu, J.-M. Halide Perovskite glues activate two-dimensional covalent organic framework crystallites for selective NO_2 sensing. *Nat. Commun.* **2023**, *14*, 2133.

(18) Yue, Y.; Cai, P.; Xu, X.; Li, H.; Chen, H.; Zhou, H. C.; Huang, N. Conductive metallophthalocyanine framework films with high carrier mobility as efficient chemiresistors. *Angew. Chem. Int. Ed.* **2021**, *60*, 10806-10813.

(19) Feng, B.; Chen, X.; Yan, P.; Huang, S.; Lu, C.; Ji, H.; Zhu, J.; Yang, Z.; Cao, K.; Zhuang, X. Isomeric Dual-Pore Two-Dimensional Covalent Organic Frameworks. *J. Am. Chem. Soc.* **2023**, *145*, 26871-26882.

(20) Meng, Z.; Stolz, R. M.; Mirica, K. A. Two-dimensional chemiresistive covalent organic framework with high intrinsic conductivity. *J. Am. Chem. Soc.* **2019**, *141*, 11929-11937.

(21) Ko, W. C.; Kim, M.-S.; Kwon, Y. J.; Jeong, J.; Kim, W. R.; Choi, H.; Park, J. K.; Jeong, Y. K. Correction: Two-dimensional semiconducting covalent organic nanosheets for highly sensitive and stable NO_2 sensing under humid conditions. *J. Mater. Chem. A* **2020**, *8*, 21882-21882.

(22) Yang, K.; Yuan, W.; Hua, Z.; Tang, Y.; Yin, F.; Xia, D. Triazine-based two-dimensional organic polymer for selective NO_2 sensing with excellent performance. *ACS Appl. Mater. Interfaces* **2019**, *12*, 3919-3927.

(23) Niu, F.; Zhu, J.-L.; Xu, M.-K.; Ding, Y.; Tao, L.-M.; Jin, J. Viologen-based covalent triazine frameworks for NO_2 sensing at room temperature. *Sens. Actuator B-Chem.* **2022**, *352*, 131045.

(24) Chen, Y.-J.; Xu, Y.-M.; Ye, X.-L.; Luo, Z.-P.; Zhu, S.-P.; Li, K.-F.; Lu, J.-F.; Wang, G.-E.; Xu, G. Strategic energy-level modulation in porous heterojunctions: advancing gas sensing through Type-I to Type-II transitions. *Nat. Commun.* **2025**, *16*, 6634.

(25) Le Ouay, B.; Boudot, M.; Kitao, T.; Yanagida, T.; Kitagawa, S.; Uemura, T. Nanostructuration of PEDOT in porous coordination polymers for tunable porosity and conductivity. *J. Am. Chem. Soc.* **2016**, *138*, 10088-10091.

(26) Li, P.; Zhan, H.; Tian, S.; Wang, J.; Wang, X.; Zhu, Z.; Dai, J.; Dai, Y.; Wang, Z.; Zhang, C. Sequential ligand exchange of coordination polymers hybridized with in situ grown and aligned Au nanowires for rapid and selective gas sensing. *ACS Appl. Mater. Interfaces* **2019**, *11*, 13624-13631.

(27) Li, Z.; Shi, W.; Seo, J.-M.; Jung, H.; Kim, M.; Wang, Z.; Jin, Y.; Li, C.; Yoo, J.-W.;

Lee, Z.; Kang, B.; Lee, K. H.; Baek, J.-B. Tuning Electronic Skeleton Properties of Porphyrin Covalent Organic Frameworks (COFs) for High-Performance Selective Gas Chemiresistive Sensing. *Nano Lett.* **2026**, *26*, 42-50.

(28) Li, H.-Z.; Pan, Y.; Li, Q.; Lin, Q.; Lin, D.; Wang, F.; Xu, G.; Zhang, J. Rationally designed titanium-based metal–organic frameworks for visible-light activated chemiresistive sensing. *J. Mater. Chem. A* **2023**, *11*, 965-971.

(29) Chen, P.; Su, X.; Wang, C.; Zhang, G.; Zhang, T.; Xu, G.; Chen, L. Two-Dimensional Conjugated Metal-Organic Frameworks with Large Pore Apertures and High Surface Areas for NO_2 Selective Chemiresistive Sensing. *Angew. Chem. Int. Ed.* **2023**, *135*, e202306224.

(30) Fang, H.; Shang, E.; Wang, D.; Ma, X.; Zhao, B.; Han, C.; Zheng, C. A chemiresistive ppt level NO_2 gas sensor based on CeO_2 nanoparticles modified CuO nanosheets operated at 100° C. *Sens. Actuator B-Chem.* **2023**, *393*, 134277.

(31) Liu, C.; Wu, M.; Gao, L.; Liu, H.; Yu, J. Nanoporous polymer films based on breath figure method for stretchable chemiresistive NO_2 gas sensors. *Sens. Actuator B-Chem.* **2022**, *371*, 132540.

(32) Li, N.; Hu, J.; Li, J.; Cheng, M.; Wei, T.; Liu, Q.; Wang, R.; Li, W.; Ling, Y.; Zhang, Y. Cu-MOF-74-derived Cu_2O/CuO-C nanocomposite as an effective chemiresistive sensor for detection of NO_2 at room temperature. *J. Alloys Compd.* **2024**, *976*, 173074.

(33) Zhan, M.; Hussain, S.; AlGarni, T. S.; Shah, S.; Liu, J.; Zhang, X.; Ahmad, A.; Javed, M. S.; Qiao, G.; Liu, G. Facet controlled polyhedral ZIF-8 MOF nanostructures for excellent NO_2 gas-sensing applications. *Mater. Res. Bull.* **2021**, *136*, 111133.

(34) Lee, J.-H.; Nguyen, T.-B.; Nguyen, D.-K.; Kim, J.-H.; Kim, J.-Y.; Phan, B. T.; Kim, S. S. Gas sensing properties of Mg-incorporated metal–organic frameworks. *Sensors* **2019**, *19*, 3323.

(35) DMello, M. E.; Sundaram, N. G.; Singh, A.; Singh, A. K.; Kalidindi, S. B. An amine functionalized zirconium metal–organic framework as an effective

chemiresistive sensor for acidic gases. *Chem. Commun.* **2019**, *55*, 349-352.

(36) Arul, C.; Moulaee, K.; Donato, N.; Iannazzo, D.; Lavanya, N.; Neri, G.; Sekar, C. Temperature modulated Cu-MOF based gas sensor with dual selectivity to acetone and NO_2 at low operating temperatures. *Sens. Actuator B-Chem.* **2021**, *329*, 129053.

(37) Koo, W. T.; Kim, S. J.; Jang, J. S.; Kim, D. H.; Kim, I. D. Catalytic metal nanoparticles embedded in conductive metal–organic frameworks for chemiresistors: highly active and conductive porous materials. *Adv. Sci.* **2019**, *6*, 1900250.

(38) Kim, J.-O.; Koo, W.-T.; Kim, H.; Park, C.; Lee, T.; Hutomo, C. A.; Choi, S. Q.; Kim, D. S.; Kim, I.-D.; Park, S. Large-area synthesis of nanoscopic catalyst-decorated conductive MOF film using microfluidic-based solution shearing. *Nat. Commun.* **2021**, *12*, 4294.

(39) Jo, Y.-M.; Lim, K.; Yoon, J. W.; Jo, Y. K.; Moon, Y. K.; Jang, H. W.; Lee, J.-H. Visible-light-activated type II heterojunction in Cu_3(hexahydroxytriphenylene)$_2$/Fe_2O_3 hybrids for reversible NO_2 sensing: critical role of π–π* transition. *ACS Cent. Sci.* **2021**, *7*, 1176-1182.

(40) He, L.; Zhang, W.; Wu, H.; Zhao, Y. Zn–Co zeolitic imidazolate framework nanoparticles intercalated in graphene nanosheets for room-temperature NO_2 sensing. *ACS Appl. Nano Mater.* **2021**, *4*, 3998-4006.

(41) Han, B.; Duan, Z.; Xu, J.; Zhu, Y.; Xu, Q.; Wang, H.; Tai, H.; Weng, J.; Zhao, Y. The art of integrated functionalization: super stable black phosphorus achieved through metal-organic framework coating. *Adv. Funct. Mater.* **2020**, *30*, 2002232.

(42) Park, C.; Shin, H.; Jeon, M.; Cho, S.-H.; Kim, J.; Kim, I.-D. Single-atom catalysts in conductive metal–organic frameworks: enabling reversible gas sensing at room temperature. *ACS Nano* **2024**, *18*, 26066-26075.

(43) Roh, H.; Kim, D. H.; Cho, Y.; Jo, Y. M.; Del Alamo, J. A.; Kulik, H. J.; Dincă, M.; Gumyusenge, A. Robust chemiresistive behavior in conductive polymer/MOF composites. *Adv. Mater.* **2024**, *36*, 2312382.

(44) Zhang, Y.; Liu, Q.; Sun, Q.; Li, H.; Shen, J.; Liu, H.; Chen, W.; Zhang, Y.; Chen, Y. Metalloporphyrin-Based Metal–Organic Frameworks for the Ultrasensitive Chemiresistive Detection of NO_2: Effect of the Central Metal on Tuning the Sensing Performance. *ACS Sens.* **2023**, *8*, 4353-4363.

(45) Hassan, M.; Liang, Z.; Liu, S.; Hussain, S.; Qiao, G.; Liu, G. Temperature-driven n-to p-type transition of a chemiresistive NiO/CdS-CdO NO_2 gas sensor. *Sens. Actuator B-Chem.* **2024**, *398*, 134755.

(46) Patil, V.; Dalavi, D.; Dhavale, S.; Vanalakar, S.; Tarwal, N.; Kalekar, A.; Kim, J.; Patil, P. Indium doped ZnO nanorods for chemiresistive NO_2 gas sensors. *New J. Chem.* **2022**, *46*, 7588-7597.

(47) Wang, W.; Zhang, Y.; Zhang, J.; Li, G.; Leng, D.; Gao, Y.; Gao, J.; Lu, H.; Li, X. Metal–organic framework-derived Cu_2O–CuO octahedrons for sensitive and selective detection of ppb-level NO_2 at room temperature. *Sens. Actuator B-Chem.* **2021**, *328*, 129045.

(48) Paul, R. K.; Badhulika, S.; Saucedo, N. M.; Mulchandani, A. Graphene nanomesh as highly sensitive chemiresistor gas sensor. *Anal. Chem.* **2012**, *84*, 8171-8178.

(49) Long, H.; Harley-Trochimczyk, A.; Pham, T.; Tang, Z.; Shi, T.; Zettl, A.; Carraro, C.; Worsley, M. A.; Maboudian, R. High surface area MoS_2/graphene hybrid aerogel for ultrasensitive NO_2 detection. *Adv. Funct. Mater.* **2016**, *26*, 5158-5165.

(50) Giampiccolo, A.; Tobaldi, D. M.; Leonardi, S. G.; Murdoch, B. J.; Seabra, M. P.; Ansell, M. P.; Neri, G.; Ball, R. J. Sol gel graphene/TiO_2 nanoparticles for the photocatalytic-assisted sensing and abatement of NO_2. *Appl. Catal. B.* **2019**, *243*, 183-194.

(51) Cho, D.; Suh, J. M.; Nam, S. H.; Park, S. Y.; Park, M.; Lee, T. H.; Choi, K. S.; Lee, J.; Ahn, C.; Jang, H. W. Optically activated 3D thin-shell TiO_2 for super-sensitive chemoresistive responses: toward visible light activation. *Adv. Sci.* **2021**, *8,* 2001883.

(52) Khan, M. A. H.; Thomson, B.; Debnath, R.; Rani, A.; Motayed, A.; Rao, M. V.

Reliable anatase-titania nanoclusters functionalized GaN sensor devices for UV assisted NO_2 gas-sensing in ppb level. *Nanotechnology* **2020**, *31*, 155504.

(53) Wang, J.; Yu, M.; Xia, Y.; Li, X.; Yang, C.; Komarneni, S. On-chip grown ZnO nanosheet-array with interconnected nanojunction interfaces for enhanced optoelectronic NO_2 gas sensing at room temperature. *J. Colloid Interface Sci.* **2019**, *554*, 19-28.

(54) Wang, J.; Shen, Y.; Li, X.; Xia, Y.; Yang, C. Synergistic effects of UV activation and surface oxygen vacancies on the room-temperature NO_2 gas sensing performance of ZnO nanowires. *Sens. Actuator B-Chem.* **2019**, *298*, 126858.

(55) Geng, X.; Zhang, C.; Olivier, M.-G.; Debliquy, M. Room Temperature NO_2 Responses of Visible-Light Activated Nanosheet rGO@ZnO_{1-x} Sensors. *Proceedings*, 2017, *1*, 411.

(56) Li, D.; Lu, J.; Zhang, X.; Jin, D.; Jin, H. Engineering of ZnO/rGO towards NO_2 gas detection: ratio modulated sensing type and heterojunction determined response. *Nanomaterials* **2023**, *13*, 917.

(57) Wang, B.; Sun, Y.; Dong, Y.; Hou, Y.; Lu, Z.; Wei, Z.; Zhang, W.; Suematsu, K.; Hu, J. Chemiresistive flexible gas sensor for NO_2 sensing at room-temperature using in situ constructed Au@ In_2S_3/In_2O_3 hybrid microflowers. *Sens. Actuator B-Chem.* **2025**, *422*, 136666.

(58) Shakya, P.; Khan, M. S.; Kumar, S.; Jhankal, D.; Verma, N.; Sachdev, K. Understanding the gas sensing mechanism of $MoSe_2$ nanoroses and rGO composite-based chemiresistive sensors for room temperature NO2 detection. *Chem. Eng. J.* **2025**, *503*, 158178.

(59) Choi, J.; Kim, T.; Li, H.; Jung, H.-T.; Zhao, D. Gas sensors with two-dimensional rGO@ COF composite materials for fast NO_2 detection under room temperature. *ACS Appl. Mater. Interfaces* **2023**, *15*, 44119-44126.

(60) Wang, J.; Cao, Q.; Cheng, X.-F.; Ye, W.; He, J.-H.; Lu, J.-M. Moisture-insensitive

and highly selective detection of NO_2 by ion-in-conjugation covalent organic frameworks. *ACS Sens.* **2022**, *7*, 3782-3789.

(61) Park, H.; Kim, D. H.; Ma, B. S.; Shin, E.; Kim, Y.; Kim, T. S.; Kim, F. S.; Kim, I. D.; Kim, B. J. High-Performance, Flexible NO_2 Chemiresistors Achieved by Design of Imine-Incorporated n-Type Conjugated Polymers. *Adv. Sci.* **2022**, *9*, 2200270.

(62) Q. Liu, X. Shi, H. Zheng, M. Chen, W. Li, A. Mukherjee, H. Li, C. Kang, Z. Wang, J. Ren, W. Zhao, J. Choi, D. Zhao, Acidic Electron Acceptors in Imine-Linked Covalent Organic Framework for Enhanced Gas Sensing With Field-Effect Transistor Evaluation, *Angew. Chem. Int. Ed*., **2026**, e5511899.
